# Abrogation of PIK3CA or PIK3R1 reduces proliferation, migration, and invasion in glioblastoma multiforme cells

**DOI:** 10.18632/oncotarget.346

**Published:** 2011-11-05

**Authors:** Genevieve L. Weber, Marie-Odile Parat, Zev A. Binder, Gary L. Gallia, Gregory J. Riggins

**Affiliations:** ^1^ Ludwig Collaborative Laboratory, Department of Neurosurgery, Johns Hopkins University School of Medicine, Baltimore, MD; ^2^ School of Pharmacy, University of Queensland, Australia

**Keywords:** glioblastoma, PIK3CA, PIK3R1, invasion, PI3K, pathway analysis

## Abstract

Glioblastoma multiforme (GBM) is a highly invasive and deadly brain tumor. Tumor cell invasion makes complete surgical resection impossible and reduces the efficacy of other therapies. Genome-wide analyses of mutations, copy-number changes, and expression patterns have provided new insights into genetic abnormalities common in GBM. We analyzed published data and identified the invasion and motility pathways most frequently altered in GBM. These were most notably the focal adhesion and integrin signaling, and extracellular matrix interactions pathways. We mapped alterations in each of these pathways and found that they included the catalytic *PIK3CA* and regulatory *PIK3R1* subunit genes of the class IA PI3K. Knockdown of either of these genes separately in GBM cell lines by lentiviral-mediated shRNA expression resulted in decreased proliferation, migration, and invasion in all lines tested. FAK activity was reduced by knockdown of either *PIK3CA* or *PIK3R1*, and MMP2 levels were reduced by knockdown of *PIK3R1*. We conclude that *PIK3R1*, like *PIK3CA*, is a potential therapeutic target in GBM and that it also influences tumor cell growth and motility.

## INTRODUCTION

Glioblastoma multiforme is the most common primary brain malignancy, with a current median survival of approximately 15 months in patients with newly diagnosed disease following treatment with surgery, chemotherapy and radiotherapy [1]. The invasive nature of GBM has been frequently implicated as a key feature of GBM's resistance to therapy [2-5]. Targeting pathways involved in the invasive phenotype of the tumor could be an important component of successful treatment in combination with strategies that more directly target the cancer cells.

The sequencing of 20,661 gene coding regions in multiple glioblastoma tumors was a significant milestone in understanding the genetic basis of GBM [6]. In this analysis, Parsons, et al utilized automated sequencing approaches and Illumina SNP arrays to identify mutations and copy number changes, respectively, in genes in 22 glioblastomas [6]. Genes found to harbor alterations in at least 2/22 tumors, and which had mutation frequencies of >10 mutations per Mb of DNA sequenced, were subsequently re-sequenced in an additional 83 tumors as validation. This sequencing of 22 GBM exomes, along with detection of genomic amplifications and homozygous deletions, likely identifies the majority of the mutated genes driving tumor formation and progression in glioblastoma.

One challenge has been to identify the genetic alterations that occur during tumor development that are functionally involved in driving tumor formation and progression. Carter et al developed the Cancer-specific High-throughput Annotation of Somatic Mutations (CHASM) program to systematically identify mutations in tumors that are likely to drive the cancer phenotype [7]. The CHASM program utilizes 49 predictive features to estimate the likelihood that a specific mutation in question is a driver, rather than a passenger mutation [7]. This group applied CHASM to a list of 607 missense mutations found by Parsons et al in GBM [6, 7]. The result was a stringent list of 24 mutations (8% of the total mutation list) that have a much higher probability of being functionally involved in GBM pathogenesis, and included alterations found in the *PTEN, TP53, IDH1*, and *PIK3CA* genes, among others.

The mutations of functional consequence in GBM are responsible for many aspects of its malignant phenotype, including cellular invasion. Glioblastoma cells exhibit significantly increased motility and invasive potential as compared to many brain tumors of lower grade [8, 9]. Tumor cell invasion results in an inability to cure the tumor via surgery alone, and invading tumor cells are more resistant to apoptosis, radiation and certain chemotherapies [5, 8-10]. It is likely that anti-invasion/motility therapy might render these cells more susceptible to apoptosis-based chemotherapeutics.

Glioblastoma cell invasion likely occurs through multiple mechanisms, beginning with the degradation of surrounding matrix proteins by proteases and proteinases in order to create a cavity through which a tumor cell can migrate [4, 9, 11]. Invading cells must detach from neighboring cells and matrix components in order to become motile [9, 11]. Cells can then move through healthy brain tissue in a receptor-mediated fashion that requires receptor turnover, including the formation and degradation of focal adhesions, and cytoskeletal rearrangements [9, 12]. Cell attachments, along with receptor and focal adhesion turnover and cytoskeleton changes, are controlled in part by complex interactions between integrins, receptor tyrosine kinases, and pathways such as focal adhesion kinase (FAK) and phosphatidylinositol 3-kinase (PI3K) signaling [12-16]. Several ECM components themselves, including laminin and fibronectin, have been shown to be overexpressed in tumors, and downregulation of these components reduces invasion and migration of glioblastoma cells [17-21]. The rate of proliferation of invading tumor cells is often significantly decreased when compared to cells in the main tumor mass [8, 9]. Targeting both proliferating and migrating cells is likely essential for an effective therapy.

The PI3K cascade is an important pathway known to be involved in proliferation, invasion, and migration in cancer [22-24]. Class IA PI3Ks are heterodimers of a p85 regulatory subunit and a p110 catalytic subunit [25, 26]. Three catalytic subunits exist, and are designated p110α, p110β, and p110δ. Five class IA PI3K regulatory subunits include p85α, p85β, p50α, p55α, and p55γ. The p85α regulatory and p110α catalytic subunits are the most highly expressed and form heterodimers most commonly [26]. Upon localization of the heterodimer to the plasma membrane via binding of Src homology 2 (SH2) domains on the regulatory subunit to activated receptor tyrosine kinases, the regulatory subunit releases its inhibitory control of the catalytic subunit, and the catalytic subunit comes in close contact with its lipid substrates. The catalytic subunit phosphorylates phosphatidylinositol 4,5-bisphosphate (PIP2), converting it to phosphatidylinositol 3,4,5-triphosphate (PIP3). PIP3 can activate several downstream signaling cascades, including the Akt and mTOR pathways, which are involved in proliferation and cell survival. PTEN activity converts PIP3 to PIP2, thus regulating the level of activation of these downstream pathways. Constitutive activation of the Akt and/or mTOR pathways and loss of function of PTEN can each contribute to tumor progression [27, 28].

The *PIK3CA* gene, which encodes the class IA PI3K catalytic subunit p110α, has been found to harbor mutations in several cancers [29-31]. Approximately 80% of p110α mutations cluster in “hot spots” in the helical (E542K and E545K in exon 9) and kinase (H1047R in exon 20) domains, and were considered to be likely drivers of cancer formation and/or progression by Parsons et al and Carter et al [6, 7, 29]. However, the majority of mutations discovered in *PIK3CA*, regardless of their location, have been shown to constitutively activate the catalytic activity of p110α [32].

The *PIK3R1* gene, which encodes the p85α, p55α, and p50α class IA PI3K regulatory subunits, was found to be mutated in glioblastoma tumors by Parsons et al and amplified in GBMs in multiple studies [6, 33]. *PIK3R1* is also mutated in other human cancers, including colorectal cancer, breast cancer, ovarian cancer, and endometrial cancer [34-36].

In many cases, *PIK3R1* mutations are thought to act through the catalytic subunit [34, 37, 38]. p85α inhibits the activity of the catalytic subunit by binding of its N-terminal SH2 domain to the N-terminus of the catalytic subunit [39]. Interaction with a regulatory subunit is also important for the stability of the catalytic subunit, which may degrade more rapidly in an unbound state [25]. A significant number of mutations documented in *PIK3R1* are located in the inter-SH2 or N-terminal SH2 domain of p85α, which may result in a regulatory subunit that no longer inhibits activity, but still maintains stability, of the catalytic subunit [40, 41]. Since p85α regulates the class IA PI3K catalytic subunit, mutations in *PIK3R1* may potentially activate invasion.

Our hypothesis for this work is that there are common mutations that activate a defined set of pathways that are responsible for the invasive properties in glioblastoma cells. We performed an in-depth informatics analysis to search for invasion and migration-related pathways that are enriched with mutations in GBM. With the recent availability of a well defined set of mutations responsible for GBM, we hope to determine the likely mutational basis responsible for at least part of GBM's invasive phenotype. We mapped many of the GBM mutations to pathways that may influence the invasive or migratory phenotype of this tumor, including focal adhesion signaling, integrin signaling, and extracellular component and receptor signaling. We show, as have multiple lines of pre-existing evidence that the PI3K signaling cascade is involved in each of these pathways and is likely to play an important role in both the proliferative and migratory/invasive phenotypes observed in GBM cells. shRNA-mediated knockdown of either the *PIK3CA* or *PIK3R1* gene resulted in reduced proliferation, migration, and invasion in glioblastoma cell lines, and in the case of the *PIK3R1* gene these effects are independent of the Akt pathway. Our studies suggest for the first time that *PIK3R1* may play an important role in the invasive and migratory capabilities of GBM cells, and that the p85α product of this gene may be an effective anti-invasion target.

## RESULTS

### Invasion and migration-related pathways are mutated in GBM

Using GBM sequencing and genomic copy number data, we sought statistically significant invasion and motility-related pathways enriched for genes mutated in GBM. A similar analysis was performed using gene expression data from the same tumors sequenced for mutation.

Our analyses included a comprehensive list of 703 genes mutated in GBM (Table S2), and a smaller more stringent list of 59 high probability driver mutations (Table S3) developed from the combined findings of Parsons et al [6], Carter et al [7], and Rao et al [33]. Transcript counts produced by next generation sequencing, a variation of Serial Analysis of Gene Expression (SAGE), were analyzed from Parsons et al [6]. This gene expression data covered 20,619 curated genes in 16 human tumors. Mutation data was analyzed using Ingenuity Pathway Analysis and Partek Genomics Suite, while SAGE data was studied using Spotfire DecisionSite. The use of Partek and Ingenuity simultaneously increases the validity and significance of our analysis, given that these programs produce results based upon algorithms that are complementary, but do not overlap.

Using the expanded and stringent mutated gene lists, we searched for enriched ontologies, based upon molecular function, cellular component, and biological process as defined by the Gene Ontology Consortium, using Partek Genomics Suite. Additionaly, we searched for enriched canonical pathways using Ingenuity Pathway Analysis, which ranks genes based on their function rather than inclusion within an ontological group. Both programs generated lists of pathways altered in GBM, each of which was assigned statistical significance (p-value greater than or equal to 0.5) (Tables S4-S7). We found that many of the statistically significant frequently mutated pathways were involved directly in the invasive or migrational phenotype in GBM (Tables S8, S9). Within these pathways, we also analyzed SAGE expression data to determine the presence of genes up- or down-regulated by 5-fold or greater. We mapped alterations in several of these enriched pathways, including focal adhesion signaling (Fig. S1), integrin signaling (Fig. S2), and extracellular matrix interactions (Fig. S3).

### Class IA PI3K plays a role in most invasion and migration-related pathways in GBM

**Figure 1:**
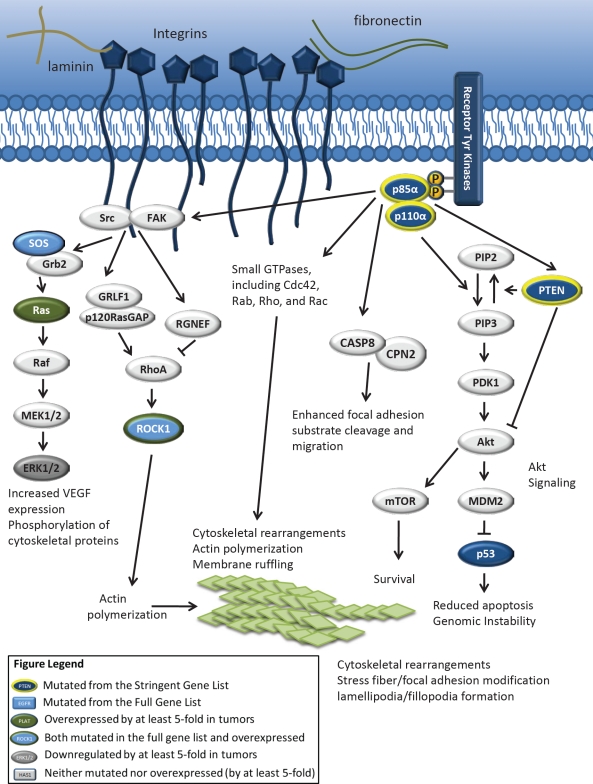
The PI3K pathway is central to proliferation, migration, and invasion in GBM cells Upon activation at the plasma membrane, the catalytic subunit of class IA PI3Ks phosphorylates phosphatidylinositol 4,5-bisphosphate (PIP2), converting it to phosphatidylinositol 3,4,5-triphosphate (PIP3). This results in the activation of several downstream pathways, including the Akt and mTOR pathways, which leads to increased proliferation and survival. PTEN activity converts PIP3 to PIP2. Interestingly, the p85α regulatory subunit has been shown to bind PTEN directly and enhance its lipid phosphatase activity [62]. This subunit may also be involved in the activation of migration and invasion-promoting pathways, possibly through the activation of small GTPases such as Cdc42 and Rac [23, 63]. p85α may interact with caspase 8 (in a non-apoptotic function of this caspase) to increase cell motility and Rac activation [55]. p85α has also been shown to interact with FAK, and this interaction correlates with FAK activation [14].

In studying the genes involved in the mutation-enriched invasion and migration-related pathways, we found that the class IA PI3K subunit genes *PIK3CA* and *PIK3R1* were central signal transducers in essentially all of the pathways studied (Fig. 1, S3, S4). *PIK3CA* is mutated in approximately 10% of GBMs and, *PIK3R1* is mutated in approximately 8% [29, 31, 35, 42]. The role of *PIK3CA* in tumor cell proliferation is well-studied as compared to its role in invasion. Examination of the original gene list (703 mutated genes) and the stringent gene list (59 likely driver mutations) produced results that were typically in agreement (Tables S4-S9). Results (p-values) will be discussed in terms of the stringent gene list analysis, due to the higher likelihood that these mutations are drivers of GBM formation. When this list was analyzed in Ingenuity Pathway Analysis, *PIK3CA* and *PIK3R1* were found to be involved in several important motility pathways, including focal adhesion (p<0.0001), integrin (p = 0.0004), Rac (p = 0.0046), and axonal guidance signaling (p = 0.0065). Similarly, when this list was analyzed in Partek Genomics Suite, both subunits were found to be involved in regulation of cell adhesion (p<0.0001), cell motion (p<0.0001), focal adhesion formation (p<0.0001), and the integrin-mediated signaling pathway (p = 0.0019). The results of analyses in Partek and Ingenuity, which use complementary algorithms but do not overlap, were in agreement in terms of the significance of the invasion and migration-related pathways that are mutated/aberrantly expressed in GBM, and in the involvement of *PIK3CA* and *PIK3R1* in these pathways. The PI3K cascade itself was found to be statistically significantly altered in GBM by both the Ingenuity (p<0.0001) and Partek programs (p<0.0001).

### The SKMG26, HCT116, and 081024 cell lines harbor *PIK3CA* mutations

In determining the mutational status of the *PIK3CA* gene in our cell lines, we sequenced the 10 most commonly mutated exons (1, 2, 4, 5, 7, 9, 12, 13, 18, 20) [6, 29, 31]. One glioblastoma line, SKMG26, and one colorectal cancer line, HCT116, have been previously sequenced. SKMG26 and HCT116 lines each bear a single somatic mutation in the kinase domain of the catalytic subunit (exon 20) [29, 31]. These mutations induce H1047Y and H1047R amino acid changes, respectively [29, 31]. In SKMG26 cells, this constitutes a change from a hydrophilic to an intermediate side chain at amino acid 1047. In HCT116 cells, amino acid 1047 remains hydrophilic. We confirmed the presence of these mutations in both cell lines (data not shown). Previous work by Samuels et al found that knockdown of the wild-type *PIK3CA* allele in HCT116 lines increased proliferation and invasion in this cell line as compared to the parental line under the condition of serum starvation [22]. Similarly, removal of the mutant allele resulted in significantly decreased proliferation and invasion under the same conditions. These effects were apparent under normal serum conditions, but were not as significant. We obtained the mutant allele only (HPIK3CA MT #125) and wild-type allele only (HPIK3CA WT #123) lines from Dr. Bert Vogelstein (Johns Hopkins), to be applied as a comparison for our own studies.

Two glioblastoma stem-like neurosphere lines, without genome wide sequencing data, were produced in our laboratory from patient tumors, 081024 and 081110. These and the D54 glioblastoma line were sequenced for *PIK3CA* mutations using published primer sequences (Table S1) [6]. While 081110 was devoid of mutations in this subunit, 081024 carried 3 mutations on exon 13, each of which has been documented as a SNP (NCBI dbSNP database) [43]. These mutations represented the following amino acid substitutions: V680L, E707K, and L719V. Two of these SNPs (V680L and L719V) were present in D54 cells. None of these substitutions changes the hydropathicity of the residue, and no other mutations were noted.

### *PIK3CA* knockdown decreases Akt activation in GBM cell lines, and knockdown of either *PIK3CA* or *PIK3R1* reduces FAK activity

**Figure 2:**
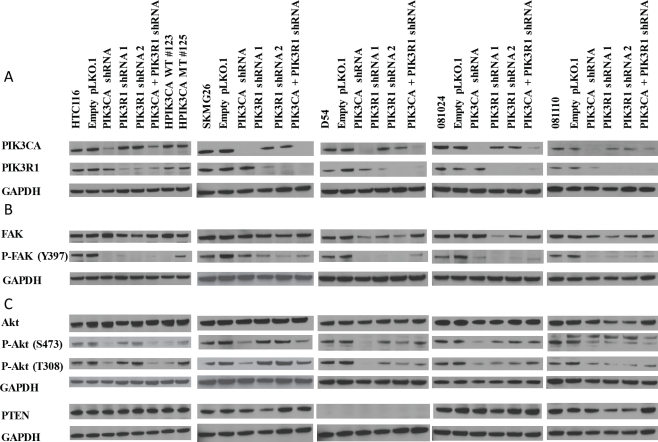
Confirmation of *PIK3CA* and *PIK3R1* knockdown in cell lines A, *PIK3CA* and *PIK3R1* class IA PI3K subunit expression was reduced using lentiviral-mediated shRNA targeted to each subunit specifically, and to each in combination. B, FAK activation at Y397 was observed for each cell line. C, Akt activation at T308 and S473 was observed, as was PTEN status.

To study the effects of *PIK3CA* and *PIK3R1* abrogation in GBM, we significantly reduced protein levels of p110α and p85α using a lentiviral expression vector containing shRNAs targeting each mRNA independently. We knocked-down expression of each subunit separately (and in combination) in the SKMG26, D54, HCT116, 081024, and 081110 cell lines. We produced controls for each line containing only an empty lentiviral vector and no shRNA. Knockdown was confirmed by western blot (Fig. 2A). Although interaction between the *PIK3R1* and *PIK3CA* gene products may be important in the stabilization of the catalytic subunit, we did not observe much decrease in p110α protein levels upon knockdown of p85α.

We examined the phosphorylation levels of Akt at Ser473 and Thr308 in HCT116, D54, and SKMG26 cell lines. Akt activation was significantly reduced at both residues in cells following *PIK3CA* knockdown (Fig.2C). In HCT116 lines, this is similar to the decrease in Akt activity seen in HPIK3CA WT #123 lines, which contain only the wild-type *PIK3CA* allele. *PIK3R1* knockdown did not result in reduced Akt activation, and in the case of SKMG26, this seemed to increase levels of phosphorylation at both of the above residues. In the 081110 neurosphere line, Akt activity was slightly reduced by both *PIK3CA* and *PIK3R1* knockdown. *PIK3R1* and *PIK3CA* knockdown in 081024 neurospheres resulted in a decreased level of Akt phosphorylation at both residues, which was more pronounced in the *PIK3CA* knockdown as compared to the *PIK3R1* knockdown. Neither neurosphere line showed decreased Akt phosphorylation at levels as significant as in the three adherent lines. These results differentiates the 3 adherent lines from the 2 non-adherent neurosphere lines, suggesting that *PIK3CA* knockdown may not affect Akt activity as significantly in neurospheres as compared to adherent lines.

We also studied the activity of FAK (PTK2) in all lines at the Y397 residue (Fig.2B). Both *PIK3R1* and *PIK3CA* knockdown significantly decreased FAK activity in all lines tested.

### Knockdown of *PIK3CA* or *PIK3R1* reduces proliferation in GBM cells in vitro, regardless of *PIK3CA* mutational status

**Figure 3:**
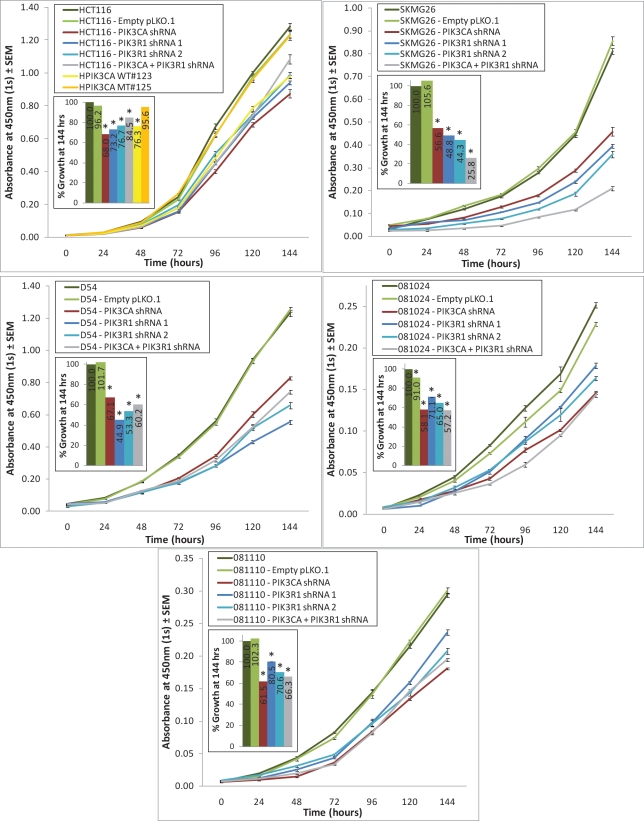
*PIK3CA* or *PIK3R1* knockdown reduces proliferation of various cancer cell lines Cells were plated at 500 or 1000 cells/well and proliferation was measured once per day using the Cell Counting Kit-8 reagent. At 144 hours, all *PIK3CA* and *PIK3R1* knockdown cell lines showed significantly reduced levels of growth as compared to parental and control lines. A more pronounced decrease in proliferation was noted in SKMG26 cells as a result of knockdown of both *PIK3CA* and *PIK3R1*. * represents a p-value ≤ 0.05, and compares a given result to that of the parental line.

**Figure 4:**
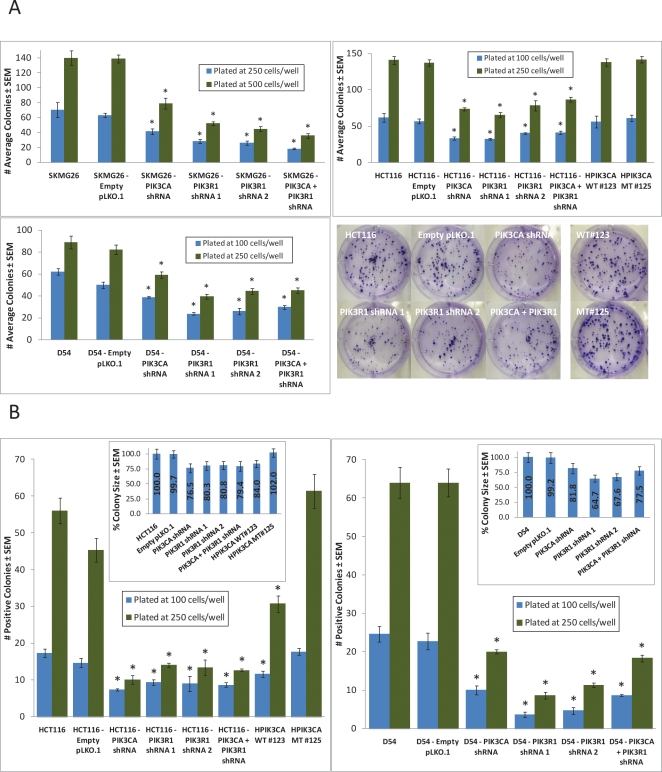
Colony formation is reduced on plastic and soft agar due to either *PIK3CA* or *PIK3R1* knockdown A, Cells were plated on treated plastic 6-well plates at 100, 250, and 500 cells/well. Colonies were stained and counted at ~2 weeks post-plating. In all cells, *PIK3CA* or *PIK3R1* knockdown resulted in a decrease in colony number. B, Cells were plated at 100 and 250 cells/well in top plugs containing appropriate media and 0.4% agarose. At ~2 weeks post-plating, colonies were stained, measured, and counted. A colony was counted as positive if it matched or exceeded the average size of colonies formed by the parental line. In all cell lines, *PIK3CA* or *PIK3R1* knockdown reduced both colony size and number of positive colonies. * represents a p-value ≤ 0.05, and compares a given result to that of the parental line.

Abrogation of either the *PIK3CA* or *PIK3R1* genes significantly reduced proliferation at 144 hours in all 5 cell lines (Fig. 3). Interestingly, in SKMG26 and D54 lines, *PIK3R1* knockdown resulted in a more reduced proliferation rate than did *PIK3CA* knockdown. The combined knockdown of *PIK3CA* and *PIK3R1* only showed a greater effect than either knockdown alone in SKMG26 lines.Similarly, in SKMG26, D54, and HCT116 the colony formation capabilities of the *PIK3CA* and *PIK3R1* knockdown lines were reduced both on treated plastic and, more significantly, in soft agar (Fig. 4). In D54 and SKMG26 lines, both *PIK3CA* and *PIK3R1* knockdown lines showed significantly reduced colony numbers on plastic as compared to parental lines and controls, with the greatest reduction due to *PIK3R1* abrogation (p<0.0001). In HCT116 cells, *PIK3CA* knockdown resulted in a more reduced colony number than *PIK3R1* knockdown, although this was not statistically significant.

In soft agar, a colony was counted as positive if it matched or exceeded the average size of colonies formed by the parental line. SKMG26 cells did not form colonies in soft agar at any agar concentration or cell number. In D54 cells, *PIK3R1* knockdown resulted in a greater reduction in colony size and number of positive colonies than *PIK3CA* knockdown, while *PIK3CA* knockdown showed a slightly greater effect in HCT116 cells. HPIK3CA WT #123 cells also exhibited reduced colony size and number, although this was not as significant as that seen in knockdown lines. HPIK3CA MT #125 cells showed an increase in colony size and number, although this was not statistically significant.

**Figure 5:**
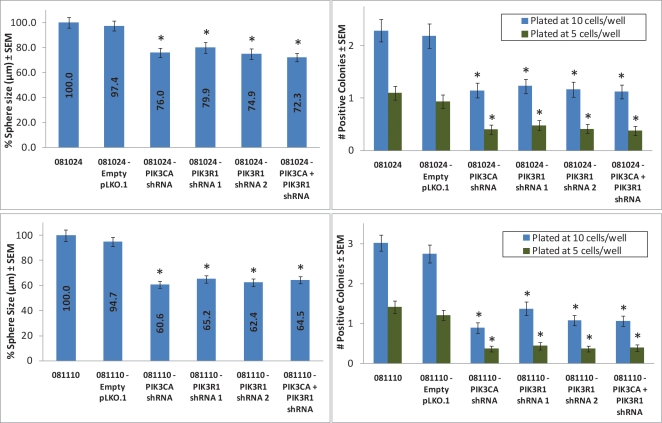
Neurosphere size and number is reduced in *PIK3CA* and *PIK3R1* knockdown cell lines Cells were plated at 1, 5, 10, 100, 250, and 500 cells/well, and at ~2 weeks post-plating, neurospheres were counted and measured. A sphere was considered positive if it matched or exceeded the average size of spheres formed by parental lines. In both 081110 and 081024 cell lines, *PIK3CA* and *PIK3R1* knockdown resulted in reduced neurosphere size and number of positive spheres per well. * represents a p-value ≤ 0.05, and compares a given result to that of the parental line.

081024 and 081110 *PIK3CA* and *PIK3R1* knockdown lines formed significantly smaller colonies over 2 weeks than parental and control lines (Fig. 5). These data suggest that cells lacking *PIK3CA* or *PIK3R1* experience significantly reduced survival under anchorage-independent conditions.

### Knockdown of *PIK3CA* or *PIK3R1* reduces GBM cell invasion and migration on laminin and fibronectin

Fibronectin and laminin are two components of brain extracellular matrix that have been found to be upregulated in glioblastoma, and both may play a role in tumor migration and invasion [17, 19]. Evidence suggests that differential interaction with matrix components may affect the migrational and proliferative capabilities of tumor cell lines. Sengupta et al found that shRNA-mediated inhibition of fibronectin expression delayed GBM tumor progression in mice [21]. Nagato et al showed that knockdown of laminin α4 chain severely limited tumor invasion in vivo [20].

**Figure 6:**
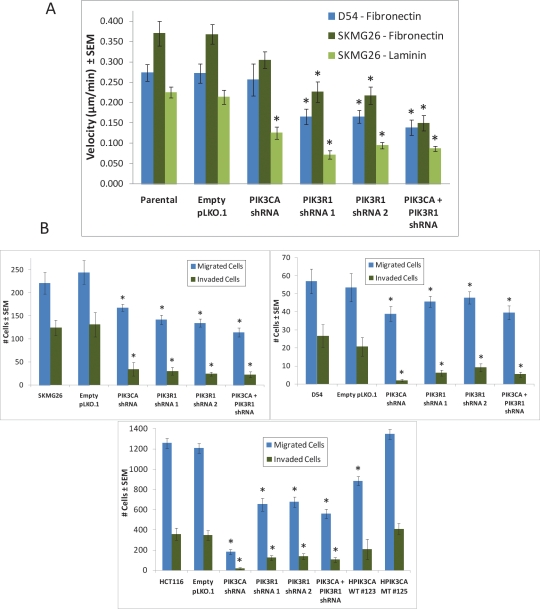
*PIK3CA* knockdown results in reduced cell migration rates on fibronectin and laminin, and both *PIK3CA* and *PIK3R1* knockdown reduce invasion through Matrigel A, Cells were plated on fibronectin or laminin and were imaged every 5 minutes for 24 hours. Average velocity and distance traveled were measured at 6, 8, 12, 16, and 24 hours, and trends were similar for each time point (graph represents the 16 hour time point). In both D54 and SKMG26 cells, *PIK3CA* knockdown did not result in a statistically significant reduced migration rate on fibronectin. *PIK3R1* knockdown reduced migration rates in both lines. D54 lines did not adhere to laminin-coated surfaces. In SKMG26 lines on laminin, both *PIK3CA* and *PIK3R1* knockdown significantly reduced the average rate of cell migration, with the combined knockdown resulting in the greatest decrease in velocity. * represents a p-value ≤ 0.05, and compares a given result to that of the parental line. B, Cells were plated at 2.5x10^5^ or 5x10^5^ cells/well on BD Biocoat Matrigel Invasion Assay inserts. At 24 or 72 hours, migrated (control) and invaded (experimental) cells were stained and counted. Percent invasion is provided for each cell line. Both *PIK3CA* and *PIK3R1* knockdown resulted in a reduced percent invasion as compared to parental lines and controls. * represents a p-value ≤ 0.05, and compares a given result to that of the parental line.

Cells were plated on fibronectin or laminin and were imaged using an Olympus IX81 microsope for 24 hours in order to study the migrational capabilities of the knockdown cell lines. As HCT116 cell lines tend to divide quickly and grow in clumps, migration studies focused on the D54 and SKMG26 lines, which are more motile and therefore trackable. Cells were tracked individually using the Manual Tracking program in ImageJ [44, 45]. 081110 and 081024 lines were not tested in this capacity, as they did not adhere to laminin or fibronectin. On fibronectin, *PIK3CA* knockdown resulted in a reduced average velocity (μm/min) and distance traveled (μm) in both SKMG26 and D54 cell lines as measured at 6, 8, 12, 16, and 24 hours, although not in a statistically significant manner (Fig. 6A, Videos S1-S3). *PIK3R1* knockdown did significantly lower velocity and distance traveled in both cell lines (Videos S4-5). In SKMG26 cells (but not in D54 cells), combined *PIK3CA* and *PIK3R1* knockdown resulted in the greatest decrease in overall velocity (Video S6). D54 cells did not adhere to laminin-coated surfaces. SKMG26 cells on laminin showed significantly reduced migration rates after knockdown of either the *PIK3CA* or *PIK3R1* subunit (Fig. 6A). Migration velocity was decreased more drastically due to *PIK3R1* than *PIK3CA* knockdown, and no combinatorial effect was observed due to combined knockdown. Overall, migration velocity was more greatly reduced on laminin as compared to cells observed on fibronectin. Cell migration rates were reduced in the *PIK3R1* knockdown line as compared to the parental SKMG26 line by 68.0% on laminin versus 38.8% on fibronectin at 16 hours.

We used the BD Biocoat Matrigel Invasion Assay to test the invasive capabilities of SKMG26, D54, and HCT116 cells lacking *PIK3CA* or *PIK3R1* mRNA. For all 3 lines, both *PIK3R1* and *PIK3CA* knockdown significantly reduced the number of invaded cells versus controls, with the lowest percent invasion in *PIK3CA* knockdown lines (Fig. 6B). Together, these data suggest that *PIK3R1* plays an important role in the rate of migration, while *PIK3CA* loss may lead to a greater reduction in invasive capabilities. Both of these characteristics are likely to be important in the infiltration of healthy non-tumor brain tissue by GBM cells.

### MMPs and uPA are reduced as a result of knockdown

**Figure 7:**
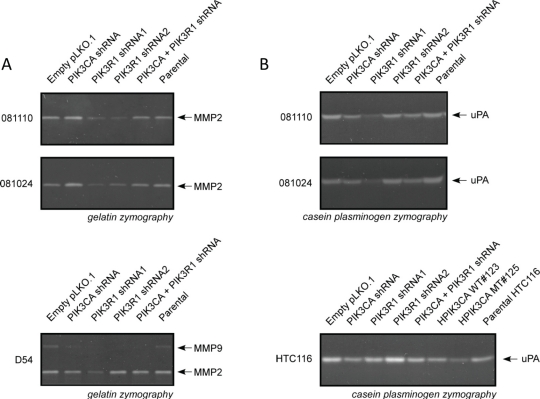
Protease activity is altered in *PIK3R1* knock down cell lines The 48 hour conditioned medium of adherent or suspension cell lines was collected and subjected to gelatin or casein-plasminogen zymography. *PIK3R1* knock down resulted in reduced MMP-2 and uPA activity in neurosphere lines. MMP-9 was reduced in all knock downs in D54 cells. Knock down or mutation of *PIK3CA* resulted in decreased uPA in HTC116 cells.

Gelatin and casein plasminogen zymograms showed that treatment with PIK3R1 shRNA resulted in a decrease in MMP2 and uPA activity in both 081024 and 081110 neurosphere lines (Figure 7). *PIK3CA* knockdown caused no change in activity. In D54 lines, MMP2 activity was similarly reduced by *PIK3R1* knockdown, but not by *PIK3CA* knockdown. D54 cells also express MMP9, which was reduced in all knockdowns as compared to control lines.

## DISCUSSION

Invasion and migration-related pathways were among the most statistically significantly enriched for mutations and expression alterations in our informatics studies using Ingenuity, Partek, and Spotfire. Central to many of these pathways is PI3-Kinase signaling. The validity of our results is strengthened by the similarly significant presence of pathways involved in cell cycle control, apoptosis, and proliferation (Tables S4-S7).

Much research has focused on elucidating the mechanisms of tumor formation and progression caused by individual common mutations, such as PTEN and p53 loss of function, EGFR amplification, or the EGFRvIII variant. However, considering the pathways that are enriched for aberrations may give a more comprehensive view of tumor function. Although we have not addressed epigenetic mechanisms in this study, these should not be excluded from further considerations of the underlying mechanisms for promoting invasion.

Our informatics studies focused primarily on pathways that may affect motility in GBM cells. We found that focal adhesion signaling, integrin signaling, and extracellular component and receptor signaling commonly contained clusters of mutations and/or aberrantly expressed genes (Fig. S1-S3). These signaling pathways are highly interconnected, and most certainly experience altered communication in tumors. While many of the mutations found may be passengers, and thus may not support tumor growth or motility, some, such as mutations found in receptor tyrosine kinases, have already been shown to play a role in cancer [46].

Several integrins are upregulated in GBM, and knockdown of these proteins results in reduced migration and invasion [47, 48]. Our studies confirmed the upregulation of a collection of integrin subunits as compared to normal brain samples by over 5-fold, including αv (8.6-fold), α2 (8.6-fold), α4 (36.9-fold), and β8 (8.3-fold). Several other subunits were upregulated at lower levels, including β3 (3.6-fold). Each of these subunits was expressed in at least half of the tumors studied. The α4 subunit was also found to bear a mutation. Matsunaga et al. showed that knockdown of VLA-4 in acute myelogenous leukemia cells resulted in increased susceptibility of to anoikis- or drug-induced apoptosis [49]. This was likely caused by a reduction in PI3K activity, which is activated by the interaction of the α4 integrin subunit of VLA-4 and fibronectin. Treatment of mice by the same group with an anti-VLA-4 antibody resulted in 100% survival at 62 days.

At least partially dependent on integrin signaling, focal adhesion turnover is likely to be highly important in the migration of tumor cells through healthy brain tissue [12, 14]. The FAK protein itself was neither mutated nor dysregulated according to our studies, although it has been shown to be upregulated in a subset of breast tumors [50]. However, several proteins that may influence the activity of this pathway were upregulated and/or mutated in GBM by our findings. This includes the integrins discussed above, several of which, such as the β3 subunit, activate FAK signaling. Similarly, activated EGFR and PDGFRA, both of which are upregulated (61.7- and 6.8-fold, respectively) and mutated in GBM, can aid in the activation of the FAK pathway [51]. PI3K, PTEN, RAP1B, TLN1, SOS, MAP3K11, TTN, BCAR3, and ROCK1 were each mutated in GBM in our studies, and ROCK1 was found to be upregulated by 8.4-fold as compared to normal brain. NRAS, ARPC1B (subunit 1B of the Arp2/3 complex), PDGFC, PDGFD were upregulated by 13.7-, 5.4-, 16.3-, and 5.5-fold respectively. Interestingly, Fotiadou et al found that mouse embryonic fibroblasts lacking NRAS also lacked phosphorylated FAK, and knockdown of NRAS may be useful in reducing migration in tumors[52].

Extracellular matrix components and their cell receptors play an important role in both integrin signaling and FAK signaling as discussed above. Many structural matrix components are overexpressed or mutated in GBM, and tumor cells may in fact express components that can increase their migratory capabilities [17, 19]. The fibronectin 1 (*FN1*) gene carried a mutation in 1 tumor, and the *COL3A1* gene was considered a potential driver by Parsons et al., with 3 mutations in individual tumors [6]. Several other collagen genes were mutated (*COL14A1, COL23A1, COL4A2, COL4A2, COL4A5, COL6A3*, and *COL6A6*, and *COL3A2* was found to amplified by Rao et al [33]. *FN1*, 2 laminin genes (*LAMC1, LAMA4*), and a collection of collagen genes (*COL1A1, COL20A1, COL5A1, COL4A2, COL1A2, COL12A1, COL22A1, COL6A1, COL28A1, COL6A3*) were upregulated in tumors as compared to normal brain. Several additional collagen genes were overexpressed, but were not expressed in at least half of the tumors studied.

Given that rapid cell proliferation is inherent in GBM and is responsible for the production of the main tumor mass, the roles played by PI3K signaling in proliferation and tumor growth are most commonly studied in GBM. However, our results show that the *PIK3CA* gene, encoding the class IA PI3K catalytic subunit p100α, and the *PIK3R1* gene, encoding the p85α, p55α, and p50α regulatory subunits, may also be key components of the migratory/invasive phenotype in these tumors. Interestingly, while knockdown of either of these subunits reduced proliferation, migration, and invasion in all cell lines tested, the extent of these effects differed slightly in each line. A measurably greater effect resulting from combined *PIK3CA* and *PIK3R1* knockdown, as compared to knockdown of a single gene alone, was observed only in SKMG26 cells. Our studies show that the effects caused by abrogation of *PIK3R1* and *PIK3CA* result from different signaling mechanisms in individual cell lines, likely reflecting different signaling mechanisms in the individual tumors (and perhaps in individual cells of the same tumor). . Although signaling responses to *PIK3CA* and *PIK3R1* knockdown differ among cell lines, proliferation, migration, and invasion were decreased in all lines tested.

p85α activity in the invasive/migratory phenotype has yet to be fully elucidated. p85α may, at least in part, act through focal adhesion signaling, as knockdown results in decreased FAK activation. Cells treated with *PIK3R1*-targeting shRNA show a more reduced average velocity than cells treated with *PIK3CA*-targeting shRNA. However, these cells are still motile on both fibronectin and laminin, and thus the FAK signaling pathway is likely only one component of GBM cell motility. Notably, while both *PIK3CA* and *PIK3R1* knockdown significantly reduces FAK activity in D54 glioblastoma cells, only reduction in *PIK3R1* expression results in statistically significantly slowed cell migration, supporting the only partial role played by FAK signaling in GBM cell motility. The p85α protein product of *PIK3R1* has been shown to interact with several small GTPases, including Cdc42, Rac, and Rho, that through various mechanisms increase cell motility [23, 53]. Park et al found that mir-29 microRNAs upregulate p53 levels, and induce apoptosis, potentially by direct suppression of *PIK3R1* and CDC42 [54]. p85α may also interact with caspase-8 to stimulate motility through Rac activation [55]. Similarly, *PIK3R1* knockdown reduced MMP2 and uPA levels in neurospheres, and reduced MMP2 and MMP9 levels in D54 cells, and reduction of the levels of these proteins has been shown to cause reduced migratory and invasive capacity in glioblastoma cells [56, 57].

There are a variety of small molecule inhibitors available that target p110α, albeit non-specifically, including LY294002 and wortmannin. These and other PI3K inhibitors have been shown to reduce angiogenesis, induce radiosensitivity, and reduce proliferation in GBM cells [58-61]. Although *PIK3R1* has not been studied previously in the context of GBM invasion, given its role in the PI3K pathway, and the 2008 discovery of its mutation in GBM, it is not surprising that *PIK3R1* knockdown shows evidence of decreased invasion in vitro [6]. However, there is currently no inhibitor targeting p85α. Based on the concept that a reduction in migration may sensitize tumor cells to apoptosis, the development of p85α inhibitors used in combination with apoptosis-inducing chemotherapies may be effective in treatment of patients with GBM.

Although much of what has been shown in this study has been implicated previously in other cancers, our findings support the hypotheses that the PI3K signaling pathway has the most significantly mutated genes driving cellular invasion. Within the common mutations that promote GBM formation and growth, those within members of this pathway (PTEN, p110α, p85α and likely receptor tyrosine kinases such as EGFR) are initiating points for signaling enhanced invasion.. Considering the frequency of mutations in the PI3K pathway and its role in growth and invasion, it would be reasonable to increase efforts to develop molecular therapies targeting its pathological signaling in GBM.

## MATERIALS AND METHODS

### Data Collection and Generation of Gene Lists

This study focused on the results of a whole-genome sequencing analysis of Glioblastoma tumors by Parsons et al [6]. We analyzed genes found to be mutated via sequencing, along with ‘target’ genes identified as amplified or deleted by Illumina SNP arrays. We included mutated GBM genes found to be likely drivers of tumor formation and progression by Carter et al [7]. This analysis also included genes amplified or deleted in more than 2% of cases based on a combination of digital karyotyping and a review of published studies by Rao et al [33]. In total, 703 genes were found to be altered in GBM via these data sources.

Since it was likely that many of the low frequency mutations were passengers, a more stringent gene list was also created that included mutations with higher probabilities of being drivers in the formation and invasion of GBM. This list included ‘target’ genes identified as amplified or deleted by Parsons et al [6], CAN genes from the same group that had a passenger probability (low) p-value of 0.05 or better, genes found by Rao et al [33] with a 4% or greater occurrence, and genes determined by Carter et al [7] to be likely drivers via their CHASM program. This stringent list included a total of 59 genes and was analyzed separately from the full list of 703 genes.

### Informatics Analysis of Mutated Gene Lists

Our objective in this analysis was to identify genes, functions, or pathways that are involved in the invasive tumor phenotype and are enriched in GBM. To identify these invasion-related components, we analyzed our lists of GBM-specific mutated, deleted, and/or amplified genes using the Ingenuity Program (Ingenuity Systems) and the Partek Genomics Suite Program (Partek Inc.). The Partek Genomics Suite Gene Ontology function allows for the analysis of gene ontologies (based upon molecular function, cellular component, and biological process as defined by the Gene Ontology Consortium) enriched in GBM tumors. Based on a gene list, Partek assigns statistical significance to each function, component, and process ontology enriched within the list. Statistical significance in this program is defined by an ‘Enrichment Score,’ which is the negative log_e_ transformation of the p-value for the particular ontology. Enrichment Scores above 3 (p ≤ 0.05) are considered statistically significant. The Ingenuity program analyzes genes based upon gene function (as opposed to inclusion in an ontology) and organizes results by p-value into statistically significant canonical pathways and networks. We analyzed the original gene list (703 genes) and the stringent gene list (59 genes) separately using both of these programs.

Additionally, within the identified statistically significant mutation-enriched pathways, the Spotfire DecisionSite program (TIBCO Software Inc.) was utilized to identify up- or down-regulated genes based on expression data from Parsons et al [6]. This gene expression data included 20,619 curated genes in 16 human glioblastoma tumors.

### Cell lines

081110 and 081024 non-adherent neurosphere lines were produced in our laboratory from human tumor samples received immediately following resection from consented GBM patients. Neurospheres were cultured in NeuroCult NS-A basal medium (StemCell Technologies, Vancouver, BC, Canada) supplemented with 10ng/mL FGF (PeproTech, Rocky Hill, NJ), 10ng/mL EGF (PeproTech), and 0.0004% heparin (StemCell Technologies). HCT116 cells and HCT116 lines containing only the wild-type (HPIK3CA WT #123) or mutant (HPIK3CA MT #125) *PIK3CA* alleles were obtained from Dr. Bert Vogelstein (Johns Hopkins), and were grown in McCoy's Modified 5A medium (Sigma-Aldrich) supplemented with 10% FBS. D54 and SKMG26 cells were grown in DMEM (Sigma-Aldrich) supplemented with 10% FBS.

### Lentivirus production

shRNAs targeting the *PIK3CA* and *PIK3R1* mRNAs cloned into the vector pLKO.1 and packaged into *E.coli* were obtained from the Expression Arrest-TRC shRNA Libraries (Broad Institute of MIT and Harvard). The plasmid was amplified on ampicillin-treated plates and colonies were then incubated in 6mL LB Broth (Invitrogen) overnight at 37°C and 300 RPM. The plasmid was purified using the QIAprep Spin Miniprep Kit (Qiagen) as described by the manufacturer. 293T cells in 10cm dishes were treated with Lipofectamine 2000 (Invitrogen), envelope plasmid pMD2.G, packaging plasmid pCMV-R8.74psPAX2, and hairpin-pLKO.1 plasmid. At 18 hours post treatment, the cells were washed and fresh high-BSA media was added. Lentivirus-containing media was collected at 24 and 48 hours and was stored at −80°C until used. Lentivirus was also produced that contained only the empty pLKO.1 plasmid (no shRNA).

HCT116, D54, SKMG26, 081110, and 081024 cell lines were treated with lentivirus targeting either *PIK3CA* or *PIK3R1* plus 8ug/mL polybrene (Sigma-Aldrich). Lines were treated in parallel with a combination of both *PIK3CA* and *PIK3R1* lentiviruses, and with lentivirus containing only the empty pLKO.1 plasmid (no shRNA) as a control. At 24 hours, cells were washed, and shRNA-expressing cells were selected with a concentration of puromycin (Invitrogen) ranging from 0.5-2ug/mL for 72 hours. Cells were then maintained without selection.

### Sequencing

Sequencing was performed as previously described [29]. Briefly, genomic DNA was purified from cell pellets using the DNeasy Blood and Tissue kit (Qiagen). Exons 1, 2, 4, 5, 7, 9, 12, 13, 18, and 20 were amplified using PCR (primers listed in Table S1). PCR products were run on 2% agarose gels, and bands were extracted using the QIAQuick Gel Extraction Kit (Qiagen). Samples were sequenced by GeneWiz (South Plainfield, NJ).

### Growth curves

Cells were treated with 2ug/mL propidium iodide (Sigma-Aldrich) and were sorted by a MoFlo cell sorter (Dako Cytomation) into 96-well plates at 500, 1000 and 2000 cells/well. For 7 days post plating, live cells were counted daily in 10 replicates per line per day using the Cell Counting Kit-8 reagent (Enzo Life Sciences). Absorbance was read at 450 nm for 1 second. Results were normalized to wells containing media only (no cells).

### Colony formation assay

Cell were plated in triplicate at 100, 250, and 500 cells/well in 6-well plates in appropriate medium with 10% FBS. At two weeks post-plating, colonies were stained with crystal violet (Sigma-Aldrich) and counted under a light microscope.

### Colony formation in soft agar

Cells were plated in triplicate at 100 and 250 cells/well in top plugs containing appropriate media plus 0.4% SeaPlaque agarose (Lonza). Bottom plugs contained media plus 0.8% agarose. At approximately two weeks post-plating, colonies were counted and measured under a light microscope.

### Neurosphere formation assay

Cells were treated with 2ug/mL propidium iodide and live cells only were sorted using a MoFlo cell sorter (Cytomation) into 96-well plates at 1, 5, 10, 100, 250, and 500 cells/well. At two weeks post plating, neurospheres were measured for average size and the number of positive neurospheres was recorded at each cell concentration. Neurospheres were considered positive if they matched or exceeded a measurement close to the average size of neurospheres formed by the parental cell line.

### In vitro invasion assay

The BD Biocoat Matrigel Invasion Assay was performed as described by the manufacturer (Becton Dickson). Matrigel inserts were rehydrated with appropriate medium and cells were plated at 2.5x10^5^ or 5x10^5^ cells/well in triplicate in both control and experimental inserts. At 24 or 72 hours, cells remaining in the upper chamber were scraped and invaded cells were stained with crystal violet. Cells were counted using light microscopy on a 1 mm^2^ grid. Two 1 mm^2^ sections were counted at the edge and two at the center of each insert.

### Migration assays on laminin and fibronectin

Cells were plated in triplicate at 10,000 cells/well in 48-well plates coated with laminin or fibronectin (Becton Dickson). At 48-hours post-plating, cells were imaged every 5 minutes for 24 hours at two points per well on an Olympus IX81 microscope (Olympus) with SlideBook 4.2 software (Intelligent Imaging Innovations, Inc.). Cells were maintained in a humidity chamber during imaging at 37°C and 5% CO_2_. Cells were tracked using the Manual Tracking plug-in in ImageJ [44, 45]. Average distance travelled and average velocity were measured for each line at 6, 8, 12, 16, and 24 hours.

### Gelatin zymography and casein plasminogen zymography

Equal amount of protein from cell-conditioned medium was loaded onto a 10% polyacrylamide gel (Sigma-Aldrich) with either 1mg/mL gelatin or 1.5 mg/mL casein and 0.28 units/mL plasminogen for zymography. After SDS-PAGE separation, gels were incubated in renaturing buffer (Invitrogen) to remove SDS and help the renaturation of proteases. The gels were then incubated in developing buffer (Invitrogen) at 37°C for 18 hours. Gels were stained with 0.25% Coomassie Blue R-250 (Thermo Scientific) in 45% methanol, 10% acetic acid, and destained in methanol:water:acetic acid (2.5:6.5:1). No digestion was detected in control casein gels where plasminogen was omitted, confirming the plasminogen activator nature of the uPA band.

### Immunoblotting

Protein levels were measured by immunoblotting using antibodies specific to p110α, p85α, FAK, Phospho-FAK (Y397), Akt, Phospho-Akt (T308), Phospho-Akt (S473) (Cell Signaling Technology, Danvers, MA), PTEN (Abcam, Cambridge, MA), and GAPDH (Santa Cruz Biotechnology). Whole cell lysates transferred onto PVDF membranes were blocked with 5% BSA prior to treatment with antibodies. Following secondary antibody treatment and thorough washing, membranes were treated with the SuperSignal West Pico Chemiluminescent Substrate (Thermo Scientific). Membranes were developed in a dark room using autoradiography film.

### Statistical Analyses

Data were analyzed using the student's t-test. A result with a p-value of less than or equal to 0.05 was considered significant.

## Supplementary Videos

Videos S1-S6. Videos of SKMG26 (Video S1), SKMG26 - Empty pLKO.1 (Video S2), SKMG26 - *PIK3CA* shRNA (Video S3), SKMG26 – *PIK3R1* shRNA 1 (Video S4), SKMG26 – *PIK3R1* shRNA 2 (Video S5), and SKMG26 – *PIK3CA* + *PIK3R1* shRNA (Video S6) cell lines tracked on fibronectin using the Manual Tracking plug-in in ImageJ. Individual tracks are provided for all trackable cells in each video. The videos, supplementary figures and tables can be found online at www.impactjournals.com/oncotarget

## Supplementary Figures, Tables and Videos
































