# Supplementary material for: Abrogation of PIK3CA or PIK3R1 reduces proliferation, migration, and invasion in glioblastoma multiforme cells
Source: Oncotarget. 2011 Nov 5;2(11):833–49. doi: 10.18632/oncotarget.346 (PMC3260001; doi:10.18632/oncotarget.346)
Supplement: Supplementary file 1 [file oncotarget-02-833-s001.pdf]

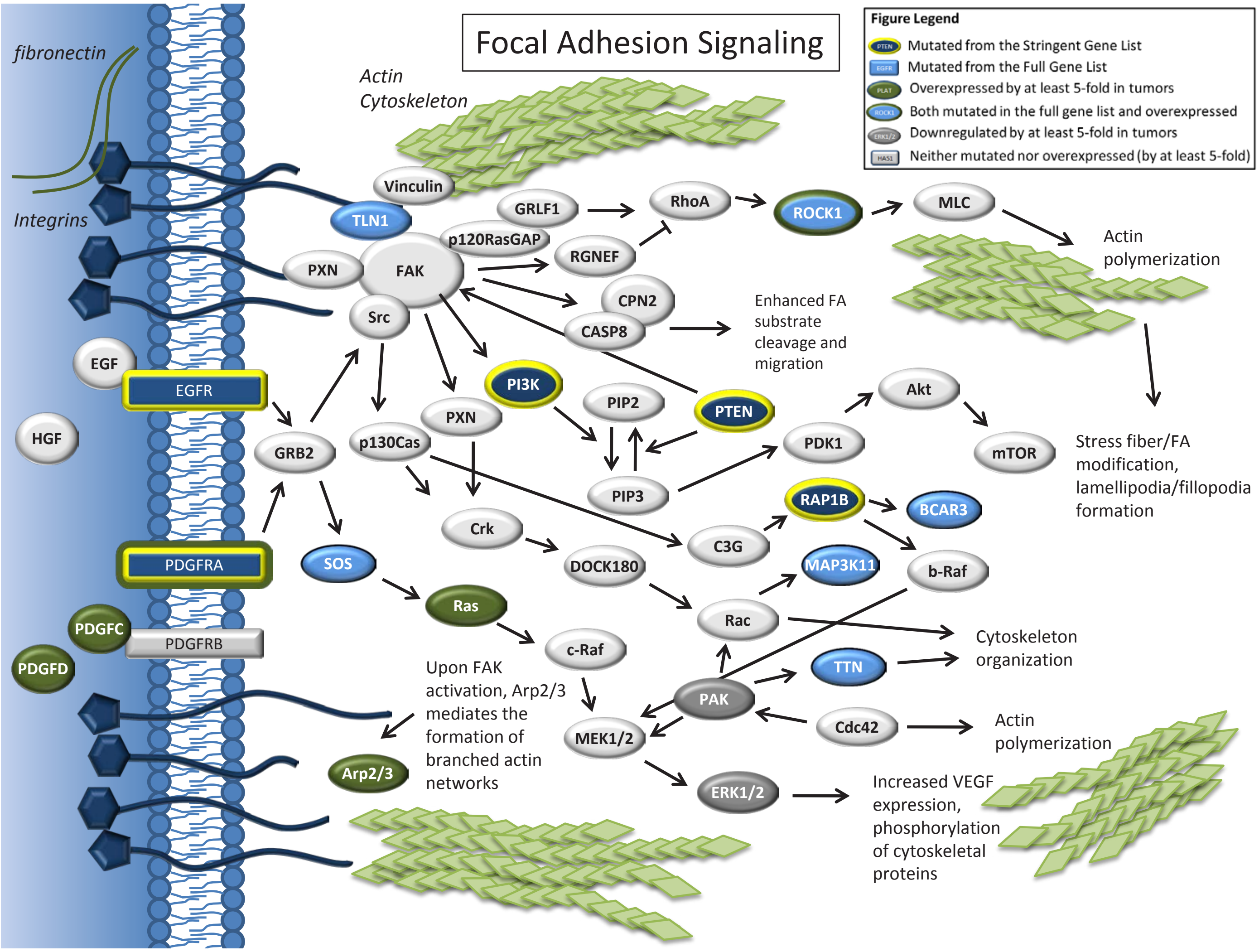

Figure S1: Gene alterations (mutations and expression changes) found in focal adhesion signaling as determined by Ingenuity Pathway Analysis and Partek Genomics Suite. Focal adhesion turnover is likely important for migration of cancer cells, and may lead to invasion of the normal brain in GBM.

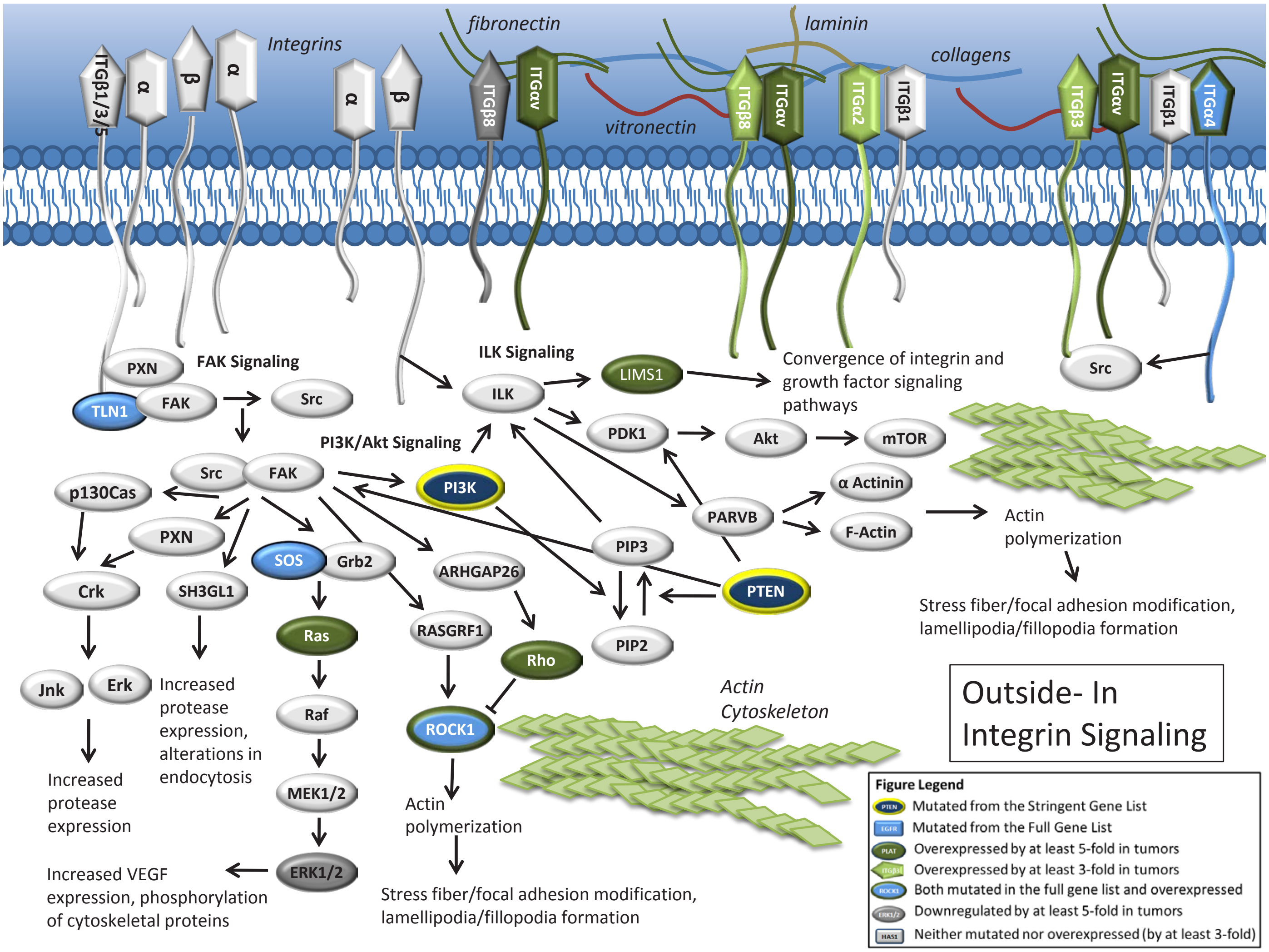

Figure S2: Gene alterations (mutations and expression changes) found in integrin signaling pathways as determined by Ingenuity Pathway Analysis and Partek Genomics Suite. Signaling through integrins activate a combination of pathways involved in motility in invading/migrating GBM cells. Multiple integrin subunits are mutated in GBM, and several matrix components, including laminin, fibronectin, and collagen, are upregulated.

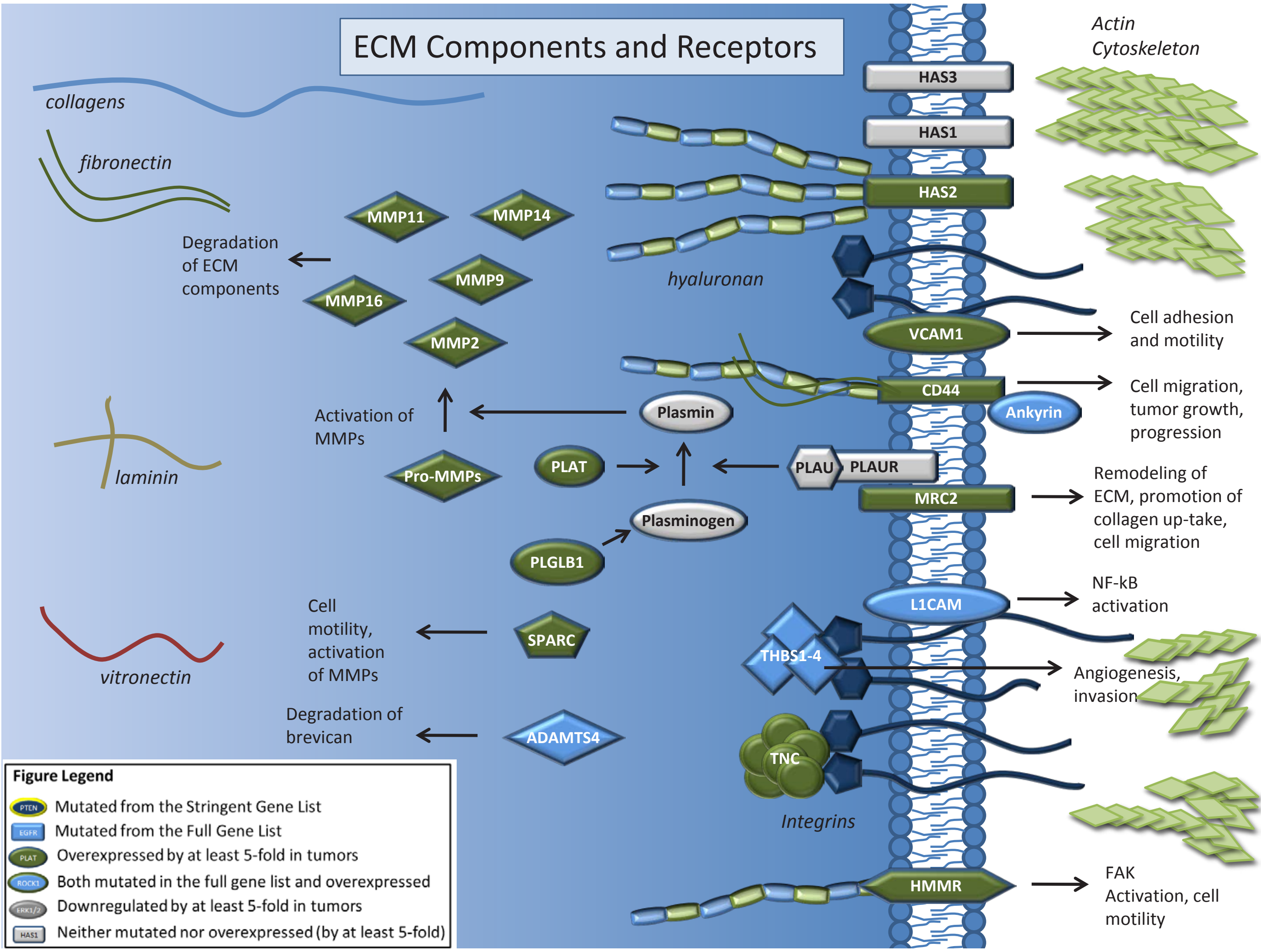

Figure S3: Gene alterations (mutations and expression changes) found in extracellular component signaling pathways as determined by Ingenuity Pathway Analysis and Partek Genomics Suite. The external plasma membrane and extracellular matrix components play integral roles in cell-cell signaling and cell-matrix signaling, and are important for motility and invasion of cancer cells. Signals received by cell receptors due to interactions with the cell environment (membrane components and signaling proteins) activate multiple pathways involved in cancer cell survival and motility, including the PI3K pathway and FAK signaling.
