# Supplementary material for: Abrogation of PIK3CA or PIK3R1 reduces proliferation, migration, and invasion in glioblastoma multiforme cells
Source: Oncotarget. 2011 Nov 5;2(11):833–49. doi: 10.18632/oncotarget.346 (PMC3260001; doi:10.18632/oncotarget.346)
Supplement: Supplementary file 2 [file oncotarget-02-833-s002.docx]

| **Gene - Exon** | **Forward Primer** | **Reverse Primer** | **Sequencing Primer** |
| --- | --- | --- | --- |
| hCT1640694-Ex 1- 1 | GTTTCTGCTTTGGGACAACCAT | CTGCTTCTTGAGTAACACTTACG | GATTCATCTTGAAGAAGTTGATGG |
| hCT1640694-Ex 1- 2 | CTCCACGACCATCATCAGG | GATTACGAAGGTATTGGTTTAGACAG | ACTTGATGCCCCCAAGAATC |
| hCT1640694-Ex 1- 3 | CCCCCTCCATCAACTTCTTC | GGTGTTAAAAATAGTTCCATAGTTCG | CTCAAGAAGCAGAAAGGGAAG |
| hCT1640694-Ex 2- 1 | TCATCAAAAATTTGTTTTAACCTAGC | TATAAGCAGTCCCTGCCTTC | TCTACAGAGTTCCCTGTTTGC |
| hCT1640694-Ex 2- 2 | TTCTGAACGTTTGTAAAGAAGCTG | TATAAGCAGTCCCTGCCTTC | GCTGTGGATCTTAGGGACCTC |
| hCT1640694-Ex 4- 1 | TCTTGTGCTTCAACGTAAATCC | CGGAGATTTGGATGTTCTCC | AAAATAATAAGCATCAGCATTTGAC |
| hCT1640694-Ex 4- 2 | TCTCAACTGCCAATGGACTG | CGGAGATTTGGATGTTCTCC | TTATTCCAGACGCATTTCCAC |
| hCT1640694-Ex5 | TAGTGGATGAAGGCAGCAAC | TTTGTAGAAATGGGGTCTTGC | TTTGAGTCTATCGAGTGTGTGC |
| hCT1640694-Ex7 | GGGGAAAAAGGAAAGAATGG | TGCTGAACCAGTCAAACTCC | TGAATTTTCCTTTTGGGGAAG |
| hCT1640694-Ex9 | GATTGGTTCTTTCCTGTCTCTG | CCACAAATATCAATTTACAACCATTG | TTGCTTTTTCTGTAAATCATCTGTG |
| hCT1640694-Ex12 | TTTATTCTAGATCCATACAACTTCCTTT | AAAGTTGAGAAGCTCATCACTGGTAC | ACCAGTAATATCCACTTTCTTTCTG |
| hCT1640694-Ex13 | CTGAAACTCATGGTGGTTTTG | TGGTTCCAAATCCTAATCTGC | TTTATTGGATTTCAAAAATGAGTG |
| hCT1640694-Ex18 | TCCTTATTCGTTGTCAGTGATTG | GTCAAAACAAATGGCACACG | TGCACCCTGTTTTCTTTTCTC |
| hCT1640694-Ex 20- 1 | TGGGGTAAAGGGAATCAAAAG | CCTATGCAATCGGTCTTTGC | TGACATTTGAGCAAAGACCTG |
| hCT1640694-Ex 20- 2 | TTGCATACATTCGAAAGACC | GGGGATTTTTGTTTTGTTTTG | TTTGTTTTGTTTTGTTTTTT |

**Table S1.** Primers used for sequencing of exons 1, 2, 4, 5, 7, 9, 12, 13, 18, and 20 of the PIK3CA gene.
