# Supplementary material for: Abrogation of PIK3CA or PIK3R1 reduces proliferation, migration, and invasion in glioblastoma multiforme cells
Source: Oncotarget. 2011 Nov 5;2(11):833–49. doi: 10.18632/oncotarget.346 (PMC3260001; doi:10.18632/oncotarget.346)
Supplement: Supplementary file 3 [file oncotarget-02-833-s003.docx]

**Table S2.** Full list of 703 mutated genes analyzed in this study. The mutations column represents the number of mutations found by Parsons, et al. [6], and the deletions and amplifications columns represent those found by both Parsons, et al. [6] and Rao, et al. [33].

| **Gene Name** | **Entrez ID** | **Mutations** | **Deletions** | **Amplifications** |
| --- | --- | --- | --- | --- |
| A2BP1 | 54715 | 0 | 1 | 0 |
| A2M | 2 | 1 | 0 | 0 |
| A4GALT | 53947 | 1 | 0 | 0 |
| ABCA10 | 10349 | 1 | 0 | 0 |
| ABCA4 | 24 | 1 | 0 | 0 |
| ABCA7 | 10347 | 1 | 0 | 0 |
| ABCB6 | 10058 | 1 | 0 | 0 |
| ABCC3 | 8714 | 1 | 0 | 0 |
| ABCC5 | 10057 | 1 | 0 | 0 |
| ABCD2 | 225 | 1 | 0 | 0 |
| ABL2 | 27 | 1 | 0 | 0 |
| ABTB2 | 25841 | 1 | 0 | 0 |
| ACADSB | 36 | 1 | 0 | 0 |
| ACLY | 47 | 1 | 0 | 0 |
| ACSM2B | 348158 | 1 | 0 | 0 |
| ACTRT1 | 139741 | 1 | 0 | 0 |
| ADAM18 | 8749 | 1 | 0 | 0 |
| ADAM29 | 11086 | 1 | 0 | 0 |
| ADAMTS17 | 170691 | 1 | 0 | 0 |
| ADAMTS4 | 9507 | 1 | 0 | 0 |
| ADARB2 | 105 | 1 | 0 | 0 |
| AGFG1 | 3267 | 1 | 0 | 0 |
| AGRN | 375790 | 1 | 0 | 0 |
| AIM1L | 55057 | 1 | 0 | 0 |
| AKAP13 | 11214 | 1 | 0 | 0 |
| ALPI | 248 | 1 | 0 | 0 |
| ALPK3 | 57538 | 1 | 0 | 0 |
| ALX1 | 8092 | 1 | 0 | 0 |
| AMBRA1 | 55626 | 1 | 0 | 0 |
| AMDHD1 | 144193 | 1 | 0 | 0 |
| AMMECR1L | 83607 | 1 | 0 | 0 |
| AMTN | 401138 | 1 | 0 | 0 |
| ANK3 | 288 | 1 | 0 | 0 |
| ANKRD28 | 23243 | 1 | 0 | 0 |
| ANKRD50 | 57182 | 1 | 0 | 0 |
| ANO2 | 57101 | 1 | 0 | 0 |
| ANO3 | 63982 | 1 | 0 | 0 |
| ANO9 | 338440 | 1 | 0 | 0 |
| APOB | 338 | 2 | 0 | 0 |
| APOBEC3H | 164668 | 1 | 0 | 0 |
| ARL1 | 400 | 1 | 0 | 0 |
| ARNT2 | 9915 | 2 | 0 | 0 |
| ARSE | 415 | 1 | 0 | 0 |
| ASH1L | 55870 | 1 | 0 | 0 |
| ASIP | 434 | 1 | 0 | 0 |
| ASTN1 | 460 | 2 | 0 | 0 |
| ATP10B | 23120 | 1 | 0 | 0 |
| ATP12A | 479 | 1 | 0 | 0 |
| ATP2B1 | 490 | 1 | 0 | 0 |
| ATP2C2 | 9914 | 1 | 0 | 0 |
| ATP6V1G3 | 127124 | 1 | 0 | 0 |
| ATP8B1 | 5205 | 1 | 0 | 0 |
| BAI2 | 576 | 1 | 0 | 0 |
| BAMBI | 25805 | 1 | 0 | 0 |
| BCAR3 | 8412 | 1 | 0 | 0 |
| BCL2L2 | 599 | 1 | 0 | 0 |
| BCOR | 54880 | 1 | 0 | 0 |
| BIN1 | 274 | 1 | 0 | 0 |
| BMPER | 168667 | 1 | 0 | 0 |
| BRF1 | 2972 | 1 | 0 | 0 |
| BSDC1 | 55108 | 1 | 0 | 0 |
| C10orf18 | 54906 | 1 | 0 | 0 |
| C10orf47 | 254427 | 1 | 0 | 0 |
| C12orf12 | 196477 | 1 | 0 | 0 |
| C12orf60 | 144608 | 1 | 0 | 0 |
| C14orf115 | 55237 | 1 | 0 | 0 |
| C14orf133 | 63894 | 1 | 0 | 0 |
| C14orf145 | 145508 | 1 | 0 | 0 |
| C15orf42 | 90381 | 1 | 0 | 0 |
| C16orf78 | 123970 | 1 | 0 | 0 |
| C17orf57 | 124989 | 1 | 0 | 0 |
| C18orf25 | 147339 | 1 | 0 | 0 |
| C19orf29 | 58509 | 1 | 0 | 0 |
| C1orf65 | 164127 | 1 | 0 | 0 |
| C20orf78 | 128763 | 1 | 0 | 0 |
| C21orf29 | 54084 | 2 | 0 | 0 |
| C2orf56 | 55471 | 1 | 0 | 0 |
| C2orf63 | 130162 | 1 | 0 | 0 |
| C3orf35 | 339883 | 1 | 0 | 0 |
| C4orf7 | 260436 | 1 | 0 | 0 |
| C5AR1 | 728 | 1 | 0 | 0 |
| C6 | 729 | 1 | 0 | 0 |
| C6orf170 | 221322 | 2 | 0 | 0 |
| C7orf16 | 10842 | 1 | 0 | 0 |
| C8A | 731 | 1 | 0 | 0 |
| C8orf77 | 286103 | 1 | 0 | 0 |
| C8orf80 | 389643 | 1 | 0 | 0 |
| C9orf5 | 23731 | 1 | 0 | 0 |
| CA2 | 760 | 1 | 0 | 0 |
| CACNA1A | 773 | 1 | 0 | 0 |
| CACNA1C | 775 | 1 | 0 | 0 |
| CACNA1H | 8912 | 2 | 0 | 0 |
| CACNA2D3 | 55799 | 1 | 0 | 0 |
| CAMTA1 | 23261 | 0 | 4.6% - Rao, et al. | 0 |
| CARD18 | 59082 | 1 | 0 | 0 |
| CCND2 | 894 | 0 | 0 | 2.2% - Rao, et al. |
| CCNL2 | 81669 | 1 | 0 | 0 |
| CD96 | 10225 | 1 | 0 | 0 |
| CDC2L6 | 23097 | 1 | 0 | 0 |
| CDK4 | 1019 | 0 | 0 | 2 |
| CDK6 | 1021 | 0 | 0 | 2% - Rao, et al. |
| CDKN2A | 1029 | 0 | 10 - Parsons, et al.; 46.4% - Rao, et al. | 0 |
| CDKN2B | 1030 | 0 | 46.4% - Rao, et al. | 0 |
| CDKN2C | 1031 | 0 | 4.6% - Rao, et al. | 0 |
| CDX1 | 1044 | 1 | 0 | 0 |
| CDYL2 | 124359 | 1 | 0 | 0 |
| CELSR3 | 1951 | 1 | 0 | 0 |
| CEP135 | 9662 | 1 | 0 | 0 |
| CFTR | 1080 | 1 | 0 | 0 |
| CHAD | 1101 | 1 | 0 | 0 |
| CHD9 | 80205 | 1 | 0 | 0 |
| CHL1 | 10752 | 1 | 0 | 0 |
| CHRM5 | 1133 | 1 | 0 | 0 |
| CHRNA3 | 1136 | 1 | 0 | 0 |
| CHRNA9 | 55584 | 1 | 0 | 0 |
| CHST13 | 166012 | 1 | 0 | 0 |
| CIZ1 | 25792 | 1 | 0 | 0 |
| CLCN1 | 1180 | 1 | 0 | 0 |
| CLEC16A | 23274 | 1 | 0 | 0 |
| CLEC1A | 51267 | 1 | 0 | 0 |
| CLSPN | 63967 | 1 | 0 | 0 |
| CNR2 | 1269 | 1 | 0 | 0 |
| CNTROB | 116840 | 1 | 0 | 0 |
| COL14A1 | 7373 | 1 | 0 | 0 |
| COL23A1 | 91522 | 1 | 0 | 0 |
| COL3A1 | 1281 | 3 | 0 | 0 |
| COL4A2 | 1284 | 0 | 0 | 2.2% - Rao, et al. |
| COL4A4 | 1286 | 1 | 0 | 0 |
| COL4A5 | 1287 | 1 | 0 | 0 |
| COL6A3 | 1293 | 1 | 0 | 0 |
| COL6A6 | 131873 | 1 | 0 | 0 |
| CPS1 | 1373 | 1 | 0 | 0 |
| CSN3 | 1448 | 1 | 0 | 0 |
| CTNNA2 | 1496 | 1 | 0 | 0 |
| CUBN | 8029 | 1 | 0 | 0 |
| CXCR3 | 2833 | 1 | 0 | 0 |
| CXorf27 | 25763 | 1 | 0 | 0 |
| CXorf59 | 286464 | 1 | 0 | 0 |
| CYP2C19 | 1557 | 1 | 0 | 0 |
| CYP4F12 | 66002 | 1 | 0 | 0 |
| DAB2IP | 153090 | 1 | 0 | 0 |
| DARS2 | 55157 | 1 | 0 | 0 |
| DCAF5 | 8816 | 1 | 0 | 0 |
| DCAF8L1 | 139425 | 1 | 0 | 0 |
| DCTN4 | 51164 | 1 | 0 | 0 |
| DDX54 | 79039 | 1 | 0 | 0 |
| DDX59 | 83479 | 1 | 0 | 0 |
| DDX60 | 55601 | 1 | 0 | 0 |
| DEF8 | 54849 | 1 | 0 | 0 |
| DEFB112 | 245915 | 1 | 0 | 0 |
| DEFB125 | 245938 | 1 | 0 | 0 |
| DEPDC5 | 9681 | 1 | 0 | 0 |
| DIMT1L | 27292 | 1 | 0 | 0 |
| DLGAP2 | 9228 | 1 | 0 | 0 |
| DNAH3 | 55567 | 1 | 0 | 0 |
| DNAH9 | 1770 | 1 | 0 | 0 |
| DOK6 | 220164 | 1 | 0 | 0 |
| DOPEY2 | 9980 | 1 | 0 | 0 |
| DRD3 | 1814 | 1 | 0 | 0 |
| DRG1 | 4733 | 1 | 0 | 0 |
| DSEL | 92126 | 1 | 0 | 0 |
| DSG4 | 147409 | 2 | 0 | 0 |
| DUSP22 | 56940 | 1 | 0 | 0 |
| DUSP3 | 1845 | 1 | 0 | 0 |
| DYNC1H1 | 1778 | 1 | 0 | 0 |
| DYNC1LI2 | 1783 | 1 | 0 | 0 |
| EAF1 | 85403 | 1 | 0 | 0 |
| ECEL1 | 9427 | 1 | 0 | 0 |
| ECHDC2 | 55268 | 1 | 0 | 0 |
| EDC4 | 23644 | 1 | 0 | 0 |
| EEF1A1 | 1915 | 1 | 0 | 0 |
| EFR3A | 23167 | 1 | 0 | 0 |
| EGFR | 1956 | 2 | 0 | 5 |
| EHBP1L1 | 254102 | 1 | 0 | 0 |
| EIF4G1 | 1981 | 1 | 0 | 0 |
| ELFN1 | 392617 | 1 | 0 | 0 |
| EME2 | 197342 | 1 | 0 | 0 |
| EML4 | 27436 | 1 | 0 | 0 |
| EMR4P | 326342 | 1 | 0 | 0 |
| EN2 | 2020 | 1 | 0 | 0 |
| ENPP2 | 5168 | 1 | 0 | 0 |
| EPHA5 | 2044 | 1 | 0 | 0 |
| EPHA6 | 285220 | 1 | 0 | 0 |
| EPO | 2056 | 1 | 0 | 0 |
| ERCC5 | 2073 | 1 | 0 | 0 |
| ESR1 | 2099 | 1 | 0 | 0 |
| ESR2 | 2100 | 1 | 0 | 0 |
| EXOC6B | 23233 | 1 | 0 | 0 |
| F13B | 2165 | 1 | 0 | 0 |
| F2RL1 | 2150 | 1 | 0 | 0 |
| F5 | 2153 | 1 | 0 | 0 |
| FAM134A | 79137 | 1 | 0 | 0 |
| FAM171B | 165215 | 1 | 0 | 0 |
| FAM194B | 220081 | 1 | 0 | 0 |
| FAM83H | 286077 | 1 | 0 | 0 |
| FAM91A1 | 157769 | 1 | 0 | 0 |
| FANCD2 | 2177 | 1 | 0 | 0 |
| FAT1 | 2195 | 1 | 0 | 0 |
| FBN3 | 84467 | 1 | 0 | 0 |
| FBXO40 | 51725 | 1 | 0 | 0 |
| FCGBP | 8857 | 1 | 0 | 0 |
| FCRL5 | 83416 | 1 | 0 | 0 |
| FER1L6 | 654463 | 1 | 0 | 0 |
| FEZ1 | 9638 | 1 | 0 | 0 |
| FFAR1 | 2864 | 1 | 0 | 0 |
| FGD4 | 121512 | 1 | 0 | 0 |
| FGF2 | 2247 | 1 | 0 | 0 |
| FGFR3 | 2261 | 1 | 0 | 0 |
| FIGF | 2277 | 1 | 0 | 0 |
| FLJ13744 | 80078 | 1 | 0 | 0 |
| FLJ41170 | 440200 | 1 | 0 | 0 |
| FLT1 | 2321 | 1 | 0 | 0 |
| FN1 | 2335 | 1 | 0 | 0 |
| FOXB1 | 27023 | 1 | 0 | 0 |
| FOXI1 | 2299 | 1 | 0 | 0 |
| FRMPD1 | 22844 | 1 | 0 | 0 |
| FRMPD4 | 9758 | 2 | 0 | 0 |
| FSCB | 84075 | 1 | 0 | 0 |
| FSTL1 | 11167 | 1 | 0 | 0 |
| FZD10 | 11211 | 1 | 0 | 0 |
| FZD3 | 7976 | 1 | 0 | 0 |
| GABRA6 | 2559 | 1 | 0 | 0 |
| GABRD | 2563 | 1 | 0 | 0 |
| GAD2 | 2572 | 1 | 0 | 0 |
| GAFA3 | 100128236 | 1 | 0 | 0 |
| GAS6 | 2621 | 1 | 0 | 0 |
| GATA4 | 2626 | 1 | 0 | 0 |
| GCM1 | 8521 | 1 | 0 | 0 |
| GCM2 | 9247 | 1 | 0 | 0 |
| GCNT3 | 9245 | 1 | 0 | 0 |
| GHSR | 2693 | 1 | 0 | 0 |
| GIMAP5 | 55340 | 1 | 0 | 0 |
| GK | 2710 | 1 | 0 | 0 |
| GLI1 | 2735 | 0 | 0 | 13.4% - Rao, et al. |
| GLRA1 | 2741 | 1 | 0 | 0 |
| GML | 2765 | 1 | 0 | 0 |
| GNL2 | 29889 | 1 | 0 | 0 |
| GORASP2 | 26003 | 1 | 0 | 0 |
| GP6 | 51206 | 1 | 0 | 0 |
| GPR116 | 221395 | 1 | 0 | 0 |
| GPR132 | 29933 | 1 | 0 | 0 |
| GPR174 | 84636 | 1 | 0 | 0 |
| GPR85 | 54329 | 1 | 0 | 0 |
| GPR98 | 84059 | 3 | 0 | 0 |
| GPRC5C | 55890 | 1 | 0 | 0 |
| GPS1 | 2873 | 1 | 0 | 0 |
| GRAP2 | 9402 | 1 | 0 | 0 |
| GRB10 | 2887 | 0 | 0 | 1 |
| GREB1 | 9687 | 1 | 0 | 0 |
| GRIK4 | 2900 | 1 | 0 | 0 |
| GRIN2B | 2904 | 1 | 0 | 0 |
| GRM1 | 2911 | 1 | 0 | 0 |
| GRM3 | 2913 | 2 | 0 | 0 |
| GTF2H4 | 2968 | 1 | 0 | 0 |
| GUCY1A3 | 2982 | 1 | 0 | 0 |
| GZMH | 2999 | 1 | 0 | 0 |
| HBB | 3043 | 1 | 0 | 0 |
| HCFC2 | 29915 | 1 | 0 | 0 |
| hCG_2011852 | 643677 | 3 | 0 | 0 |
| HDAC2 | 3066 | 1 | 0 | 0 |
| HDAC9 | 9734 | 1 | 0 | 0 |
| HEATR7B2 | 133558 | 1 | 0 | 0 |
| HHIP | 64399 | 1 | 0 | 0 |
| HIVEP1 | 3096 | 1 | 0 | 0 |
| HIVEP2 | 3097 | 1 | 0 | 0 |
| HLA-DRB5 | 3127 | 0 | 1 | 0 |
| HLA-DRB9 | 3132 | 2 | 0 | 0 |
| HMG20A | 10363 | 1 | 0 | 0 |
| HP | 3240 | 1 | 0 | 0 |
| HP1BP3 | 50809 | 1 | 0 | 0 |
| HPCAL4 | 51440 | 1 | 0 | 0 |
| HTR3C | 170572 | 1 | 0 | 0 |
| HTR3E | 285242 | 1 | 0 | 0 |
| IBTK | 25998 | 1 | 0 | 0 |
| IDH1 | 3417 | 4 | 0 | 0 |
| IFLTD1 | 160492 | 1 | 0 | 0 |
| IFNAR1 | 3454 | 1 | 0 | 0 |
| IGSF1 | 3547 | 1 | 0 | 0 |
| IGSF10 | 285313 | 1 | 0 | 0 |
| IL12RB2 | 3595 | 1 | 0 | 0 |
| IL17B | 27190 | 1 | 0 | 0 |
| IL4R | 3566 | 0 | 1 | 0 |
| IMP4 | 92856 | 2 | 0 | 0 |
| INSC | 387755 | 1 | 0 | 0 |
| IPO13 | 9670 | 1 | 0 | 0 |
| IRS1 | 3667 | 1 | 0 | 0 |
| IRX6 | 79190 | 2 | 0 | 0 |
| ITFG3 | 83986 | 1 | 0 | 0 |
| ITGA4 | 3676 | 1 | 0 | 0 |
| ITLN1 | 55600 | 1 | 0 | 0 |
| ITPR3 | 3710 | 1 | 0 | 0 |
| IVNS1ABP | 10625 | 1 | 0 | 0 |
| KCNA4 | 3739 | 1 | 0 | 0 |
| KCNB2 | 9312 | 1 | 0 | 0 |
| KCND2 | 3751 | 1 | 0 | 0 |
| KCNG3 | 170850 | 1 | 0 | 0 |
| KCNH1 | 3756 | 1 | 0 | 0 |
| KCNH5 | 27133 | 1 | 0 | 0 |
| KCNJ15 | 3772 | 1 | 0 | 0 |
| KCNK1 | 3775 | 1 | 0 | 0 |
| KCP | 375616 | 1 | 0 | 0 |
| KDR | 3791 | 1 | 0 | 0 |
| KIAA0226 | 9711 | 1 | 0 | 0 |
| KIAA0649 | 9858 | 1 | 0 | 0 |
| KIAA0652 | 9776 | 1 | 0 | 0 |
| KIAA0831 | 22863 | 1 | 0 | 0 |
| KIAA1377 | 57562 | 1 | 0 | 0 |
| KIAA1467 | 57613 | 1 | 0 | 0 |
| KIAA1486 | 57624 | 1 | 0 | 0 |
| KIAA1524 | 57650 | 1 | 0 | 0 |
| KIAA1804 | 84451 | 2 | 0 | 0 |
| KIAA1967 | 57805 | 1 | 0 | 0 |
| KIAA2022 | 340533 | 1 | 0 | 0 |
| KIAA2026 | 158358 | 1 | 0 | 0 |
| KLHL10 | 317719 | 1 | 0 | 0 |
| KLK9 | 284366 | 1 | 0 | 0 |
| KLRG1 | 10219 | 1 | 0 | 0 |
| KNTC1 | 9735 | 1 | 0 | 0 |
| KRT222 | 125113 | 1 | 0 | 0 |
| KRT34 | 3885 | 1 | 0 | 0 |
| L1CAM | 3897 | 1 | 0 | 0 |
| LACE1 | 246269 | 1 | 0 | 0 |
| LAMA1 | 284217 | 1 | 0 | 0 |
| LAMA3 | 3909 | 1 | 0 | 0 |
| LAMB3 | 3914 | 1 | 0 | 0 |
| LAMP3 | 27074 | 1 | 0 | 0 |
| LARP4B | 23185 | 1 | 0 | 0 |
| LBP | 3929 | 1 | 0 | 0 |
| LCT | 3938 | 1 | 0 | 0 |
| LENG8 | 114823 | 1 | 0 | 0 |
| LETMD1 | 25875 | 1 | 0 | 0 |
| LILRA4 | 23547 | 1 | 0 | 0 |
| LMX1A | 4009 | 2 | 0 | 0 |
| LOC284912 | 284912 | 1 | 0 | 0 |
| LOC340096 | 340096 | 1 | 0 | 0 |
| LOC441233 | 441233 | 1 | 0 | 0 |
| LONRF3 | 79836 | 1 | 0 | 0 |
| LPAL2 | 80350 | 1 | 0 | 0 |
| LPHN3 | 23284 | 1 | 0 | 0 |
| LPL | 4023 | 1 | 0 | 0 |
| LRAT | 9227 | 1 | 0 | 0 |
| LRFN5 | 145581 | 1 | 0 | 0 |
| LRP1B | 53353 | 0 | 2 | 0 |
| LRP2 | 4036 | 3 | 0 | 0 |
| LRRC33 | 375387 | 1 | 0 | 0 |
| LRRC4B | 94030 | 1 | 0 | 0 |
| LRRC7 | 57554 | 2 | 0 | 0 |
| LRRIQ1 | 84125 | 1 | 0 | 0 |
| LRRK1 | 79705 | 2 | 0 | 0 |
| LRRN3 | 54674 | 1 | 0 | 0 |
| LTB4R2 | 56413 | 1 | 0 | 0 |
| LTBP4 | 8425 | 1 | 0 | 0 |
| LTK | 4058 | 1 | 0 | 0 |
| LVRN | 206338 | 1 | 0 | 0 |
| LYNX1 | 66004 | 1 | 0 | 0 |
| LYPLA1 | 10434 | 1 | 0 | 0 |
| MACF1 | 23499 | 1 | 0 | 0 |
| MAGEB10 | 139422 | 1 | 0 | 0 |
| MAGEB18 | 286514 | 1 | 0 | 0 |
| MAGEC1 | 9947 | 1 | 0 | 0 |
| MAN2B1 | 4125 | 1 | 0 | 0 |
| MAP1S | 55201 | 1 | 0 | 0 |
| MAP3K11 | 4296 | 1 | 0 | 0 |
| MAP3K9 | 4293 | 1 | 0 | 0 |
| MARK3 | 4140 | 1 | 0 | 0 |
| MBD1 | 4152 | 1 | 0 | 0 |
| MCPH1 | 79648 | 1 | 0 | 0 |
| MDM2 | 4193 | 0 | 0 | 9.2% - Rao, et al. |
| MDM4 | 4194 | 0 | 0 | 1- Parsons, et al.; 7.7% - Rao et al. |
| MEFV | 4210 | 1 | 0 | 0 |
| MEGF6 | 1953 | 0 | 1 | 0 |
| MET | 4233 | 0 | 0 | 3.7% - Rao, et al. |
| MGAM | 8972 | 1 | 0 | 0 |
| MME | 4311 | 1 | 0 | 0 |
| MON2 | 23041 | 1 | 0 | 0 |
| MPDU1 | 9526 | 1 | 0 | 0 |
| MPZ | 4359 | 1 | 0 | 0 |
| MRPL16 | 54948 | 1 | 0 | 0 |
| MRPL37 | 51253 | 1 | 0 | 0 |
| MRPL55 | 128308 | 1 | 0 | 0 |
| MSL2 | 55167 | 1 | 0 | 0 |
| MSRB3 | 253827 | 1 | 0 | 0 |
| MTHFD2L | 441024 | 1 | 0 | 0 |
| MTUS2 | 23281 | 2 | 0 | 0 |
| MTX2 | 10651 | 1 | 0 | 0 |
| MUC16 | 94025 | 1 | 0 | 0 |
| MUC7 | 4589 | 1 | 0 | 0 |
| MXRA5 | 25878 | 1 | 0 | 0 |
| MYBPHL | 343263 | 1 | 0 | 0 |
| MYC | 4609 | 0 | 0 | 2% - Rao, et al. |
| MYCN | 4613 | 0 | 0 | 1 |
| MYH14 | 79784 | 1 | 0 | 0 |
| MYH15 | 22989 | 1 | 0 | 0 |
| MYO16 | 23026 | 1 | 0 | 0 |
| MYO1B | 4430 | 2 | 0 | 0 |
| MYO1D | 4642 | 1 | 0 | 0 |
| MYO3A | 53904 | 1 | 0 | 0 |
| MYO3B | 140469 | 1 | 0 | 0 |
| MYO5C | 55930 | 1 | 0 | 0 |
| MYRIP | 25924 | 1 | 0 | 0 |
| NAPSB | 256236 | 1 | 0 | 0 |
| NAT14 | 57106 | 1 | 0 | 0 |
| NCRNA00169 | 400508 | 1 | 0 | 0 |
| NDUFB2 | 4708 | 1 | 0 | 0 |
| NDUFS6 | 4726 | 1 | 0 | 0 |
| NEIL3 | 55247 | 1 | 0 | 0 |
| NF1 | 4763 | 4 | 2.2% - Rao, et al. | 0 |
| NGEF | 25791 | 2 | 0 | 0 |
| NHLRC4 | 283948 | 1 | 0 | 0 |
| NHS | 4810 | 1 | 0 | 0 |
| NISCH | 11188 | 1 | 0 | 0 |
| NKRF | 55922 | 1 | 0 | 0 |
| NLGN2 | 57555 | 1 | 0 | 0 |
| NLRP7 | 199713 | 1 | 0 | 0 |
| NMUR1 | 10316 | 1 | 0 | 0 |
| NOTCH1 | 4851 | 1 | 0 | 0 |
| NPC1L1 | 29881 | 1 | 0 | 0 |
| NPFFR2 | 10886 | 1 | 0 | 0 |
| NRAP | 4892 | 1 | 0 | 0 |
| NRK | 203447 | 1 | 0 | 0 |
| NRN1L | 123904 | 1 | 0 | 0 |
| NUP160 | 23279 | 1 | 0 | 0 |
| NXF5 | 55998 | 1 | 0 | 0 |
| NXPH1 | 30010 | 1 | 0 | 0 |
| OAS3 | 4940 | 1 | 0 | 0 |
| ODZ2 | 57451 | 1 | 0 | 0 |
| OPRD1 | 4985 | 1 | 0 | 0 |
| OR10T2 | 128360 | 1 | 0 | 0 |
| OR13J1 | 392309 | 1 | 0 | 0 |
| OR1L8 | 138881 | 1 | 0 | 0 |
| OR1P1 | 8391 | 1 | 0 | 0 |
| OR2A12 | 346525 | 1 | 0 | 0 |
| OR2A3P | 202861 | 1 | 0 | 0 |
| OR2AG1 | 144125 | 1 | 0 | 0 |
| OR2D2 | 120776 | 1 | 0 | 0 |
| OR2G3 | 81469 | 1 | 0 | 0 |
| OR2L13 | 284521 | 2 | 0 | 0 |
| OR2L2 | 26246 | 1 | 0 | 0 |
| OR2T4 | 127074 | 1 | 0 | 0 |
| OR4P4 | 81300 | 1 | 0 | 0 |
| OR52A5 | 390054 | 1 | 0 | 0 |
| OR52B2 | 255725 | 1 | 0 | 0 |
| OR52N4 | 390072 | 1 | 0 | 0 |
| OR5M8 | 219484 | 1 | 0 | 0 |
| OR6C74 | 254783 | 1 | 0 | 0 |
| OR8D2 | 283160 | 1 | 0 | 0 |
| OSBPL5 | 114879 | 1 | 0 | 0 |
| OSR2 | 116039 | 1 | 0 | 0 |
| OVCH1 | 341350 | 1 | 0 | 0 |
| OVOL2 | 58495 | 1 | 0 | 0 |
| PAPOLG | 64895 | 1 | 0 | 0 |
| PAPPA2 | 60676 | 1 | 0 | 0 |
| PARK2 | 5071 | 0 | 3.8% - Rao, et al. | 0 |
| PARP11 | 57097 | 1 | 0 | 0 |
| PAX9 | 5083 | 1 | 0 | 0 |
| PCDH11X | 27328 | 1 | 0 | 0 |
| PCDHA10 | 56139 | 1 | 0 | 0 |
| PCDHA13 | 56136 | 1 | 0 | 0 |
| PCGF2 | 7703 | 1 | 0 | 0 |
| PCLO | 27445 | 1 | 0 | 0 |
| PDCD11 | 22984 | 1 | 0 | 0 |
| PDE1C | 5137 | 1 | 0 | 0 |
| PDGFRA | 5156 | 1 | 0 | 7.7% - Rao, et al. |
| PDHA2 | 5161 | 1 | 0 | 0 |
| PDIA2 | 64714 | 1 | 0 | 0 |
| PDZD2 | 23037 | 1 | 0 | 0 |
| PEAR1 | 375033 | 1 | 0 | 0 |
| PEX1 | 5189 | 1 | 0 | 0 |
| PHF13 | 148479 | 0 | 0 | 1 |
| PHF2 | 5253 | 1 | 0 | 0 |
| PHIP | 55023 | 2 | 0 | 0 |
| PHLPP2 | 23035 | 1 | 0 | 0 |
| PIGG | 54872 | 1 | 0 | 0 |
| PIGR | 5284 | 1 | 0 | 0 |
| PIK3C2B | 5287 | 0 | 0 | 7.7% - Rao, et al. |
| PIK3C2G | 5288 | 1 | 0 | 0 |
| PIK3CA | 5290 | 2 | 0 | 0 |
| PIK3R1 | 5295 | 2 | 0 | 0 |
| PIK3R5 | 23533 | 1 | 0 | 0 |
| PIKFYVE | 200576 | 1 | 0 | 0 |
| PITPNM1 | 9600 | 1 | 0 | 0 |
| PITPNM3 | 83394 | 1 | 0 | 0 |
| PKD1L2 | 114780 | 1 | 0 | 0 |
| PKHD1 | 5314 | 3 | 0 | 0 |
| PLEKHA4 | 57664 | 1 | 0 | 0 |
| PLEKHG4B | 153478 | 1 | 0 | 0 |
| PLXNA3 | 55558 | 1 | 0 | 0 |
| PLXNB2 | 23654 | 1 | 0 | 0 |
| PMS1 | 5378 | 1 | 0 | 0 |
| PNLIP | 5406 | 1 | 0 | 0 |
| POLE | 5426 | 1 | 0 | 0 |
| POLM | 27434 | 1 | 0 | 0 |
| POLR3B | 55703 | 1 | 0 | 0 |
| POU6F2 | 11281 | 1 | 0 | 0 |
| PPP1R12C | 54776 | 1 | 0 | 0 |
| PRDM16 | 63976 | 1 | 0 | 0 |
| PRIC285 | 85441 | 1 | 0 | 0 |
| PRKDC | 5591 | 1 | 0 | 0 |
| PRKG2 | 5593 | 1 | 0 | 0 |
| PRMT10 | 90826 | 1 | 0 | 0 |
| PRR12 | 57479 | 1 | 0 | 0 |
| PRSS22 | 64063 | 1 | 0 | 0 |
| PSMD6 | 9861 | 1 | 0 | 0 |
| PSMD8 | 5714 | 1 | 0 | 0 |
| PTAR1 | 375743 | 1 | 0 | 0 |
| PTEN | 5728 | 5 | 1 - Parsons, et al.; 10.9% - Rao, et al. | 0 |
| PTGFR | 5737 | 1 | 0 | 0 |
| PTK2B | 2185 | 1 | 0 | 0 |
| PTPN23 | 25930 | 1 | 0 | 0 |
| PTPRM | 5797 | 1 | 0 | 0 |
| RAB3C | 115827 | 1 | 0 | 0 |
| RAC2 | 5880 | 1 | 0 | 0 |
| RAD51L3 | 5892 | 1 | 0 | 0 |
| RAD52 | 5893 | 1 | 0 | 0 |
| RALBP1 | 10928 | 1 | 0 | 0 |
| RAP1B | 5908 | 0 | 0 | 1 |
| RAPGEF6 | 51735 | 1 | 0 | 0 |
| RAPGEFL1 | 51195 | 1 | 0 | 0 |
| RASGRF2 | 5924 | 1 | 0 | 0 |
| RB1 | 5925 | 2 | 1 - Parsons, et al.; 6.3% - Rao, et al. | 0 |
| RBM25 | 58517 | 1 | 0 | 0 |
| RBM27 | 54439 | 2 | 0 | 0 |
| RBM39 | 9584 | 1 | 0 | 0 |
| RBMS3 | 27303 | 1 | 0 | 0 |
| RBP3 | 5949 | 1 | 0 | 0 |
| REG1B | 5968 | 1 | 0 | 0 |
| REN | 5972 | 1 | 0 | 0 |
| RET | 5979 | 1 | 0 | 0 |
| RFC2 | 5982 | 1 | 0 | 0 |
| RGSL1 | 353299 | 1 | 0 | 0 |
| RHOT1 | 55288 | 1 | 0 | 0 |
| RICH2 | 9912 | 1 | 0 | 0 |
| RIMBP2 | 23504 | 1 | 0 | 0 |
| RIPK4 | 54101 | 1 | 0 | 0 |
| RLTPR | 146206 | 1 | 0 | 0 |
| ROCK1 | 6093 | 1 | 0 | 0 |
| RP1L1 | 94137 | 1 | 0 | 0 |
| RPL11 | 6135 | 1 | 0 | 0 |
| RPS2P32 | 256355 | 1 | 0 | 0 |
| RPUSD3 | 285367 | 1 | 0 | 0 |
| RRP12 | 23223 | 1 | 0 | 0 |
| RYR2 | 6262 | 2 | 0 | 0 |
| RYR3 | 6263 | 1 | 0 | 0 |
| SAMD11 | 148398 | 1 | 0 | 0 |
| SAMD9 | 54809 | 1 | 0 | 0 |
| SCAI | 286205 | 1 | 0 | 0 |
| SCN1B | 6324 | 1 | 0 | 0 |
| SCN3A | 6328 | 1 | 0 | 0 |
| SCN3B | 55800 | 1 | 0 | 0 |
| SCN5A | 6331 | 1 | 0 | 0 |
| SCN9A | 6335 | 2 | 0 | 0 |
| SCUBE1 | 80274 | 1 | 0 | 0 |
| SDC3 | 9672 | 1 | 0 | 0 |
| SDR9C7 | 121214 | 1 | 0 | 0 |
| SEC24C | 9632 | 1 | 0 | 0 |
| SERPINA12 | 145264 | 2 | 0 | 0 |
| SERPINB7 | 8710 | 1 | 0 | 0 |
| SERPING1 | 710 | 1 | 0 | 0 |
| SEZ6 | 124925 | 1 | 0 | 0 |
| SEZ6L | 23544 | 1 | 0 | 0 |
| SFTPB | 6439 | 1 | 0 | 0 |
| SGK2 | 10110 | 1 | 0 | 0 |
| SGPP2 | 130367 | 1 | 0 | 0 |
| SH2D6 | 284948 | 1 | 0 | 0 |
| SHMT2 | 6472 | 1 | 0 | 0 |
| SIGLEC5 | 8778 | 1 | 0 | 0 |
| SKP2 | 6502 | 2 | 0 | 0 |
| SLC11A1 | 6556 | 1 | 0 | 0 |
| SLC12A5 | 57468 | 1 | 0 | 0 |
| SLC14A1 | 6563 | 1 | 0 | 0 |
| SLC14A2 | 8170 | 1 | 0 | 0 |
| SLC16A5 | 9121 | 1 | 0 | 0 |
| SLC1A2 | 6506 | 1 | 0 | 0 |
| SLC22A3 | 6581 | 1 | 0 | 0 |
| SLC30A9 | 10463 | 1 | 0 | 0 |
| SLC35F2 | 54733 | 1 | 0 | 0 |
| SLC46A3 | 283537 | 1 | 0 | 0 |
| SLC4A1 | 6521 | 1 | 0 | 0 |
| SLC5A7 | 60482 | 1 | 0 | 0 |
| SLC7A13 | 157724 | 1 | 0 | 0 |
| SLC7A6 | 9057 | 1 | 0 | 0 |
| SLC8A1 | 6546 | 1 | 0 | 0 |
| SLC9A1 | 6548 | 1 | 0 | 0 |
| SLC9A2 | 6549 | 1 | 0 | 0 |
| SLC9A4 | 389015 | 1 | 0 | 0 |
| SLCO6A1 | 133482 | 1 | 0 | 0 |
| SLITRK5 | 26050 | 1 | 0 | 0 |
| SMARCA2 | 6595 | 1 | 0 | 0 |
| SMARCC2 | 6601 | 1 | 0 | 0 |
| SMC3 | 9126 | 1 | 0 | 0 |
| SMCR8 | 140775 | 1 | 0 | 0 |
| SMG7 | 9887 | 1 | 0 | 0 |
| SNRPA | 6626 | 1 | 0 | 0 |
| SOHLH1 | 402381 | 1 | 0 | 0 |
| SOS1 | 6654 | 1 | 0 | 0 |
| SP100 | 6672 | 1 | 0 | 0 |
| SPEF2 | 79925 | 1 | 0 | 0 |
| SPIN3 | 169981 | 1 | 0 | 0 |
| SPRED2 | 200734 | 1 | 0 | 0 |
| SPRY2 | 10253 | 0 | 6.3% - Rao, et al. | 0 |
| SPTBN4 | 57731 | 1 | 0 | 0 |
| SPTBN5 | 51332 | 1 | 0 | 0 |
| SRGAP1 | 57522 | 1 | 0 | 0 |
| ST14 | 6768 | 1 | 0 | 0 |
| ST8SIA4 | 7903 | 1 | 0 | 0 |
| STAB1 | 23166 | 1 | 0 | 0 |
| STAP2 | 55620 | 1 | 0 | 0 |
| STEAP3 | 55240 | 1 | 0 | 0 |
| STIM2 | 57620 | 1 | 0 | 0 |
| STK39 | 27347 | 1 | 0 | 0 |
| STRA6 | 64220 | 1 | 0 | 0 |
| STXBP2 | 6813 | 1 | 0 | 0 |
| SULT6B1 | 391365 | 1 | 0 | 0 |
| SV2B | 9899 | 1 | 0 | 0 |
| SYNE1 | 23345 | 1 | 0 | 0 |
| SYNM | 23336 | 1 | 0 | 0 |
| TACC2 | 10579 | 1 | 0 | 0 |
| TACC3 | 10460 | 1 | 0 | 0 |
| TAF4B | 6875 | 1 | 0 | 0 |
| TAOK1 | 57551 | 1 | 0 | 0 |
| TBC1D8B | 54885 | 1 | 0 | 0 |
| TBX20 | 57057 | 1 | 0 | 0 |
| TBX22 | 50945 | 1 | 0 | 0 |
| TDRD5 | 163589 | 1 | 0 | 0 |
| TDRD9 | 122402 | 1 | 0 | 0 |
| TEPP | 374739 | 1 | 0 | 0 |
| TGM1 | 7051 | 1 | 0 | 0 |
| TGM5 | 9333 | 1 | 0 | 0 |
| THAP9 | 79725 | 1 | 0 | 0 |
| THBS1 | 7057 | 1 | 0 | 0 |
| THOC6 | 79228 | 1 | 0 | 0 |
| THRAP3 | 9967 | 1 | 0 | 0 |
| TLL1 | 7092 | 1 | 0 | 0 |
| TLN1 | 7094 | 1 | 0 | 0 |
| TLX3 | 30012 | 1 | 0 | 0 |
| TM9SF4 | 9777 | 1 | 0 | 0 |
| TMCO5A | 145942 | 1 | 0 | 0 |
| TMEM130 | 222865 | 1 | 0 | 0 |
| TMEM131 | 23505 | 1 | 0 | 0 |
| TMEM132B | 114795 | 0 | 1 | 0 |
| TMEM132C | 92293 | 1 | 0 | 0 |
| TMEM146 | 257062 | 1 | 0 | 0 |
| TMEM168 | 64418 | 1 | 0 | 0 |
| TMPRSS4 | 56649 | 1 | 0 | 0 |
| TNFSF13B | 10673 | 0 | 0 | 2.2% - Rao, et al. |
| TNFSF18 | 8995 | 1 | 0 | 0 |
| TNIP2 | 79155 | 1 | 0 | 0 |
| TNMD | 64102 | 1 | 0 | 0 |
| TNRC4 | 11189 | 1 | 0 | 0 |
| TNS4 | 84951 | 1 | 0 | 0 |
| TOR1A | 1861 | 1 | 0 | 0 |
| TP53 | 7157 | 12 | 1 - Parsons, et al.; 1.9% - Rao, et al. | 0 |
| TRAPPC3 | 27095 | 1 | 0 | 0 |
| TREML2 | 79865 | 1 | 0 | 0 |
| TRIM42 | 287015 | 1 | 0 | 0 |
| TRIM45 | 80263 | 1 | 0 | 0 |
| TRIM55 | 84675 | 1 | 0 | 0 |
| TRIM58 | 25893 | 1 | 0 | 0 |
| TRIML2 | 205860 | 1 | 0 | 0 |
| TRIO | 7204 | 1 | 0 | 0 |
| TRPM3 | 80036 | 1 | 0 | 0 |
| TRPM5 | 29850 | 1 | 0 | 0 |
| TRPV5 | 56302 | 2 | 0 | 0 |
| TSNARE1 | 203062 | 0 | 1 | 0 |
| TSPAN32 | 10077 | 1 | 0 | 0 |
| TTC27 | 55622 | 1 | 0 | 0 |
| TTC35 | 9694 | 1 | 0 | 0 |
| TTC6 | 115669 | 1 | 0 | 0 |
| TTLL5 | 23093 | 1 | 0 | 0 |
| TTN | 7273 | 2 | 0 | 0 |
| TUBGCP3 | 10426 | 1 | 0 | 0 |
| TXNDC3 | 51314 | 1 | 0 | 0 |
| UBR5 | 51366 | 1 | 0 | 0 |
| UGDH | 7358 | 1 | 0 | 0 |
| UGT1A6 | 54578 | 1 | 0 | 0 |
| UPK3B | 80761 | 1 | 0 | 0 |
| URB2 | 9816 | 2 | 0 | 0 |
| USH2A | 7399 | 1 | 0 | 0 |
| VGLL2 | 245806 | 1 | 0 | 0 |
| VPS11 | 55823 | 1 | 0 | 0 |
| VPS13B | 157680 | 1 | 0 | 0 |
| VWA2 | 340706 | 1 | 0 | 0 |
| WDFY4 | 57705 | 1 | 0 | 0 |
| WDR24 | 84219 | 1 | 0 | 0 |
| WNK2 | 65268 | 1 | 0 | 0 |
| XDH | 7498 | 1 | 0 | 0 |
| XPO7 | 23039 | 1 | 0 | 0 |
| YWHAH | 7533 | 1 | 0 | 0 |
| ZC3H6 | 376940 | 1 | 0 | 0 |
| ZFPM1 | 161882 | 1 | 0 | 0 |
| ZIK1 | 284307 | 1 | 0 | 0 |
| ZMAT4 | 79698 | 1 | 0 | 0 |
| ZMYND8 | 23613 | 1 | 0 | 0 |
| ZNF160 | 90338 | 1 | 0 | 0 |
| ZNF280D | 54816 | 0 | 1 | 0 |
| ZNF295 | 49854 | 1 | 0 | 0 |
| ZNF366 | 167465 | 1 | 0 | 0 |
| ZNF497 | 162968 | 2 | 0 | 0 |
| ZNF507 | 22847 | 1 | 0 | 0 |
| ZNF67P | 7618 | 1 | 0 | 0 |
| ZNF683 | 257101 | 1 | 0 | 0 |
| ZNF687 | 57592 | 2 | 0 | 0 |
| ZSCAN2 | 54993 | 1 | 0 | 0 |
