# Supplementary material for: Abrogation of PIK3CA or PIK3R1 reduces proliferation, migration, and invasion in glioblastoma multiforme cells
Source: Oncotarget. 2011 Nov 5;2(11):833–49. doi: 10.18632/oncotarget.346 (PMC3260001; doi:10.18632/oncotarget.346)
Supplement: Supplementary file 4 [file oncotarget-02-833-s004.docx]

**Table S3.** Stringent list of 59 genes analyzed in this study. The mutations column represents the number of mutations found by Parsons, et al. [6], and the deletions and amplifications columns represent those found by both Parsons, et al. [6] and Rao, et al. [33].

| **Gene Name** | **Entrez ID** | **Mutations** | **Deletions** | **Amplifications** |
| --- | --- | --- | --- | --- |
| ABL2 | 27 | 1 | 0 | 0 |
| CDK4 | 1019 | 0 | 0 | 2 |
| CDKN2A | 1029 | 0 | 10 - Parsons, et al.; 46.4% - Rao, et al. | 0 |
| CDKN2B | 1030 | 0 | 46.4% - Rao, et al. | 0 |
| CDKN2C | 1031 | 0 | 4.6% - Rao, et al. | 0 |
| COL3A1 | 1281 | 3 | 0 | 0 |
| CYP2C19 | 1557 | 1 | 0 | 0 |
| MEGF6 | 1953 | 0 | 1 | 0 |
| EGFR | 1956 | 2 | 0 | 5 |
| F2RL1 | 2150 | 1 | 0 | 0 |
| GLI1 | 2735 | 0 | 0 | 13.4% - Rao, et al. |
| GML | 2765 | 1 | 1 | 0 |
| GRB10 | 2887 | 0 | 0 | 1 |
| HLA-DRB5 | 3127 | 0 | 1 | 0 |
| HLA-DRB9 | 3132 | 2 | 0 | 0 |
| IDH1 | 3417 | 5 | 0 | 0 |
| IL4R | 3566 | 0 | 1 | 0 |
| LBP | 3929 | 1 | 0 | 0 |
| LRP2 | 4036 | 4 | 0 | 0 |
| MDM2 | 4193 | 0 | 0 | 9.2% - Rao, et al. |
| MDM4 | 4194 | 0 | 0 | 1- Parsons, et al.; 7.7% - Rao et al. |
| MYCN | 4613 | 0 | 0 | 1 |
| NF1 | 4763 | 3 | 2.2% - Rao, et al. | 0 |
| PDGFRA | 5156 | 1 | 0 | 7.7% - Rao, et al. |
| PHF2 | 5253 | 1 | 0 | 0 |
| PIK3C2B | 5287 | 0 | 0 | 7.7% - Rao, et al. |
| PIK3CA | 5290 | 2 | 0 | 0 |
| PIK3R1 | 5295 | 2 | 0 | 0 |
| PTEN | 5728 | 5 | 1 - Parsons, et al.; 10.9% - Rao, et al. | 0 |
| PTPRM | 5797 | 1 | 0 | 0 |
| RAP1B | 5908 | 0 | 0 | 1 |
| RB1 | 5925 | 2 | 1 - Parsons, et al.; 6.3% - Rao, et al. | 0 |
| SKP2 | 6502 | 2 | 0 | 0 |
| TP53 | 7157 | 12 | 1 - Parsons, et al.; 1.9% - Rao, et al. | 0 |
| ST8SIA4 | 7903 | 1 | 0 | 0 |
| ABCC3 | 8714 | 1 | 0 | 0 |
| ARNT2 | 9915 | 2 | 0 | 0 |
| SPRY2 | 10253 | 0 | 6.3% - Rao, et al. | 0 |
| SLC30A9 | 10463 | 1 | 0 | 0 |
| CAMTA1 | 23261 | 0 | 4.6% - Rao, et al. | 0 |
| STK39 | 27347 | 1 | 0 | 0 |
| LRP1B | 53353 | 0 | 2 | 0 |
| RIPK4 | 54101 | 1 | 0 | 0 |
| RBM27 | 54439 | 2 | 0 | 0 |
| A2BP1 | 54715 | 0 | 1 | 0 |
| ZNF280D | 54816 | 0 | 1 | 0 |
| PHIP | 55023 | 2 | 0 | 0 |
| BSDC1 | 55108 | 1 | 0 | 0 |
| ZNF687 | 57592 | 2 | 0 | 0 |
| IRX6 | 79190 | 3 | 0 | 0 |
| KIAA1804 | 84451 | 2 | 0 | 0 |
| IMP4 | 92856 | 2 | 0 | 0 |
| TMEM132B | 114795 | 0 | 1 | 0 |
| SERPINA12 | 145264 | 2 | 0 | 0 |
| PHF13 | 148479 | 0 | 0 | 1 |
| TSNARE1 | 203062 | 0 | 1 | 0 |
| C6orf170 | 221322 | 2 | 0 | 0 |
| OR2L13 | 284521 | 2 | 0 | 0 |
| LOC643677 | 643677 | 2 | 0 | 0 |
