# Supplementary material for: Abrogation of PIK3CA or PIK3R1 reduces proliferation, migration, and invasion in glioblastoma multiforme cells
Source: Oncotarget. 2011 Nov 5;2(11):833–49. doi: 10.18632/oncotarget.346 (PMC3260001; doi:10.18632/oncotarget.346)
Supplement: Supplementary file 5 [file oncotarget-02-833-s005.docx]

**Table S4.** Gene ontologies enriched for mutations in GBM according to analysis of the full GBM mutation list (703 mutations) in Partek Genomics Suite. Ontologies that have an Enrichment Score of 3 or greater (p-value ≤ 0.05) are included in this table.

| **Partek - Ontology** | **Enrichment Score** | **p-value** |
| --- | --- | --- |
| membrane part | 23.202 | 8.38E-11 |
| collagen type IV | 23.034 | 9.92E-11 |
| voltage-gated sodium channel activity | 20.9112 | 8.29E-10 |
| voltage-gated cation channel activity | 20.9058 | 8.33E-10 |
| extracellular matrix part | 19.5958 | 3.09E-09 |
| sheet-forming collagen | 18.9657 | 5.80E-09 |
| phosphoinositide 3-kinase cascade | 18.9657 | 5.80E-09 |
| negative regulation of complement activation | 17.6885 | 2.08E-08 |
| regulation of cholesterol storage | 17.6885 | 2.08E-08 |
| positive regulation of cholesterol storage | 17.6885 | 2.08E-08 |
| cellular polysaccharide catabolic process | 17.6885 | 2.08E-08 |
| extrinsic to plasma membrane | 17.6885 | 2.08E-08 |
| nitric-oxide synthase regulator activity | 17.6885 | 2.08E-08 |
| urea transport | 17.6885 | 2.08E-08 |
| amide transport | 17.6885 | 2.08E-08 |
| plasma membrane part | 15.9152 | 1.22E-07 |
| insulin receptor binding | 15.7414 | 1.46E-07 |
| negative regulation of developmental process | 15.6817 | 1.55E-07 |
| negative regulation of phosphorylation | 14.3501 | 5.86E-07 |
| receptor activity | 13.5383 | 1.32E-06 |
| syncytium formation by plasma membrane fusion | 13.1231 | 2.00E-06 |
| myoblast fusion | 13.1231 | 2.00E-06 |
| cyclin-dependent protein kinase holoenzyme complex | 13.1231 | 2.00E-06 |
| visual learning | 13.1231 | 2.00E-06 |
| negative regulation of protein kinase B signaling cascade | 13.1231 | 2.00E-06 |
| maintenance of organ identity | 13.1231 | 2.00E-06 |
| positive regulation of macrophage activation | 13.1231 | 2.00E-06 |
| negative regulation of focal adhesion formation | 13.1231 | 2.00E-06 |
| metal ion transport | 12.9422 | 2.39E-06 |
| transmembrane receptor activity | 12.86 | 2.60E-06 |
| transmembrane receptor protein tyrosine kinase activity | 12.5851 | 3.42E-06 |
| transporter activity | 12.164 | 5.21E-06 |
| ATP-binding cassette (ABC) transporter complex | 12.1489 | 5.29E-06 |
| ion transmembrane transporter activity | 12.1426 | 5.33E-06 |
| sodium ion transport | 12.1332 | 5.38E-06 |
| negative regulation of phosphate metabolic process | 12.1213 | 5.44E-06 |
| transmembrane receptor protein kinase activity | 11.918 | 6.67E-06 |
| collagen | 11.6159 | 9.02E-06 |
| intrinsic to plasma membrane | 11.584 | 9.31E-06 |
| transport | 11.4375 | 1.08E-05 |
| integral to plasma membrane | 11.3948 | 1.13E-05 |
| cation channel activity | 11.3193 | 1.21E-05 |
| cationtransmembrane transporter activity | 11.0063 | 1.66E-05 |
| collagen binding | 10.882 | 1.88E-05 |
| metal ion transmembrane transporter activity | 10.7993 | 2.04E-05 |
| cytosolic calcium ion transport | 10.7778 | 2.09E-05 |
| estrogen receptor signaling pathway | 10.7778 | 2.09E-05 |
| platelet alpha granule lumen | 10.6765 | 2.31E-05 |
| regulation of cell-substrate adhesion | 10.3625 | 3.16E-05 |
| cellular carbohydrate catabolic process | 10.3625 | 3.16E-05 |
| insulin-like growth factor receptor signaling pathway | 10.3625 | 3.16E-05 |
| mitochondrion localization, microtubule-mediated | 10.3625 | 3.16E-05 |
| mitochondrion transport along microtubule | 10.3625 | 3.16E-05 |
| establishment of mitochondrion localization | 10.3625 | 3.16E-05 |
| phosphatidylinositol transporter activity | 10.3625 | 3.16E-05 |
| regulation of focal adhesion formation | 10.3625 | 3.16E-05 |
| cytoskeletal anchoring at plasma membrane | 10.3625 | 3.16E-05 |
| protein-glutamine gamma-glutamyltransferase activity | 10.3625 | 3.16E-05 |
| signal transducer activity | 10.0455 | 4.34E-05 |
| molecular transducer activity | 10.0455 | 4.34E-05 |
| intracellular receptor-mediated signaling pathway | 9.99102 | 4.58E-05 |
| cytoplasmic membrane-bounded vesicle lumen | 9.84581 | 5.30E-05 |
| regulation of developmental process | 9.7204 | 6.00E-05 |
| regulation of glucose transport | 9.70315 | 6.11E-05 |
| regulation of glucose import | 9.70315 | 6.11E-05 |
| regulation of endothelial cell migration | 9.65329 | 6.42E-05 |
| regulation of blood vessel endothelial cell migration | 9.65329 | 6.42E-05 |
| phosphoinositide 3-kinase activity | 9.65329 | 6.42E-05 |
| substrate-specific transporter activity | 9.14608 | 0.000106637 |
| mRNA export from nucleus | 9.06622 | 0.000115502 |
| mRNA transport | 9.06622 | 0.000115502 |
| positive regulation of phosphorylation | 9.06622 | 0.000115502 |
| negative regulation of angiogenesis | 9.06622 | 0.000115502 |
| transcription factor binding | 9.00027 | 0.000123377 |
| actin filament binding | 8.87859 | 0.000139341 |
| G1 phase | 8.71421 | 0.000164236 |
| peptide cross-linking | 8.71421 | 0.000164236 |
| negative regulation of response to stimulus | 8.71421 | 0.000164236 |
| positive regulation of glucose import | 8.71421 | 0.000164236 |
| basement membrane | 8.71421 | 0.000164236 |
| establishment of localization | 8.71197 | 0.000164604 |
| cation transport | 8.66942 | 0.000171758 |
| steroid hormone receptor signaling pathway | 8.64915 | 0.000175275 |
| regulation of phosphate metabolic process | 8.62887 | 0.000178866 |
| regulation of phosphorus metabolic process | 8.62887 | 0.000178866 |
| regulation of biological quality | 8.59443 | 0.000185134 |
| laminin binding | 8.50844 | 0.000201759 |
| positive regulation of cyclin-dependent protein kinase activity | 8.50844 | 0.000201759 |
| Golgi to endosome transport | 8.50844 | 0.000201759 |
| visual behavior | 8.50844 | 0.000201759 |
| cytoplasmic dynein complex | 8.50844 | 0.000201759 |
| dynein complex | 8.50844 | 0.000201759 |
| holo TFIIH complex | 8.50844 | 0.000201759 |
| positive regulation of blood vessel endothelial cell migration | 8.50844 | 0.000201759 |
| vascular endothelial growth factor receptor activity | 8.50844 | 0.000201759 |
| glycine binding | 8.50844 | 0.000201759 |
| negative regulation of S phase of mitotic cell cycle | 8.50844 | 0.000201759 |
| postsynaptic membrane | 8.50844 | 0.000201759 |
| calcium-release channel activity | 8.50844 | 0.000201759 |
| glutamate receptor activity | 8.49285 | 0.000204928 |
| heart development | 8.46467 | 0.000210785 |
| G1/S transition of mitotic cell cycle | 8.46467 | 0.000210785 |
| cell projection part | 8.46467 | 0.000210785 |
| ion transport | 8.25957 | 0.000258769 |
| cell cycle process | 8.16047 | 0.000285729 |
| regulation of cell motion | 7.9635 | 0.000347933 |
| negative regulation of protein catabolic process | 7.91802 | 0.000364121 |
| response to alkaloid | 7.91802 | 0.000364121 |
| SWI/SNF complex | 7.91802 | 0.000364121 |
| SWI/SNF-type complex | 7.91802 | 0.000364121 |
| calcium ion transport | 7.84737 | 0.000390777 |
| negative regulation of cell motion | 7.71773 | 0.000444871 |
| actin binding | 7.65431 | 0.000473999 |
| positive regulation of phosphate metabolic process | 7.50231 | 0.000551808 |
| regulation of epithelial cell proliferation | 7.50231 | 0.000551808 |
| positive regulation of fibroblast proliferation | 7.23434 | 0.000721383 |
| negative regulation of endothelial cell proliferation | 7.23434 | 0.000721383 |
| organic anion transmembrane transporter activity | 7.17486 | 0.000765595 |
| basal lamina | 7.17486 | 0.000765595 |
| apical part of cell | 7.17486 | 0.000765595 |
| negative regulation of blood vessel endothelial cell migration | 7.17486 | 0.000765595 |
| fibrinogen complex | 7.17486 | 0.000765595 |
| Wnt receptor activity | 7.17486 | 0.000765595 |
| osteoblast differentiation | 7.17486 | 0.000765595 |
| negative regulation of cell differentiation | 7.17486 | 0.000765595 |
| regulation of respiratory burst | 7.17486 | 0.000765595 |
| regulation of oxygen and reactive oxygen species metabolic process | 7.17486 | 0.000765595 |
| ankyrin binding | 7.17486 | 0.000765595 |
| vinculin binding | 7.17486 | 0.000765595 |
| receptor-mediated endocytosis | 7.06239 | 0.00085673 |
| maintenance of protein location in cell | 6.64082 | 0.00130595 |
| substrate-specific transmembrane transporter activity | 6.3324 | 0.00177777 |
| ligand-gated ion channel activity | 6.21793 | 0.00199336 |
| ligand-gated channel activity | 6.21793 | 0.00199336 |
| microtubule | 6.21793 | 0.00199336 |
| G-protein coupled receptor activity | 6.20851 | 0.00201224 |
| cell part | 6.20737 | 0.00201453 |
| enzyme binding | 6.19027 | 0.00204928 |
| negative regulation of proteolysis | 6.16805 | 0.00209532 |
| muscarinic acetylcholine receptor signaling pathway | 6.16805 | 0.00209532 |
| extracellular matrix binding | 6.16805 | 0.00209532 |
| cyclin-dependent protein kinase inhibitor activity | 6.16805 | 0.00209532 |
| platelet-derived growth factor receptor binding | 6.16805 | 0.00209532 |
| negative regulation of insulin receptor signaling pathway | 6.16805 | 0.00209532 |
| solute:cationantiporter activity | 6.16805 | 0.00209532 |
| transferase activity, transferring amino-acyl groups | 6.16805 | 0.00209532 |
| regulation of fibroblast proliferation | 6.12073 | 0.00219686 |
| collagen fibril organization | 6.12073 | 0.00219686 |
| RNA transport | 5.97888 | 0.00253167 |
| establishment of RNA localization | 5.97888 | 0.00253167 |
| voltage-gated potassium channel activity | 5.97888 | 0.00253167 |
| cellular_component | 5.83574 | 0.00292126 |
| PML body | 5.66882 | 0.00345195 |
| specific transcriptional repressor activity | 5.66119 | 0.00347837 |
| cell junction | 5.5239 | 0.00399026 |
| negative regulation of cell migration | 5.38105 | 0.00460297 |
| peptidyl-tyrosine phosphorylation | 5.38007 | 0.00460748 |
| regulation of lipid storage | 5.38007 | 0.00460748 |
| calcium ion transport into cytosol | 5.38007 | 0.00460748 |
| G1 phase of mitotic cell cycle | 5.38007 | 0.00460748 |
| negative regulation of epithelial cell proliferation | 5.38007 | 0.00460748 |
| positive regulation of transforming growth factor beta receptor signaling pathway | 5.38007 | 0.00460748 |
| anchoring collagen | 5.38007 | 0.00460748 |
| positive regulation of chemotaxis | 5.38007 | 0.00460748 |
| positive regulation of proteolysis | 5.38007 | 0.00460748 |
| regulation of macrophage activation | 5.38007 | 0.00460748 |
| protein ubiquitination during ubiquitin-dependent protein catabolic process | 5.38007 | 0.00460748 |
| 1-phosphatidylinositol-3-kinase activity | 5.38007 | 0.00460748 |
| regulation of GTPase activity | 5.38007 | 0.00460748 |
| spectrin | 5.38007 | 0.00460748 |
| regulation of cellular metabolic process | 5.30856 | 0.00494905 |
| homeostatic process | 5.24325 | 0.00528308 |
| response to calcium ion | 5.1133 | 0.00601618 |
| cell surface receptor linked signal transduction | 5.1059 | 0.00606089 |
| cytoplasmic vesicle part | 5.03615 | 0.00649871 |
| extracellular region part | 4.92693 | 0.00724874 |
| transcription factor complex | 4.90379 | 0.0074184 |
| extracellular matrix organization | 4.86358 | 0.00772281 |
| chromatin remodeling complex | 4.86358 | 0.00772281 |
| protein complex binding | 4.85655 | 0.00777727 |
| extracellular structure organization | 4.76572 | 0.00851676 |
| interleukin-8 binding | 4.75671 | 0.00859381 |
| receptor clustering | 4.75671 | 0.00859381 |
| odontogenesis of dentine-containing tooth | 4.75671 | 0.00859381 |
| bile acid and bile salt transport | 4.75671 | 0.00859381 |
| transcription initiation from RNA polymerase III promoter | 4.75671 | 0.00859381 |
| cyclin binding | 4.75671 | 0.00859381 |
| negative regulation of cell-matrix adhesion | 4.75671 | 0.00859381 |
| negative regulation of cell-substrate adhesion | 4.75671 | 0.00859381 |
| chondroitin 4-sulfotransferase activity | 4.75671 | 0.00859381 |
| centrosome separation | 4.75671 | 0.00859381 |
| collagen biosynthetic process | 4.75671 | 0.00859381 |
| positive regulation of synaptic transmission | 4.75671 | 0.00859381 |
| triglyceride catabolic process | 4.75671 | 0.00859381 |
| cobalamin transport | 4.75671 | 0.00859381 |
| dopamine receptor activity, coupled via Gi/Go | 4.75671 | 0.00859381 |
| catecholamine metabolic process | 4.75671 | 0.00859381 |
| catechol metabolic process | 4.75671 | 0.00859381 |
| negative regulation of response to external stimulus | 4.75671 | 0.00859381 |
| circadian regulation of gene expression | 4.75671 | 0.00859381 |
| diol metabolic process | 4.75671 | 0.00859381 |
| response to histamine | 4.75671 | 0.00859381 |
| regulation of multicellular organism growth | 4.75671 | 0.00859381 |
| response to cocaine | 4.75671 | 0.00859381 |
| dopamine metabolic process | 4.75671 | 0.00859381 |
| behavioral response to cocaine | 4.75671 | 0.00859381 |
| regulation of dopamine uptake | 4.75671 | 0.00859381 |
| regulation of catecholamine uptake during transmission of nerve impulse | 4.75671 | 0.00859381 |
| prepulse inhibition | 4.75671 | 0.00859381 |
| eukaryotic translation elongation factor 1 complex | 4.75671 | 0.00859381 |
| positive regulation of cyclin-dependent protein kinase activity during G1/S | 4.75671 | 0.00859381 |
| response to UV-A | 4.75671 | 0.00859381 |
| phosphodiesterase I activity | 4.75671 | 0.00859381 |
| bubble DNA binding | 4.75671 | 0.00859381 |
| nucleotide-excision repair, DNA incision, 3'-to lesion | 4.75671 | 0.00859381 |
| regulation of blood coagulation | 4.75671 | 0.00859381 |
| positive regulation of vascular endothelial growth factor receptor signaling pathway | 4.75671 | 0.00859381 |
| cellular phosphate ion homeostasis | 4.75671 | 0.00859381 |
| di-, tri-valent inorganic anion homeostasis | 4.75671 | 0.00859381 |
| phosphate ion homeostasis | 4.75671 | 0.00859381 |
| anion homeostasis | 4.75671 | 0.00859381 |
| parathyroid gland development | 4.75671 | 0.00859381 |
| N-acetyllactosaminide beta-1,6-N-acetylglucosaminyltransferase activity | 4.75671 | 0.00859381 |
| positive regulation of appetite | 4.75671 | 0.00859381 |
| regulation of interleukin-1 production | 4.75671 | 0.00859381 |
| negative regulation of interleukin-6 biosynthetic process | 4.75671 | 0.00859381 |
| glycerol kinase activity | 4.75671 | 0.00859381 |
| extracellular-glycine-gated ion channel activity | 4.75671 | 0.00859381 |
| extracellular-glycine-gated chloride channel activity | 4.75671 | 0.00859381 |
| hemoglobin binding | 4.75671 | 0.00859381 |
| positive regulation of receptor biosynthetic process | 4.75671 | 0.00859381 |
| negative regulation of MHC class II biosynthetic process | 4.75671 | 0.00859381 |
| insulin receptor complex | 4.75671 | 0.00859381 |
| positive regulation of insulin receptor signaling pathway | 4.75671 | 0.00859381 |
| positive regulation of response to stimulus | 4.75671 | 0.00859381 |
| inositol 1,3,4,5 tetrakisphosphate binding | 4.75671 | 0.00859381 |
| dendritic spine | 4.75671 | 0.00859381 |
| detection of biotic stimulus | 4.75671 | 0.00859381 |
| positive regulation of interleukin-8 production | 4.75671 | 0.00859381 |
| positive regulation of tumor necrosis factor production | 4.75671 | 0.00859381 |
| bacterial cell surface binding | 4.75671 | 0.00859381 |
| positive regulation of respiratory burst | 4.75671 | 0.00859381 |
| positive regulation of sequestering of triglyceride | 4.75671 | 0.00859381 |
| chylomicron remodeling | 4.75671 | 0.00859381 |
| negative regulation of MAPKKK cascade | 4.75671 | 0.00859381 |
| transmission of nerve impulse | 4.75671 | 0.00859381 |
| negative regulation of myoblast differentiation | 4.75671 | 0.00859381 |
| calmodulin-dependent cyclic-nucleotide phosphodiesterase activity | 4.75671 | 0.00859381 |
| platelet-derived growth factor receptor activity | 4.75671 | 0.00859381 |
| protein retention in ER lumen | 4.75671 | 0.00859381 |
| ErbB-3 class receptor binding | 4.75671 | 0.00859381 |
| insulin binding | 4.75671 | 0.00859381 |
| DNA-dependent protein kinase-DNA ligase 4 complex | 4.75671 | 0.00859381 |
| nonhomologous end joining complex | 4.75671 | 0.00859381 |
| positive regulation of Cdc42 GTPase activity | 4.75671 | 0.00859381 |
| protein kinase A catalytic subunit binding | 4.75671 | 0.00859381 |
| adult heart development | 4.75671 | 0.00859381 |
| endothelial cell differentiation | 4.75671 | 0.00859381 |
| protein anchor | 4.75671 | 0.00859381 |
| sodium ion transmembrane transporter activity | 4.75671 | 0.00859381 |
| calcium ion transmembrane transporter activity | 4.75671 | 0.00859381 |
| regulation of dephosphorylation | 4.75671 | 0.00859381 |
| protein disulfide oxidoreductase activity | 4.75671 | 0.00859381 |
| fibronectin binding | 4.75671 | 0.00859381 |
| regulation of immune system process | 4.75671 | 0.00859381 |
| regulation of leukocyte chemotaxis | 4.75671 | 0.00859381 |
| negative regulation of interleukin-12 production | 4.75671 | 0.00859381 |
| regulation of transforming growth factor-beta1 production | 4.75671 | 0.00859381 |
| muscle thin filament assembly | 4.75671 | 0.00859381 |
| cardiac muscle fiber development | 4.75671 | 0.00859381 |
| cardiac myofibril assembly | 4.75671 | 0.00859381 |
| glucocorticoid receptor binding | 4.75671 | 0.00859381 |
| cellular iron ion homeostasis | 4.74596 | 0.00868669 |
| plasma membrane fusion | 4.74596 | 0.00868669 |
| centriole | 4.74596 | 0.00868669 |
| alcohol binding | 4.74596 | 0.00868669 |
| mitotic spindle organization | 4.74596 | 0.00868669 |
| regulation of positive chemotaxis | 4.74596 | 0.00868669 |
| positive regulation of positive chemotaxis | 4.74596 | 0.00868669 |
| cell soma | 4.74596 | 0.00868669 |
| transforming growth factor beta binding | 4.74596 | 0.00868669 |
| cell-cell signaling | 4.56907 | 0.0103676 |
| negative regulation of MAP kinase activity | 4.55598 | 0.0105042 |
| maintenance of location in cell | 4.55598 | 0.0105042 |
| protein binding | 4.50078 | 0.0111003 |
| regulation of protein kinase activity | 4.38781 | 0.0124279 |
| cell communication | 4.28574 | 0.0137634 |
| regulation of behavior | 4.25722 | 0.0141616 |
| regulation of chemotaxis | 4.25722 | 0.0141616 |
| cell cortex part | 4.25722 | 0.0141616 |
| regulation of kinase activity | 4.2415 | 0.014386 |
| iron ion homeostasis | 4.22425 | 0.0146363 |
| peptidyl-tyrosine modification | 4.22425 | 0.0146363 |
| positive regulation of nitric oxide biosynthetic process | 4.22425 | 0.0146363 |
| extrinsic to membrane | 4.22425 | 0.0146363 |
| insulin-like growth factor receptor binding | 4.22425 | 0.0146363 |
| phosphate binding | 4.22425 | 0.0146363 |
| solute:soluteantiporter activity | 4.22425 | 0.0146363 |
| response to hypoxia | 4.21445 | 0.0147805 |
| di-, tri-valent inorganic cation homeostasis | 4.14032 | 0.0159178 |
| muscle contraction | 4.13616 | 0.0159841 |
| gated channel activity | 4.06242 | 0.0172073 |
| cell projection | 4.05686 | 0.0173032 |
| organ development | 4.03792 | 0.0176342 |
| chromatin remodeling | 4.0134 | 0.0180718 |
| voltage-gated calcium channel activity | 3.98545 | 0.018584 |
| regulation of MAP kinase activity | 3.98545 | 0.018584 |
| protein phosphatase binding | 3.98545 | 0.018584 |
| nuclear matrix | 3.98545 | 0.018584 |
| intrinsic to membrane | 3.97126 | 0.0188496 |
| anatomical structure development | 3.90126 | 0.0202165 |
| ATPase activity, coupled | 3.89512 | 0.0203409 |
| cytoskeletal protein binding | 3.87375 | 0.0207803 |
| protein amino acid autophosphorylation | 3.83172 | 0.0216723 |
| biological_process | 3.81696 | 0.0219946 |
| chemical homeostasis | 3.79499 | 0.0224831 |
| odontogenesis | 3.7872 | 0.022659 |
| positive regulation of protein amino acid phosphorylation | 3.7872 | 0.022659 |
| regulation of transmission of nerve impulse | 3.7872 | 0.022659 |
| response to ethanol | 3.7872 | 0.022659 |
| nucleotide-excision repair | 3.7872 | 0.022659 |
| positive regulation of angiogenesis | 3.7872 | 0.022659 |
| amino acid binding | 3.7872 | 0.022659 |
| photoreceptor cell maintenance | 3.7872 | 0.022659 |
| ionotropic glutamate receptor activity | 3.7872 | 0.022659 |
| positive regulation of protein catabolic process | 3.7872 | 0.022659 |
| delayed rectifier potassium channel activity | 3.7872 | 0.022659 |
| carboxylic acid biosynthetic process | 3.7872 | 0.022659 |
| ATPase activity, coupled to movement of substances | 3.77778 | 0.0228733 |
| response to oxygen levels | 3.77778 | 0.0228733 |
| cell-substrate adherens junction | 3.77778 | 0.0228733 |
| focal adhesion | 3.77778 | 0.0228733 |
| muscle system process | 3.77702 | 0.0228907 |
| cation homeostasis | 3.74073 | 0.0237368 |
| developmental process | 3.73747 | 0.0238143 |
| neuropeptide signaling pathway | 3.73721 | 0.0238204 |
| positive regulation of DNA replication | 3.73721 | 0.0238204 |
| regulation of multicellular organismal process | 3.73624 | 0.0238436 |
| cell-cell junction | 3.66035 | 0.0257236 |
| positive regulation of cell migration | 3.66035 | 0.0257236 |
| cell cycle arrest | 3.51974 | 0.0296071 |
| C-X-C chemokine binding | 3.51875 | 0.0296365 |
| interleukin-1 binding | 3.51875 | 0.0296365 |
| citrate metabolic process | 3.51875 | 0.0296365 |
| transferase activity, transferring acyl groups, acyl groups converted into alkyl on transfer | 3.51875 | 0.0296365 |
| cartilage condensation | 3.51875 | 0.0296365 |
| intermediate-density lipoprotein particle | 3.51875 | 0.0296365 |
| low-density lipoprotein particle clearance | 3.51875 | 0.0296365 |
| DNA packaging | 3.51875 | 0.0296365 |
| phospholipid-translocating ATPase activity | 3.51875 | 0.0296365 |
| positive regulation of cell-matrix adhesion | 3.51875 | 0.0296365 |
| positive regulation of cell-substrate adhesion | 3.51875 | 0.0296365 |
| negative regulation of osteoblast differentiation | 3.51875 | 0.0296365 |
| negative regulation of T cell proliferation | 3.51875 | 0.0296365 |
| negative regulation of mast cell proliferation | 3.51875 | 0.0296365 |
| cellular response to nutrient | 3.51875 | 0.0296365 |
| collagen metabolic process | 3.51875 | 0.0296365 |
| positive regulation of transmission of nerve impulse | 3.51875 | 0.0296365 |
| nitrogen compound biosynthetic process | 3.51875 | 0.0296365 |
| acylglycerol catabolic process | 3.51875 | 0.0296365 |
| cilium axoneme | 3.51875 | 0.0296365 |
| cilium part | 3.51875 | 0.0296365 |
| dopamine receptor activity | 3.51875 | 0.0296365 |
| inhibition of adenylatecyclase activity by dopamine receptor signaling pathway | 3.51875 | 0.0296365 |
| response to organic nitrogen | 3.51875 | 0.0296365 |
| response to amine stimulus | 3.51875 | 0.0296365 |
| social behavior | 3.51875 | 0.0296365 |
| dopamine binding | 3.51875 | 0.0296365 |
| negative regulation of blood pressure | 3.51875 | 0.0296365 |
| AP-2 adaptor complex | 3.51875 | 0.0296365 |
| activation of phospholipase A2 activity | 3.51875 | 0.0296365 |
| response to UV-C | 3.51875 | 0.0296365 |
| regulation of cardiac muscle cell proliferation | 3.51875 | 0.0296365 |
| regulation of vascular endothelial growth factor receptor signaling pathway | 3.51875 | 0.0296365 |
| glutamate catabolic process | 3.51875 | 0.0296365 |
| extracellular ligand-gated ion channel activity | 3.51875 | 0.0296365 |
| excitatory extracellular ligand-gated ion channel activity | 3.51875 | 0.0296365 |
| DNA damage response, signal transduction by p53 class mediator resulting in cell cycle arrest | 3.51875 | 0.0296365 |
| negative regulation of glucose import | 3.51875 | 0.0296365 |
| negative regulation of protein amino acid phosphorylation | 3.51875 | 0.0296365 |
| release of sequestered calcium ion into cytosol | 3.51875 | 0.0296365 |
| interferon binding | 3.51875 | 0.0296365 |
| regulation of cellular carbohydrate metabolic process | 3.51875 | 0.0296365 |
| regulation of glucose metabolic process | 3.51875 | 0.0296365 |
| positive regulation of glucose metabolic process | 3.51875 | 0.0296365 |
| positive regulation of fatty acid beta-oxidation | 3.51875 | 0.0296365 |
| kinetochore microtubule | 3.51875 | 0.0296365 |
| positive regulation of interleukin-6 production | 3.51875 | 0.0296365 |
| positive regulation of proteasomal ubiquitin-dependent protein catabolic process | 3.51875 | 0.0296365 |
| basal plasma membrane | 3.51875 | 0.0296365 |
| positive regulation of neuron apoptosis | 3.51875 | 0.0296365 |
| negative regulation of muscle cell differentiation | 3.51875 | 0.0296365 |
| oxidoreductase activity, acting on the aldehyde or oxo group of donors, disulfide as acceptor | 3.51875 | 0.0296365 |
| growth hormone receptor signaling pathway | 3.51875 | 0.0296365 |
| receptor tyrosine kinase binding | 3.51875 | 0.0296365 |
| lipid phosphatase activity | 3.51875 | 0.0296365 |
| regulation of hydrogen peroxide metabolic process | 3.51875 | 0.0296365 |
| regulation of Cdc42 GTPase activity | 3.51875 | 0.0296365 |
| leukocyte tethering or rolling | 3.51875 | 0.0296365 |
| COPII vesicle coat | 3.51875 | 0.0296365 |
| sodium channel regulator activity | 3.51875 | 0.0296365 |
| glycine hydroxymethyltransferase activity | 3.51875 | 0.0296365 |
| potassium:chloridesymporter activity | 3.51875 | 0.0296365 |
| monovalent cation:hydrogenantiporter activity | 3.51875 | 0.0296365 |
| sodium:hydrogenantiporter activity | 3.51875 | 0.0296365 |
| cohesin complex | 3.51875 | 0.0296365 |
| protein phosphatase 2A binding | 3.51875 | 0.0296365 |
| calcium channel regulator activity | 3.51875 | 0.0296365 |
| negative regulation of plasma membrane long-chain fatty acid transport | 3.51875 | 0.0296365 |
| fibroblast growth factor binding | 3.51875 | 0.0296365 |
| negative regulation of fibrinolysis | 3.51875 | 0.0296365 |
| cellular response to glucose starvation | 3.51875 | 0.0296365 |
| myofibril assembly | 3.51875 | 0.0296365 |
| oxidoreductase activity, acting on CH or CH2 groups | 3.51875 | 0.0296365 |
| protein kinase inhibitor activity | 3.50958 | 0.0299095 |
| cell activation | 3.50958 | 0.0299095 |
| negative regulation of adenylatecyclase activity | 3.50958 | 0.0299095 |
| negative regulation of lyase activity | 3.50958 | 0.0299095 |
| regulation of cell migration | 3.50958 | 0.0299095 |
| negative regulation of protein metabolic process | 3.49845 | 0.0302442 |
| amine binding | 3.49845 | 0.0302442 |
| G-protein coupled receptor protein signaling pathway | 3.46578 | 0.0312485 |
| biological regulation | 3.46172 | 0.0313756 |
| organ morphogenesis | 3.4503 | 0.0317362 |
| insoluble fraction | 3.44545 | 0.0318904 |
| integral to membrane | 3.43114 | 0.0323499 |
| structural constituent of cytoskeleton | 3.41697 | 0.0328118 |
| protein serine/threonine kinase inhibitor activity | 3.41556 | 0.0328581 |
| regulation of neurological system process | 3.41556 | 0.0328581 |
| regulation of protein kinase B signaling cascade | 3.41556 | 0.0328581 |
| positive regulation of epithelial cell proliferation | 3.41556 | 0.0328581 |
| peptide hormone secretion | 3.41556 | 0.0328581 |
| inward rectifier potassium channel activity | 3.41556 | 0.0328581 |
| phospholipid transporter activity | 3.41556 | 0.0328581 |
| anion binding | 3.41556 | 0.0328581 |
| ion homeostasis | 3.40996 | 0.0330426 |
| regulation of transferase activity | 3.40001 | 0.0333731 |
| extracellular matrix structural constituent | 3.3938 | 0.0335809 |
| cell-substrate junction | 3.3938 | 0.0335809 |
| immune response | 3.38476 | 0.0338858 |
| RNA binding | 3.36661 | 0.0345064 |
| transmembrane transporter activity | 3.31054 | 0.0364966 |
| cellular component organization | 3.27846 | 0.0376864 |
| synaptic transmission | 3.22513 | 0.0397507 |
| regulation of system process | 3.22109 | 0.0399116 |
| positive regulation of MAP kinase activity | 3.20021 | 0.0407538 |
| voltage-gated potassium channel complex | 3.20021 | 0.0407538 |
| regulation of response to stimulus | 3.16564 | 0.0421871 |
| cellular calcium ion homeostasis | 3.15851 | 0.042489 |
| cell-cell adhesion | 3.15219 | 0.0427583 |
| phosphatase binding | 3.10676 | 0.0447455 |
| cell cycle checkpoint | 3.09553 | 0.0452511 |
| NF-kappaB binding | 3.09553 | 0.0452511 |
| response to lipopolysaccharide | 3.09553 | 0.0452511 |
| steroid hormone receptor activity | 3.09553 | 0.0452511 |
| gland development | 3.09553 | 0.0452511 |
| hormone secretion | 3.09553 | 0.0452511 |
| chloride transport | 3.09553 | 0.0452511 |
| DNA damage response, signal transduction by p53 class mediator | 3.09553 | 0.0452511 |
| B cell differentiation | 3.09553 | 0.0452511 |
| spindle pole | 3.09553 | 0.0452511 |
| negative regulation of mitotic cell cycle | 3.09553 | 0.0452511 |
| kidney development | 3.09553 | 0.0452511 |
| calcium ion homeostasis | 3.07907 | 0.0460022 |
| brain development | 3.06259 | 0.0467662 |
| positive regulation of cell motion | 3.06259 | 0.0467662 |
| negative regulation of cell growth | 3.05391 | 0.0471741 |
| multicellular organismal development | 3.04827 | 0.0474408 |
| kinase binding | 3.00165 | 0.0497052 |
| positive regulation of cell proliferation | 2.99323 | 0.0501254 |
