# Supplementary material for: Abrogation of PIK3CA or PIK3R1 reduces proliferation, migration, and invasion in glioblastoma multiforme cells
Source: Oncotarget. 2011 Nov 5;2(11):833–49. doi: 10.18632/oncotarget.346 (PMC3260001; doi:10.18632/oncotarget.346)
Supplement: Supplementary file 6 [file oncotarget-02-833-s006.docx]

**Table S5.** Gene ontologies enriched for mutations in GBM according to analysis of the stringent GBM mutation list (59 mutations) in Partek Genomics Suite. Ontologies that have an Enrichment Score of 3 or greater (p-value ≤ 0.05) are included in this table.

| **Partek - Ontology** | **p-value** | **Enrichment Score** |
| --- | --- | --- |
| regulation of cell-substrate adhesion | 3.89E-47 | 106.8620 |
| insulin receptor binding | 7.68E-44 | 99.2749 |
| negative regulation of phosphorylation | 1.66E-40 | 91.5979 |
| phosphoinositide 3-kinase cascade | 1.98E-39 | 89.1196 |
| negative regulation of phosphate metabolic process | 3.61E-35 | 79.3074 |
| osteoblast differentiation | 6.38E-34 | 76.4348 |
| G1/S transition of mitotic cell cycle | 5.54E-32 | 71.9713 |
| cyclin-dependent protein kinase inhibitor activity | 8.72E-30 | 66.9124 |
| peptidyl-tyrosine phosphorylation | 1.44E-26 | 59.4994 |
| peptidyl-tyrosine modification | 7.06E-22 | 48.7025 |
| phosphoinositide 3-kinase activity | 7.06E-22 | 48.7025 |
| negative regulation of cell-matrix adhesion | 1.56E-20 | 45.6099 |
| negative regulation of cell-substrate adhesion | 1.56E-20 | 45.6099 |
| collagen biosynthetic process | 1.56E-20 | 45.6099 |
| nitric-oxide synthase regulator activity | 1.56E-20 | 45.6099 |
| positive regulation of cyclin-dependent protein kinase activity during G1/S | 1.56E-20 | 45.6099 |
| response to UV-A | 1.56E-20 | 45.6099 |
| regulation of blood coagulation | 1.56E-20 | 45.6099 |
| detection of biotic stimulus | 1.56E-20 | 45.6099 |
| positive regulation of interleukin-8 production | 1.56E-20 | 45.6099 |
| positive regulation of tumor necrosis factor production | 1.56E-20 | 45.6099 |
| bacterial cell surface binding | 1.56E-20 | 45.6099 |
| positive regulation of respiratory burst | 1.56E-20 | 45.6099 |
| negative regulation of MAPKKK cascade | 1.56E-20 | 45.6099 |
| platelet-derived growth factor receptor activity | 1.56E-20 | 45.6099 |
| ErbB-3 class receptor binding | 1.56E-20 | 45.6099 |
| insulin binding | 1.56E-20 | 45.6099 |
| negative regulation of cell migration | 5.07E-19 | 42.1262 |
| protein serine/threonine kinase inhibitor activity | 1.26E-18 | 41.2142 |
| positive regulation of fibroblast proliferation | 2.40E-17 | 38.2683 |
| cell cycle checkpoint | 2.40E-17 | 38.2683 |
| DNA damage response, signal transduction by p53 class mediator | 2.40E-17 | 38.2683 |
| negative regulation of endothelial cell proliferation | 2.40E-17 | 38.2683 |
| negative regulation of cell motion | 3.27E-17 | 37.9597 |
| negative regulation of cell growth | 7.38E-17 | 37.1449 |
| regulation of protein stability | 1.10E-16 | 36.7436 |
| regulation of cell motion | 6.68E-16 | 34.9430 |
| regulation of cyclin-dependent protein kinase activity | 1.01E-15 | 34.5246 |
| negative regulation of T cell proliferation | 1.11E-15 | 34.4353 |
| negative regulation of mast cell proliferation | 1.11E-15 | 34.4353 |
| cellular response to nutrient | 1.11E-15 | 34.4353 |
| collagen metabolic process | 1.11E-15 | 34.4353 |
| AP-2 adaptor complex | 1.11E-15 | 34.4353 |
| activation of phospholipase A2 activity | 1.11E-15 | 34.4353 |
| DNA damage response, signal transduction by p53 class mediator resulting in cell cycle arrest | 1.11E-15 | 34.4353 |
| negative regulation of glucose import | 1.11E-15 | 34.4353 |
| positive regulation of interleukin-6 production | 1.11E-15 | 34.4353 |
| positive regulation of macrophage activation | 1.11E-15 | 34.4353 |
| positive regulation of proteasomal ubiquitin-dependent protein catabolic process | 1.11E-15 | 34.4353 |
| visual learning | 1.11E-15 | 34.4353 |
| positive regulation of neuron apoptosis | 1.11E-15 | 34.4353 |
| growth hormone receptor signaling pathway | 1.11E-15 | 34.4353 |
| lipid phosphatase activity | 1.11E-15 | 34.4353 |
| negative regulation of focal adhesion formation | 1.11E-15 | 34.4353 |
| negative regulation of protein kinase B signaling cascade | 1.11E-15 | 34.4353 |
| cellular response to glucose starvation | 1.11E-15 | 34.4353 |
| regulation of cell proliferation | 2.28E-15 | 33.7164 |
| cell cycle process | 2.32E-15 | 33.6974 |
| regulation of fibroblast proliferation | 2.90E-15 | 33.4749 |
| collagen fibril organization | 2.90E-15 | 33.4749 |
| wound healing | 2.09E-14 | 31.4985 |
| insulin receptor signaling pathway | 2.09E-14 | 31.4985 |
| cell cycle arrest | 4.91E-14 | 30.6445 |
| regulation of glucose transport | 1.21E-13 | 29.7402 |
| regulation of glucose import | 1.21E-13 | 29.7402 |
| negative regulation of protein kinase activity | 5.86E-13 | 28.1655 |
| cellular response to nutrient levels | 9.28E-13 | 27.7060 |
| skin development | 9.28E-13 | 27.7060 |
| regulation of phospholipase activity | 9.28E-13 | 27.7060 |
| regulation of phospholipase A2 activity | 9.28E-13 | 27.7060 |
| positive regulation of phospholipase A2 activity | 9.28E-13 | 27.7060 |
| epidermal cell differentiation | 9.28E-13 | 27.7060 |
| opsonization | 9.28E-13 | 27.7060 |
| positive regulation of RasGTPase activity | 9.28E-13 | 27.7060 |
| pigmentation | 9.28E-13 | 27.7060 |
| positive regulation of GTPase activity | 9.28E-13 | 27.7060 |
| eye morphogenesis | 9.28E-13 | 27.7060 |
| insulin-like growth factor receptor signaling pathway | 9.28E-13 | 27.7060 |
| response to growth hormone stimulus | 9.28E-13 | 27.7060 |
| regulation of focal adhesion formation | 9.28E-13 | 27.7060 |
| negative regulation of endothelial cell migration | 9.28E-13 | 27.7060 |
| M phase | 9.28E-13 | 27.7060 |
| regulation of lipid kinase activity | 9.28E-13 | 27.7060 |
| regulation of mitochondrial membrane permeability | 9.28E-13 | 27.7060 |
| enzyme binding | 1.11E-11 | 25.2285 |
| negative regulation of cell proliferation | 1.60E-11 | 24.8563 |
| regulation of epithelial cell proliferation | 2.81E-11 | 24.2940 |
| protein phosphatase binding | 2.81E-11 | 24.2940 |
| protein complex binding | 5.29E-11 | 23.6622 |
| regulation of phosphate metabolic process | 7.16E-11 | 23.3605 |
| regulation of phosphorus metabolic process | 7.16E-11 | 23.3605 |
| positive regulation of DNA replication | 8.19E-11 | 23.2260 |
| senescence | 8.37E-11 | 23.2036 |
| fibril organization | 8.37E-11 | 23.2036 |
| multicellular organismal macromolecule metabolic process | 8.37E-11 | 23.2036 |
| platelet-derived growth factor binding | 8.37E-11 | 23.2036 |
| negative regulation of immune response | 8.37E-11 | 23.2036 |
| positive regulation of cyclin-dependent protein kinase activity | 8.37E-11 | 23.2036 |
| protein insertion into membrane | 8.37E-11 | 23.2036 |
| positive regulation of leukocyte chemotaxis | 8.37E-11 | 23.2036 |
| regulation of interleukin-8 production | 8.37E-11 | 23.2036 |
| visual behavior | 8.37E-11 | 23.2036 |
| glial cell development | 8.37E-11 | 23.2036 |
| negative regulation of S phase of mitotic cell cycle | 8.37E-11 | 23.2036 |
| ER overload response | 8.37E-11 | 23.2036 |
| kinase binding | 1.70E-10 | 22.4962 |
| regulation of cell adhesion | 2.18E-10 | 22.2462 |
| protein kinase inhibitor activity | 2.18E-10 | 22.2462 |
| cell activation | 2.18E-10 | 22.2462 |
| cellular response to insulin stimulus | 2.18E-10 | 22.2462 |
| regulation of cell cycle | 7.25E-10 | 21.0449 |
| phosphatase binding | 1.24E-09 | 20.5104 |
| manganese ion binding | 2.11E-09 | 19.9761 |
| cellular response to extracellular stimulus | 2.11E-09 | 19.9761 |
| drug metabolic process | 2.11E-09 | 19.9761 |
| negative regulation of tumor necrosis factor production | 2.11E-09 | 19.9761 |
| macrophage activation | 2.11E-09 | 19.9761 |
| defense response to Gram-negative bacterium | 2.11E-09 | 19.9761 |
| regulation of respiratory burst | 2.11E-09 | 19.9761 |
| liver development | 2.11E-09 | 19.9761 |
| regulation of bone remodeling | 2.11E-09 | 19.9761 |
| N-glycan processing | 2.11E-09 | 19.9761 |
| oligosaccharide metabolic process | 2.11E-09 | 19.9761 |
| organic anion transmembrane transporter activity | 2.11E-09 | 19.9761 |
| PML body | 2.68E-09 | 19.7380 |
| receptor signaling protein serine/threonine kinase activity | 5.49E-09 | 19.0201 |
| heart development | 1.07E-08 | 18.3512 |
| negative regulation of cellular process | 1.63E-08 | 17.9292 |
| extracellular matrix organization | 2.00E-08 | 17.7264 |
| regulation of cell cycle process | 2.40E-08 | 17.5469 |
| G1/S transition checkpoint | 2.40E-08 | 17.5469 |
| negative regulation of insulin receptor signaling pathway | 2.40E-08 | 17.5469 |
| regulation of tissue remodeling | 2.40E-08 | 17.5469 |
| myelination | 2.40E-08 | 17.5469 |
| regulation of angiogenesis | 2.40E-08 | 17.5469 |
| platelet-derived growth factor receptor binding | 2.40E-08 | 17.5469 |
| peptide hormone binding | 2.40E-08 | 17.5469 |
| activation of caspase activity by cytochrome c | 2.40E-08 | 17.5469 |
| DNA damage response, signal transduction by p53 class mediator resulting in induction of apoptosis | 2.40E-08 | 17.5469 |
| protein heterodimerization activity | 4.58E-08 | 16.8997 |
| regulation of oxidoreductase activity | 6.22E-08 | 16.5925 |
| receptor-mediated endocytosis | 1.04E-07 | 16.0764 |
| protein kinase binding | 1.21E-07 | 15.9245 |
| positive regulation of transforming growth factor beta receptor signaling pathway | 1.60E-07 | 15.6509 |
| negative regulation of epithelial cell proliferation | 1.60E-07 | 15.6509 |
| MAP kinase kinasekinase activity | 1.60E-07 | 15.6509 |
| positive regulation of chemotaxis | 1.60E-07 | 15.6509 |
| regulation of interleukin-6 production | 1.60E-07 | 15.6509 |
| regulation of tumor necrosis factor production | 1.60E-07 | 15.6509 |
| regulation of macrophage activation | 1.60E-07 | 15.6509 |
| protein ubiquitination during ubiquitin-dependent protein catabolic process | 1.60E-07 | 15.6509 |
| positive regulation of proteolysis | 1.60E-07 | 15.6509 |
| ensheathment of neurons | 1.60E-07 | 15.6509 |
| peripheral nervous system development | 1.60E-07 | 15.6509 |
| axon ensheathment | 1.60E-07 | 15.6509 |
| 1-phosphatidylinositol-3-kinase activity | 1.60E-07 | 15.6509 |
| response to peptide hormone stimulus | 1.60E-07 | 15.6509 |
| cadherin binding | 1.60E-07 | 15.6509 |
| positive regulation of cell migration | 4.14E-07 | 14.6980 |
| chromatin | 6.24E-07 | 14.2875 |
| regulation of hydrolase activity | 6.91E-07 | 14.1848 |
| positive regulation of oxidoreductase activity | 7.31E-07 | 14.1288 |
| aging | 7.31E-07 | 14.1288 |
| regulation of positive chemotaxis | 7.31E-07 | 14.1288 |
| positive regulation of positive chemotaxis | 7.31E-07 | 14.1288 |
| immune effector process | 7.31E-07 | 14.1288 |
| defense response to Gram-positive bacterium | 7.31E-07 | 14.1288 |
| regulation of protein kinase activity | 1.11E-06 | 13.7084 |
| activation of caspase activity | 1.33E-06 | 13.5288 |
| positive regulation of DNA metabolic process | 1.33E-06 | 13.5288 |
| DNA damage response, signal transduction | 1.33E-06 | 13.5288 |
| regulation of kinase activity | 1.40E-06 | 13.4805 |
| positive regulation of cell motion | 1.89E-06 | 13.1775 |
| DNA fragmentation involved in apoptosis | 2.55E-06 | 12.8792 |
| negative regulation of NF-kappaB transcription factor activity | 2.55E-06 | 12.8792 |
| fibrillar collagen | 2.55E-06 | 12.8792 |
| positive regulation of nitric oxide biosynthetic process | 2.55E-06 | 12.8792 |
| regulation of nitric-oxide synthase activity | 2.55E-06 | 12.8792 |
| localization within membrane | 2.55E-06 | 12.8792 |
| extrinsic to membrane | 2.55E-06 | 12.8792 |
| regulation of endothelial cell migration | 2.55E-06 | 12.8792 |
| regulation of neuron apoptosis | 2.55E-06 | 12.8792 |
| regulation of blood vessel endothelial cell migration | 2.55E-06 | 12.8792 |
| insulin-like growth factor receptor binding | 2.55E-06 | 12.8792 |
| cell-cell adherens junction | 2.55E-06 | 12.8792 |
| neuron projection development | 2.55E-06 | 12.8792 |
| cellular response to starvation | 2.55E-06 | 12.8792 |
| negative regulation of hydrolase activity | 2.55E-06 | 12.8792 |
| negative regulation of transcription from RNA polymerase II promoter | 2.90E-06 | 12.7497 |
| receptor signaling protein activity | 3.41E-06 | 12.5892 |
| positive regulation of caspase activity | 4.93E-06 | 12.2200 |
| regulation of transferase activity | 5.32E-06 | 12.1437 |
| peptide cross-linking | 7.25E-06 | 11.8344 |
| monooxygenase activity | 7.25E-06 | 11.8344 |
| negative regulation of response to stimulus | 7.25E-06 | 11.8344 |
| positive regulation of protein catabolic process | 7.25E-06 | 11.8344 |
| axon | 7.25E-06 | 11.8344 |
| positive regulation of glucose import | 7.25E-06 | 11.8344 |
| G1 phase | 7.25E-06 | 11.8344 |
| nucleotide-excision repair | 7.25E-06 | 11.8344 |
| response to nutrient levels | 7.25E-06 | 11.8344 |
| response to starvation | 7.25E-06 | 11.8344 |
| protein kinase regulator activity | 8.71E-06 | 11.6515 |
| response to hypoxia | 1.14E-05 | 11.3855 |
| transmembrane receptor protein tyrosine kinase activity | 1.47E-05 | 11.1306 |
| nuclear body | 1.47E-05 | 11.1306 |
| multicellular organismal metabolic process | 1.76E-05 | 10.9473 |
| positive regulation of epithelial cell proliferation | 1.76E-05 | 10.9473 |
| acute inflammatory response | 1.76E-05 | 10.9473 |
| acute-phase response | 1.76E-05 | 10.9473 |
| p53 binding | 1.76E-05 | 10.9473 |
| negative regulation of protein catabolic process | 1.76E-05 | 10.9473 |
| inositol or phosphatidylinositol phosphatase activity | 1.76E-05 | 10.9473 |
| regulation of protein kinase B signaling cascade | 1.76E-05 | 10.9473 |
| SWI/SNF complex | 1.76E-05 | 10.9473 |
| SWI/SNF-type complex | 1.76E-05 | 10.9473 |
| cell aging | 1.76E-05 | 10.9473 |
| regulation of cellular process | 2.06E-05 | 10.7923 |
| response to oxygen levels | 2.37E-05 | 10.6514 |
| cellular process | 2.97E-05 | 10.4246 |
| regulation of cellular metabolic process | 3.49E-05 | 10.2618 |
| NF-kappaB binding | 3.78E-05 | 10.1844 |
| G2/M transition of mitotic cell cycle | 3.78E-05 | 10.1844 |
| response to lipopolysaccharide | 3.78E-05 | 10.1844 |
| kidney development | 3.78E-05 | 10.1844 |
| regulation of transcription factor import into nucleus | 3.78E-05 | 10.1844 |
| gland development | 3.78E-05 | 10.1844 |
| muscle tissue development | 3.78E-05 | 10.1844 |
| ubiquitin protein ligase binding | 3.78E-05 | 10.1844 |
| negative regulation of mitotic cell cycle | 3.78E-05 | 10.1844 |
| base-excision repair | 3.78E-05 | 10.1844 |
| induction of apoptosis by intracellular signals | 3.78E-05 | 10.1844 |
| response to extracellular stimulus | 3.78E-05 | 10.1844 |
| kinase regulator activity | 4.54E-05 | 10.0006 |
| regulation of caspase activity | 5.55E-05 | 9.7997 |
| ossification | 7.33E-05 | 9.5210 |
| activation of phospholipase C activity | 7.33E-05 | 9.5210 |
| positive regulation of phospholipase C activity | 7.33E-05 | 9.5210 |
| adherens junction | 7.33E-05 | 9.5210 |
| nucleus | 7.63E-05 | 9.4806 |
| regulation of signal transduction | 8.33E-05 | 9.3936 |
| regulation of cell communication | 8.33E-05 | 9.3936 |
| regulation of endopeptidase activity | 9.72E-05 | 9.2393 |
| extracellular structure organization | 9.72E-05 | 9.2393 |
| peptidyl-amino acid modification | 0.0001 | 9.0653 |
| response to chemical stimulus | 0.0001 | 9.0194 |
| cell structure disassembly during apoptosis | 0.0001 | 8.9386 |
| apoptotic mitochondrial changes | 0.0001 | 8.9386 |
| cellular component disassembly | 0.0001 | 8.9386 |
| positive regulation of phospholipase activity | 0.0001 | 8.9386 |
| myeloid leukocyte activation | 0.0001 | 8.9386 |
| lamellipodium | 0.0001 | 8.9386 |
| response to endoplasmic reticulum stress | 0.0001 | 8.9386 |
| chaperone binding | 0.0001 | 8.9386 |
| transition metal ion binding | 0.0001 | 8.8140 |
| transmembrane receptor protein kinase activity | 0.0002 | 8.7347 |
| regulation of peptidase activity | 0.0002 | 8.5775 |
| cellular response to hormone stimulus | 0.0002 | 8.5775 |
| response to external stimulus | 0.0002 | 8.4374 |
| structure-specific DNA binding | 0.0002 | 8.4253 |
| platelet activation | 0.0002 | 8.4231 |
| dendrite | 0.0002 | 8.4231 |
| transmembrane receptor protein tyrosine phosphatase activity | 0.0002 | 8.4231 |
| transmembrane receptor protein phosphatase activity | 0.0002 | 8.4231 |
| transcription factor TFIID complex | 0.0002 | 8.4231 |
| intracellular membrane-bounded organelle | 0.0002 | 8.3247 |
| regulation of catalytic activity | 0.0003 | 8.1380 |
| response to stress | 0.0003 | 8.1344 |
| regulation of biological process | 0.0003 | 8.1022 |
| regulation of monooxygenase activity | 0.0003 | 7.9634 |
| protein tetramerization | 0.0003 | 7.9634 |
| localization | 0.0004 | 7.8624 |
| endocytosis | 0.0004 | 7.8624 |
| regulation of metabolic process | 0.0004 | 7.8424 |
| protein amino acid dephosphorylation | 0.0004 | 7.7321 |
| response to stimulus | 0.0004 | 7.7220 |
| response to radiation | 0.0005 | 7.6056 |
| cell differentiation | 0.0005 | 7.5653 |
| regulation of mast cell proliferation | 0.0005 | 7.5507 |
| epidermal growth factor receptor signaling pathway | 0.0005 | 7.5507 |
| positive regulation of phosphorylation | 0.0005 | 7.5507 |
| response to molecule of bacterial origin | 0.0005 | 7.5507 |
| regulation of MAPKKK cascade | 0.0005 | 7.5507 |
| positive regulation of hydrolase activity | 0.0005 | 7.5507 |
| hormone binding | 0.0005 | 7.5507 |
| negative regulation of angiogenesis | 0.0005 | 7.5507 |
| regulation of response to stimulus | 0.0006 | 7.4994 |
| protein amino acid glycosylation | 0.0006 | 7.4827 |
| carbohydrate metabolic process | 0.0006 | 7.3634 |
| transcription factor complex | 0.0006 | 7.3634 |
| cellular response to stimulus | 0.0007 | 7.3151 |
| cytoplasm | 0.0007 | 7.3005 |
| regulation of leukocyte proliferation | 0.0008 | 7.1781 |
| regulation of protein import into nucleus | 0.0008 | 7.1781 |
| negative regulation of MAP kinase activity | 0.0008 | 7.1781 |
| cell adhesion molecule binding | 0.0008 | 7.1781 |
| anchoring junction | 0.0008 | 7.1781 |
| copper ion binding | 0.0008 | 7.1781 |
| positive regulation of lipase activity | 0.0011 | 6.8400 |
| regulation of behavior | 0.0011 | 6.8400 |
| regulation of chemotaxis | 0.0011 | 6.8400 |
| regulation of protein catabolic process | 0.0011 | 6.8400 |
| homophilic cell adhesion | 0.0011 | 6.8400 |
| nucleoplasm | 0.0011 | 6.7891 |
| regulation of DNA metabolic process | 0.0012 | 6.7123 |
| protein modification process | 0.0014 | 6.5609 |
| negative regulation of transcription, DNA-dependent | 0.0014 | 6.5605 |
| oxygen binding | 0.0015 | 6.5316 |
| positive regulation of phosphate metabolic process | 0.0015 | 6.5316 |
| regulation of MAP kinase activity | 0.0015 | 6.5316 |
| positive regulation of adenylatecyclase activity | 0.0015 | 6.5316 |
| PDZ domain binding | 0.0015 | 6.5316 |
| nuclear matrix | 0.0015 | 6.5316 |
| regulation of molecular function | 0.0016 | 6.4103 |
| post-translational protein modification | 0.0016 | 6.4103 |
| biological regulation | 0.0017 | 6.3616 |
| induction of apoptosis | 0.0017 | 6.3551 |
| enzyme linked receptor protein signaling pathway | 0.0017 | 6.3551 |
| intracellular organelle | 0.0018 | 6.3333 |
| regulation of macromolecule metabolic process | 0.0018 | 6.3280 |
| negative regulation of RNA metabolic process | 0.0018 | 6.3053 |
| integrin-mediated signaling pathway | 0.0019 | 6.2492 |
| cell surface binding | 0.0019 | 6.2492 |
| regulation of programmed cell death | 0.0020 | 6.2130 |
| regulation of cell death | 0.0021 | 6.1482 |
| enzyme regulator activity | 0.0021 | 6.1430 |
| intracellular part | 0.0022 | 6.1406 |
| protein complex assembly | 0.0022 | 6.1123 |
| zinc ion binding | 0.0022 | 6.1123 |
| protein stabilization | 0.0025 | 5.9895 |
| regulation of cell migration | 0.0025 | 5.9895 |
| axon guidance | 0.0025 | 5.9895 |
| protein amino acid N-linked glycosylation | 0.0025 | 5.9895 |
| cellular carbohydrate metabolic process | 0.0025 | 5.9735 |
| regulation of nucleocytoplasmic transport | 0.0032 | 5.7498 |
| positive regulation of lyase activity | 0.0032 | 5.7498 |
| androgen receptor binding | 0.0032 | 5.7498 |
| promoter binding | 0.0032 | 5.7498 |
| cellular component organization | 0.0036 | 5.6218 |
| regulation of intracellular transport | 0.0040 | 5.5279 |
| protein localization | 0.0040 | 5.5279 |
| protein dimerization activity | 0.0040 | 5.5233 |
| transmembrane receptor protein tyrosine kinase signaling pathway | 0.0043 | 5.4559 |
| protein binding | 0.0045 | 5.3933 |
| regulation of neurogenesis | 0.0049 | 5.3219 |
| lipid modification | 0.0049 | 5.3219 |
| response to wounding | 0.0051 | 5.2717 |
| regulation of biological quality | 0.0058 | 5.1571 |
| actin filament binding | 0.0059 | 5.1299 |
| macromolecule localization | 0.0059 | 5.1299 |
| chromosomal part | 0.0067 | 4.9992 |
| cell division | 0.0071 | 4.9507 |
| defense response to bacterium | 0.0071 | 4.9507 |
| RasGTPase activator activity | 0.0071 | 4.9507 |
| organ morphogenesis | 0.0076 | 4.8802 |
| vesicle-mediated transport | 0.0080 | 4.8225 |
| mitochondrion organization | 0.0084 | 4.7830 |
| negative regulation of transcription factor activity | 0.0084 | 4.7830 |
| mitotic cell cycle checkpoint | 0.0084 | 4.7830 |
| regulation of cell development | 0.0084 | 4.7830 |
| chromatin remodeling complex | 0.0084 | 4.7830 |
| response to abiotic stimulus | 0.0085 | 4.7659 |
| cytosol | 0.0093 | 4.6811 |
| positive regulation of apoptosis | 0.0095 | 4.6563 |
| cell-cell adhesion | 0.0095 | 4.6558 |
| collagen | 0.0098 | 4.6256 |
| double-stranded DNA binding | 0.0098 | 4.6256 |
| positive regulation of programmed cell death | 0.0111 | 4.5041 |
| positive regulation of transcription from RNA polymerase II promoter | 0.0111 | 4.4982 |
| apoptosis | 0.0111 | 4.4982 |
| nucleolus | 0.0114 | 4.4782 |
| cell-matrix adhesion | 0.0114 | 4.4776 |
| transforming growth factor beta receptor signaling pathway | 0.0114 | 4.4776 |
| regulation of lipase activity | 0.0114 | 4.4776 |
| negative regulation of developmental process | 0.0114 | 4.4776 |
| cell projection | 0.0123 | 4.3978 |
| transcription factor binding | 0.0129 | 4.3490 |
| regulation of gene expression | 0.0131 | 4.3381 |
| regulation of peptidyl-tyrosine phosphorylation | 0.0131 | 4.3381 |
| protein serine/threonine phosphatase activity | 0.0131 | 4.3381 |
| positive regulation of specific transcription from RNA polymerase II promoter | 0.0131 | 4.3381 |
| programmed cell death | 0.0136 | 4.3010 |
| cell part | 0.0136 | 4.2969 |
| growth factor binding | 0.0149 | 4.2065 |
| response to UV | 0.0149 | 4.2065 |
| chromatin remodeling | 0.0149 | 4.2065 |
| androgen receptor signaling pathway | 0.0149 | 4.2065 |
| rRNA processing | 0.0149 | 4.2065 |
| binding | 0.0164 | 4.1127 |
| protein amino acid autophosphorylation | 0.0169 | 4.0821 |
| response to drug | 0.0169 | 4.0821 |
| steroid hormone receptor binding | 0.0169 | 4.0821 |
| receptor binding | 0.0173 | 4.0591 |
| regulation of RNA metabolic process | 0.0173 | 4.0569 |
| regulation of transport | 0.0185 | 3.9875 |
| response to cytokine stimulus | 0.0190 | 3.9642 |
| cell projection organization | 0.0190 | 3.9642 |
| chromatin binding | 0.0190 | 3.9642 |
| single-stranded DNA binding | 0.0190 | 3.9642 |
| cellular developmental process | 0.0195 | 3.9374 |
| cellular protein metabolic process | 0.0211 | 3.8580 |
| cellular localization | 0.0212 | 3.8524 |
| response to bacterium | 0.0212 | 3.8524 |
| negative regulation of protein metabolic process | 0.0212 | 3.8524 |
| anatomical structure formation involved in morphogenesis | 0.0212 | 3.8524 |
| insoluble fraction | 0.0226 | 3.7907 |
| protein transport | 0.0236 | 3.7462 |
| protein kinase cascade | 0.0236 | 3.7462 |
| developmental process | 0.0253 | 3.6774 |
| cytoplasmic part | 0.0260 | 3.6486 |
| regulation of transforming growth factor beta receptor signaling pathway | 0.0261 | 3.6452 |
| cell-substrate adhesion | 0.0261 | 3.6452 |
| response to light stimulus | 0.0261 | 3.6452 |
| positive regulation of MAP kinase activity | 0.0261 | 3.6452 |
| regulation of response to stress | 0.0261 | 3.6452 |
| neuron projection | 0.0261 | 3.6452 |
| regulation of mitotic cell cycle | 0.0288 | 3.5489 |
| integrin binding | 0.0288 | 3.5489 |
| brain development | 0.0288 | 3.5489 |
| cell development | 0.0288 | 3.5489 |
| negative regulation of cell cycle | 0.0288 | 3.5489 |
| tissue development | 0.0294 | 3.5267 |
| cell death | 0.0304 | 3.4920 |
| death | 0.0304 | 3.4920 |
| regulation of protein kinase cascade | 0.0315 | 3.4578 |
| cellular_component | 0.0321 | 3.4392 |
| cation binding | 0.0343 | 3.3740 |
| metal ion binding | 0.0343 | 3.3740 |
| detection of stimulus | 0.0344 | 3.3695 |
| establishment of protein localization | 0.0344 | 3.3695 |
| biological_process | 0.0364 | 3.3128 |
| regulation of protein amino acid phosphorylation | 0.0374 | 3.2856 |
| innate immune response | 0.0374 | 3.2856 |
| chromatin modification | 0.0374 | 3.2856 |
| response to DNA damage stimulus | 0.0374 | 3.2856 |
| ion binding | 0.0405 | 3.2070 |
| regulation of immune response | 0.0405 | 3.2054 |
| regulation of cytokine production | 0.0405 | 3.2054 |
| ATPase activity, coupled to transmembrane movement of substances | 0.0405 | 3.2054 |
| positive regulation of cell proliferation | 0.0432 | 3.1418 |
| regulation of leukocyte activation | 0.0438 | 3.1285 |
| protein tyrosine phosphatase activity | 0.0438 | 3.1285 |
| anion transmembrane transporter activity | 0.0438 | 3.1285 |
| regulation of protein metabolic process | 0.0445 | 3.1126 |
| SH3/SH2 adaptor activity | 0.0471 | 3.0548 |
