# Supplementary material for: Abrogation of PIK3CA or PIK3R1 reduces proliferation, migration, and invasion in glioblastoma multiforme cells
Source: Oncotarget. 2011 Nov 5;2(11):833–49. doi: 10.18632/oncotarget.346 (PMC3260001; doi:10.18632/oncotarget.346)
Supplement: Supplementary file 7 [file oncotarget-02-833-s007.docx]

**Table S6.** Canonical pathways enriched for mutations in GBM according to analysis of the full GBM mutation list (703 genes) in Ingenuity. Canonical pathways that have a p-value of ≤ 0.05 are included in this table.

| **Ingenuity Canonical Pathways** | **p-value** |
| --- | --- |
| Glioma Signaling | 1.202E-08 |
| GlioblastomaMultiforme Signaling | 2.138E-08 |
| Melanoma Signaling | 2.630E-08 |
| Non-Small Cell Lung Cancer Signaling | 9.550E-08 |
| Nitric Oxide Signaling in the Cardiovascular System | 1.122E-07 |
| p53 Signaling | 1.950E-07 |
| Small Cell Lung Cancer Signaling | 3.020E-07 |
| Chronic Myeloid Leukemia Signaling | 3.631E-07 |
| Pancreatic Adenocarcinoma Signaling | 1.318E-06 |
| Endometrial Cancer Signaling | 2.754E-06 |
| Cell Cycle: G1/S Checkpoint Regulation | 5.888E-06 |
| Hereditary Breast Cancer Signaling | 7.244E-06 |
| Molecular Mechanisms of Cancer | 8.318E-06 |
| Myc Mediated Apoptosis Signaling | 1.413E-05 |
| VEGF Signaling | 7.244E-05 |
| Hepatic Fibrosis / Hepatic Stellate Cell Activation | 8.710E-05 |
| HER-2 Signaling in Breast Cancer | 0.000102 |
| Ovarian Cancer Signaling | 0.000105 |
| Amyotrophic Lateral Sclerosis Signaling | 0.000158 |
| EGF Signaling | 0.000166 |
| Prostate Cancer Signaling | 0.000186 |
| IL-4 Signaling | 0.000204 |
| HGF Signaling | 0.000263 |
| FAK Signaling | 0.000355 |
| Bladder Cancer Signaling | 0.000389 |
| SAPK/JNK Signaling | 0.000575 |
| IL-2 Signaling | 0.000741 |
| IL-9 Signaling | 0.000891 |
| PDGF Signaling | 0.000933 |
| FGF Signaling | 0.000955 |
| Estrogen-Dependent Breast Cancer Signaling | 0.001698 |
| PTEN Signaling | 0.001778 |
| Reelin Signaling in Neurons | 0.001862 |
| p70S6K Signaling | 0.001905 |
| iCOS-iCOSL Signaling in T Helper Cells | 0.002042 |
| Integrin Signaling | 0.002239 |
| IGF-1 Signaling | 0.002291 |
| ERK/MAPK Signaling | 0.002399 |
| Aldosterone Signaling in Epithelial Cells | 0.002455 |
| Role of NANOG in Mammalian Embryonic Stem Cell Pluripotency | 0.002570 |
| FcγRIIB Signaling in B Lymphocytes | 0.002692 |
| Docosahexaenoic Acid (DHA) Signaling | 0.002692 |
| Thrombopoietin Signaling | 0.002754 |
| Erythropoietin Signaling | 0.002818 |
| PKCθ Signaling in T Lymphocytes | 0.003162 |
| Prolactin Signaling | 0.003715 |
| Renal Cell Carcinoma Signaling | 0.003715 |
| IL-8 Signaling | 0.003890 |
| TR/RXR Activation | 0.004786 |
| PAK Signaling | 0.004786 |
| HIF1α Signaling | 0.004898 |
| Axonal Guidance Signaling | 0.005495 |
| RANK Signaling in Osteoclasts | 0.005623 |
| Actin Cytoskeleton Signaling | 0.005623 |
| Regulation of eIF4 and p70S6K Signaling | 0.005888 |
| JAK/Stat Signaling | 0.007586 |
| Acute Myeloid Leukemia Signaling | 0.007943 |
| GM-CSF Signaling | 0.008128 |
| Growth Hormone Signaling | 0.008913 |
| Neuropathic Pain Signaling In Dorsal Horn Neurons | 0.009120 |
| CNTF Signaling | 0.009550 |
| Insulin Receptor Signaling | 0.009550 |
| Human Embryonic Stem Cell Pluripotency | 0.010233 |
| AMPK Signaling | 0.010233 |
| Angiopoietin Signaling | 0.010471 |
| EIF2 Signaling | 0.012303 |
| Role of PI3K/AKT Signaling in the Pathogenesis of Influenza | 0.012303 |
| Neurotrophin/TRK Signaling | 0.012303 |
| IL-3 Signaling | 0.014454 |
| Cell Cycle: G2/M DNA Damage Checkpoint Regulation | 0.014454 |
| Renin-Angiotensin Signaling | 0.015136 |
| Rac Signaling | 0.015136 |
| Lymphotoxin β Receptor Signaling | 0.015136 |
| Glioma Invasiveness Signaling | 0.015136 |
| Antiproliferative Role of Somatostatin Receptor 2 | 0.015488 |
| ILK Signaling | 0.016596 |
| Role of Pattern Recognition Receptors in Recognition of Bacteria and Viruses | 0.016596 |
| Sphingosine-1-phosphate Signaling | 0.016982 |
| NF-κB Activation by Viruses | 0.017783 |
| FLT3 Signaling in Hematopoietic Progenitor Cells | 0.017783 |
| Leptin Signaling in Obesity | 0.019055 |
| G-Protein Coupled Receptor Signaling | 0.020417 |
| CTLA4 Signaling in Cytotoxic T Lymphocytes | 0.021878 |
| Germ Cell-Sertoli Cell Junction Signaling | 0.024547 |
| Fc Epsilon RI Signaling | 0.025704 |
| IL-15 Signaling | 0.026303 |
| Calcium Signaling | 0.028184 |
| Aryl Hydrocarbon Receptor Signaling | 0.028840 |
| CD28 Signaling in T Helper Cells | 0.030200 |
| Gα12/13 Signaling | 0.030200 |
| Macropinocytosis Signaling | 0.032359 |
| mTOR Signaling | 0.033113 |
| PI3K/AKT Signaling | 0.034674 |
| Complement System | 0.037154 |
| Role of Macrophages, Fibroblasts and Endothelial Cells in Rheumatoid Arthritis | 0.038019 |
| Role of NFAT in Cardiac Hypertrophy | 0.038019 |
| Cardiac Hypertrophy Signaling | 0.038905 |
| B Cell Receptor Signaling | 0.040738 |
| ATM Signaling | 0.041687 |
| Virus Entry via Endocytic Pathways | 0.041687 |
| LPS-stimulated MAPK Signaling | 0.043652 |
| Relaxin Signaling | 0.044668 |
| NF-κB Signaling | 0.045709 |
| CREB Signaling in Neurons | 0.047863 |
| Neuregulin Signaling | 0.048978 |
| Thrombin Signaling | 0.050119 |

© 2000-2010 Ingenuity Systems, Inc. All rights reserved.
