# Supplementary material for: Abrogation of PIK3CA or PIK3R1 reduces proliferation, migration, and invasion in glioblastoma multiforme cells
Source: Oncotarget. 2011 Nov 5;2(11):833–49. doi: 10.18632/oncotarget.346 (PMC3260001; doi:10.18632/oncotarget.346)
Supplement: Supplementary file 8 [file oncotarget-02-833-s008.docx]

**Table S7.** Canonical pathways enriched for mutations in GBM according to analysis of the stringent GBM mutation list (59 genes) in Ingenuity. Canonical pathways that have a p-value of ≤ 0.05 are included in this table.

| **Ingenuity Canonical Pathways** | **p-value** |
| --- | --- |
| Glioma Signaling | 2.51E-18 |
| Melanoma Signaling | 7.94E-15 |
| Glioblastoma Multiforme Signaling | 2.51E-14 |
| p53 Signaling | 2.51E-13 |
| Pancreatic Adenocarcinoma Signaling | 1.00E-12 |
| Small Cell Lung Cancer Signaling | 1.26E-12 |
| Non-Small Cell Lung Cancer Signaling | 3.16E-11 |
| Molecular Mechanisms of Cancer | 3.16E-11 |
| Ovarian Cancer Signaling | 3.09E-10 |
| Chronic Myeloid Leukemia Signaling | 6.31E-10 |
| Prostate Cancer Signaling | 6.61E-09 |
| Cell Cycle: G1/S Checkpoint Regulation | 2.51E-08 |
| Hereditary Breast Cancer Signaling | 9.33E-08 |
| HER-2 Signaling in Breast Cancer | 1.48E-07 |
| Bladder Cancer Signaling | 3.63E-07 |
| Endometrial Cancer Signaling | 6.92E-07 |
| Myc Mediated Apoptosis Signaling | 1.55E-06 |
| p70S6K Signaling | 2.51E-06 |
| IL-4 Signaling | 2.63E-06 |
| PDGF Signaling | 2.63E-06 |
| Cell Cycle: G2/M DNA Damage Checkpoint Regulation | 7.59E-06 |
| FAK Signaling | 8.51E-06 |
| PTEN Signaling | 1.00E-05 |
| EGF Signaling | 1.12E-05 |
| HGF Signaling | 1.55E-05 |
| HIF1α Signaling | 1.95E-05 |
| iCOS-iCOSL Signaling in T Helper Cells | 2.34E-05 |
| PI3K/AKT Signaling | 4.07E-05 |
| Estrogen-Dependent Breast Cancer Signaling | 4.27E-05 |
| Hepatic Fibrosis / Hepatic Stellate Cell Activation | 5.89E-05 |
| Insulin Receptor Signaling | 6.31E-05 |
| Neurotrophin/TRK Signaling | 6.61E-05 |
| Aryl Hydrocarbon Receptor Signaling | 7.24E-05 |
| LPS-stimulated MAPK Signaling | 7.41E-05 |
| Inositol Phosphate Metabolism | 7.76E-05 |
| Antiproliferative Role of Somatostatin Receptor 2 | 7.76E-05 |
| NF-κB Signaling | 0.000105 |
| IL-9 Signaling | 0.000138 |
| Dendritic Cell Maturation | 0.000162 |
| TR/RXR Activation | 0.000174 |
| PAK Signaling | 0.000174 |
| SAPK/JNK Signaling | 0.000214 |
| Amyotrophic Lateral Sclerosis Signaling | 0.000224 |
| CTLA4 Signaling in Cytotoxic T Lymphocytes | 0.000224 |
| IGF-1 Signaling | 0.000224 |
| FcγRIIB Signaling in B Lymphocytes | 0.000251 |
| Docosahexaenoic Acid (DHA) Signaling | 0.000251 |
| ERK/MAPK Signaling | 0.000251 |
| Sphingosine-1-phosphate Signaling | 0.000372 |
| Integrin Signaling | 0.000380 |
| Role of NANOG in Mammalian Embryonic Stem Cell Pluripotency | 0.000427 |
| PKCθ Signaling in T Lymphocytes | 0.000479 |
| CNTF Signaling | 0.000537 |
| CD28 Signaling in T Helper Cells | 0.000550 |
| Gα12/13 Signaling | 0.000550 |
| Thrombopoietin Signaling | 0.000603 |
| ATM Signaling | 0.000603 |
| IL-2 Signaling | 0.000676 |
| Lymphotoxin β Receptor Signaling | 0.000724 |
| Glioma Invasiveness Signaling | 0.000724 |
| Relaxin Signaling | 0.000724 |
| Huntington's Disease Signaling | 0.000724 |
| Human Embryonic Stem Cell Pluripotency | 0.000851 |
| Colorectal Cancer Metastasis Signaling | 0.000977 |
| CD40 Signaling | 0.000977 |
| IL-15 Signaling | 0.001023 |
| JAK/Stat Signaling | 0.001023 |
| GM-CSF Signaling | 0.001072 |
| Growth Hormone Signaling | 0.001122 |
| B Cell Receptor Signaling | 0.001175 |
| Macropinocytosis Signaling | 0.001175 |
| Erythropoietin Signaling | 0.001230 |
| Angiopoietin Signaling | 0.001230 |
| Role of PI3K/AKT Signaling in the Pathogenesis of Influenza | 0.001349 |
| Prolactin Signaling | 0.001413 |
| Renal Cell Carcinoma Signaling | 0.001413 |
| IL-3 Signaling | 0.001479 |
| Hypoxia Signaling in the Cardiovascular System | 0.001479 |
| Production of Nitric Oxide and Reactive Oxygen Species in Macrophages | 0.001479 |
| Role of Pattern Recognition Receptors in Recognition of Bacteria and Viruses | 0.001585 |
| Clathrin-mediated Endocytosis Signaling | 0.001622 |
| NF-κB Activation by Viruses | 0.001660 |
| IL-17 Signaling | 0.001660 |
| FLT3 Signaling in Hematopoietic Progenitor Cells | 0.001660 |
| Leptin Signaling in Obesity | 0.001738 |
| Reelin Signaling in Neurons | 0.001862 |
| Nitric Oxide Signaling in the Cardiovascular System | 0.001950 |
| Acute Myeloid Leukemia Signaling | 0.001995 |
| Xenobiotic Metabolism Signaling | 0.002042 |
| IL-8 Signaling | 0.002042 |
| Ceramide Signaling | 0.002089 |
| Aldosterone Signaling in Epithelial Cells | 0.002089 |
| Melanocyte Development and Pigmentation Signaling | 0.002344 |
| Role of NFAT in Regulation of the Immune Response | 0.002399 |
| FGF Signaling | 0.002399 |
| EIF2 Signaling | 0.002512 |
| ILK Signaling | 0.002630 |
| VEGF Signaling | 0.002692 |
| Virus Entry via Endocytic Pathways | 0.002754 |
| Breast Cancer Regulation by Stathmin1 | 0.002951 |
| RANK Signaling in Osteoclasts | 0.002951 |
| Neuregulin Signaling | 0.003020 |
| Leukocyte Extravasation Signaling | 0.003020 |
| Thrombin Signaling | 0.003236 |
| HMGB1 Signaling | 0.003311 |
| Fc Epsilon RI Signaling | 0.003631 |
| Neuropathic Pain Signaling In Dorsal Horn Neurons | 0.003631 |
| T Cell Receptor Signaling | 0.003631 |
| Renin-Angiotensin Signaling | 0.004571 |
| Rac Signaling | 0.004571 |
| Actin Cytoskeleton Signaling | 0.004786 |
| Natural Killer Cell Signaling | 0.004786 |
| fMLP Signaling in Neutrophils | 0.004786 |
| Regulation of eIF4 and p70S6K Signaling | 0.004786 |
| CCR3 Signaling in Eosinophils | 0.005248 |
| 14-3-3-mediated Signaling | 0.005495 |
| IL-12 Signaling and Production in Macrophages | 0.005495 |
| Cell Cycle Regulation by BTG Family Proteins | 0.005754 |
| Type II Diabetes Mellitus Signaling | 0.005888 |
| Axonal Guidance Signaling | 0.006457 |
| AMPK Signaling | 0.008913 |
| Role of Oct4 in Mammalian Embryonic Stem Cell Pluripotency | 0.009333 |
| mTOR Signaling | 0.010000 |
| Systemic Lupus Erythematosus Signaling | 0.010471 |
| Germ Cell-Sertoli Cell Junction Signaling | 0.012589 |
| CXCR4 Signaling | 0.013183 |
| Role of BRCA1 in DNA Damage Response | 0.014454 |
| IL-10 Signaling | 0.016218 |
| RAR Activation | 0.016596 |
| Wnt/β-catenin Signaling | 0.016596 |
| CREB Signaling in Neurons | 0.016982 |
| Endothelin-1 Signaling | 0.016982 |
| Acute Phase Response Signaling | 0.017378 |
| NRF2-mediated Oxidative Stress Response | 0.018197 |
| Role of NFAT in Cardiac Hypertrophy | 0.020893 |
| T Helper Cell Differentiation | 0.021380 |
| Role of Macrophages, Fibroblasts and Endothelial Cells in Rheumatoid Arthritis | 0.021380 |
| Basal Cell Carcinoma Signaling | 0.021878 |
| PXR/RXR Activation | 0.022387 |
| LPS/IL-1 Mediated Inhibition of RXR Function | 0.025119 |
| G-Protein Coupled Receptor Signaling | 0.028184 |
| Role of Osteoblasts, Osteoclasts and Chondrocytes in Rheumatoid Arthritis | 0.032359 |
| Cardiac Hypertrophy Signaling | 0.035481 |
| Fcγ Receptor-mediated Phagocytosis in Macrophages and Monocytes | 0.036308 |
| Glucocorticoid Receptor Signaling | 0.047863 |
