# Supplementary material for: Abrogation of PIK3CA or PIK3R1 reduces proliferation, migration, and invasion in glioblastoma multiforme cells
Source: Oncotarget. 2011 Nov 5;2(11):833–49. doi: 10.18632/oncotarget.346 (PMC3260001; doi:10.18632/oncotarget.346)
Supplement: Supplementary file 9 [file oncotarget-02-833-s009.docx]

**Table S8.** Statistically significant invasion-related gene ontologies and pathways enriched for mutations in GBM according to analysis of the full GBM mutation list (703 genes) in Ingenuity and Partek. Gene ontologies generated by Partek are shown in light blue, and canonical pathways generated by Ingenuity are shown in dark blue.

| **Selected Gene Ontologies and Pathways Mutated in GBM** | **p-value** |
| --- | --- |
| extracellular matrix part | 3.09E-09 |
| plasma membrane part | 1.22E-07 |
| collagen | 9.02E-06 |
| collagen binding | 1.88E-05 |
| estrogen receptor signaling pathway | 2.09E-05 |
| regulation of cell-substrate adhesion | 3.16E-05 |
| regulation of focal adhesion formation | 3.16E-05 |
| cytoskeletal anchoring at plasma membrane | 3.16E-05 |
| actin filament binding | 0.000139 |
| EGF Signaling | 0.000166 |
| laminin binding | 0.000202 |
| FAK Signaling | 0.000355 |
| actin binding | 0.000474 |
| fibrinogen complex | 0.000766 |
| ankyrin binding | 0.000766 |
| vinculin binding | 0.000766 |
| PTEN Signaling | 0.001778 |
| extracellular matrix binding | 0.002095 |
| Integrin Signaling | 0.002239 |
| ERK/MAPK Signaling | 0.002399 |
| positive regulation of chemotaxis | 0.004607 |
| HIF1α Signaling | 0.004898 |
| Axonal Guidance Signaling | 0.005495 |
| Actin Cytoskeleton Signaling | 0.005623 |
| negative regulation of cell-matrix adhesion | 0.008594 |
| negative regulation of cell-substrate adhesion | 0.008594 |
| positive regulation of Cdc42 GTPase activity | 0.008594 |
| fibronectin binding | 0.008594 |
| Growth Hormone Signaling | 0.008913 |
| Rac Signaling | 0.015136 |
| ILK Signaling | 0.016596 |
| focal adhesion | 0.022873 |
| positive regulation of cell migration | 0.025724 |
| positive regulation of cell-matrix adhesion | 0.029637 |
| positive regulation of cell-substrate adhesion | 0.029637 |
| structural constituent of cytoskeleton | 0.032812 |
| extracellular matrix structural constituent | 0.033581 |
| PI3K/AKT Signaling | 0.034674 |
| cell-cell adhesion | 0.042758 |
