# Supplementary material for: Abrogation of PIK3CA or PIK3R1 reduces proliferation, migration, and invasion in glioblastoma multiforme cells
Source: Oncotarget. 2011 Nov 5;2(11):833–49. doi: 10.18632/oncotarget.346 (PMC3260001; doi:10.18632/oncotarget.346)
Supplement: Supplementary file 10 [file oncotarget-02-833-s010.docx]

**Table S9.** Statistically significant invasion-related gene ontologies and pathways enriched for mutations in GBM according to analysis of the stringnt GBM mutation list (59 genes) in Ingenuity and Partek. Gene ontologies generated by Partek are shown in light blue, and canonical pathways generated by Ingenuity are shown in dark blue.

| **Selected Gene Ontologies and Pathways Mutated in GBM** | **p-value** |
| --- | --- |
| regulation of cell-substrate adhesion | 3.89E-47 |
| phosphoinositide 3-kinase cascade | 1.98E-39 |
| negative regulation of cell-matrix adhesion | 1.56E-20 |
| negative regulation of cell-substrate adhesion | 1.56E-20 |
| collagen biosynthetic process | 1.56E-20 |
| regulation of cell motion | 6.68E-16 |
| collagen metabolic process | 1.11E-15 |
| negative regulation of focal adhesion formation | 1.11E-15 |
| collagen fibril organization | 2.90E-15 |
| positive regulation of Ras GTPase activity | 9.28E-13 |
| regulation of focal adhesion formation | 9.28E-13 |
| extracellular matrix organization | 2.00E-08 |
| regulation of tissue remodeling | 2.40E-08 |
| cadherin binding | 1.60E-07 |
| positive regulation of chemotaxis | 1.60E-07 |
| positive regulation of cell migration | 4.14E-07 |
| fibrillar collagen | 2.55E-06 |
| cell-cell adherens junction | 2.55E-06 |
| FAK Signaling | 8.51E-06 |
| PTEN Signaling | 1.00E-05 |
| EGF Signaling | 1.12E-05 |
| HIF1α Signaling | 1.95E-05 |
| PI3K/AKT Signaling | 4.07E-05 |
| lamellipodium | 0.0001 |
| epidermal growth factor receptor signaling pathway | 0.0005 |
| cell adhesion molecule binding | 0.0008 |
| anchoring junction | 0.0008 |
| Growth Hormone Signaling | 0.0011 |
| integrin-mediated signaling pathway | 0.0019 |
| ILK Signaling | 0.0026 |
| Rac Signaling | 0.0046 |
| Actin Cytoskeleton Signaling | 0.0048 |
| actin filament binding | 0.0059 |
| Axonal Guidance Signaling | 0.0065 |
| cell-cell adhesion | 0.0095 |
| cell-matrix adhesion | 0.0114 |
| cell projection | 0.0123 |
| cell-substrate adhesion | 0.0261 |
| integrin binding | 0.0288 |
